# A GWAS study highlights significant associations between a series of indels in a *FLOWERING LOCUS T* gene promoter and flowering time in white lupin (*Lupinus albus* L.)

**DOI:** 10.1186/s12870-024-05438-1

**Published:** 2024-07-29

**Authors:** Sandra Rychel-Bielska, Wojciech Bielski, Anna Surma, Paolo Annicchiarico, Jolanta Belter, Bartosz Kozak, Renata Galek, Nathalie Harzic, Michał Książkiewicz

**Affiliations:** 1 Department of Genetics, Plant Breeding and Seed Production, Wroclaw University of Environmental and Life Sciences, Plac Grunwaldzki 24A, Wrocław, 50-363 Poland; 2 Department of Genetics and Plant Breeding, Poznań University of Life Sciences, Dojazd 11, Poznan, 60-632 Poland; 3 Department of Gene Structure and Function, Institute of Plant Genetics, Polish Academy of Sciences, Strzeszynska 34, Poznań, 60-479 Poland; 4 Council for Agricultural Research and Economics, Research Centre for Animal Production and Aquaculture, Viale Piacenza 29, Lodi, 26900 Italy; 5 Cérience, 1 Allée de la Sapinière, Saint Sauvant, 86600 France

**Keywords:** Flowering, Vernalization, Flowering locus T, Promoter, Indel, GWAS, QTL

## Abstract

**Background:**

White lupin (*Lupinus albus* L.) is a high-protein Old World grain legume with remarkable food and feed production interest. It is sown in autumn or early spring, depending on the local agroclimatic conditions. This study aimed to identify allelic variants associated with vernalization responsiveness, in order to improve our knowledge of legume flowering regulatory pathways and develop molecular selection tools for the desired phenology as required for current breeding and adaptation to the changing climate.

**Results:**

Some 120 white lupin accessions originating from a wide range of environments of Europe, Africa, and Asia were phenotyped under field conditions in three environments with different intensities of vernalization, namely, a Mediterranean and a subcontinental climate sites of Italy under autumn sowing, and a suboceanic climate site of France under spring sowing. Two hundred sixty-two individual genotypes extracted from them were phenotyped in a greenhouse under long-day photoperiod without vernalization. Phenology data, and marker data generated by Diversity Arrays Technology sequencing (DArT-seq) and by PCR-based screening targeting published quantitative trait loci (QTLs) from linkage map and newly identified insertion/deletion polymorphisms in the promoter region of the *FLOWERING LOCUS T* homolog, *LalbFTc1* gene (*Lalb_Chr14g0364281*), were subjected to a genome-wide association study (GWAS). Population structure followed differences in phenology and isolation by distance pattern. The GWAS highlighted numerous loci significantly associated with flowering time, including four *LalbFTc1* gene promoter deletions: 2388 bp and 2126 bp deletions at the 5’ end, a 264 bp deletion in the middle and a 28 bp deletion at the 3’ end of the promoter. Besides *LalbFTc1* deletions, this set contained DArT-seq markers that matched previously published major QTLs in chromosomes Lalb_Chr02, Lalb_Chr13 and Lalb_Chr16, and newly discovered QTLs in other chromosomes.

**Conclusions:**

This study highlighted novel QTLs for flowering time and validated those already published, thereby providing novel evidence on the convergence of *FTc1* gene functional evolution into the vernalization pathway in Old World lupin species. Moreover, this research provided the set of loci specific for extreme phenotypes (the earliest or the latest) awaiting further implementation in marker-assisted selection for spring- or winter sowing.

**Supplementary Information:**

The online version contains supplementary material available at 10.1186/s12870-024-05438-1.

## Background

White lupin (*Lupinus albus* L.) is a cool-season grain legume originating putatively from the eastern Mediterranean region, where wild *Greacus*-type accessions are still present [1, 2]. White lupin was primarily domesticated in ancient Greece and Egypt more than three thousand years ago [3]. Its cultivars feature high protein (38–42%) [4] and moderate oil (10–13%) seed content with favorable fatty acid composition for human consumption [5]. White lupin cultivation positively contributes to soil fertility through efficient mobilization of soil phosphorus and – like other legumes – symbiotic nitrogen fixation [6, 7].

White lupin may be cultivated as a spring-sown or an autumn-sown crop. Spring sowing is preferred in colder cropping areas of temperate climate, including also central Europe, whereas autumn sowing is carried out in warmer regions (Australia, the Mediterranean basin, and western Europe). A prolonged cold period during germination and juvenile growth, that induces vernalization, usually accelerates the transition from vegetative to generative growth phase in white lupin landraces, except those adapted to spring sowing and drought escape by rapid flowering [8–10]. A similar observation was done also for two other Old World lupin crop species, narrow-leafed and yellow lupins, where Palestinian accessions gained significant vernalization independence [11–13]. In the latter two species, abolition of vernalization requirements is conferred by large deletions in regulatory regions of one of the four *FLOWERING LOCUS T* (*FT*) homologs present in their genomes: *LanFTc1* in the narrow-leafed lupin, and *LlutFTc1* in the yellow lupin [11, 13, 14]. All three Old World lupin crop species delay flowering under short day photoperiod (as compared to long days). Nevertheless, this response is much more significant in vernalization-requiring accessions than in the thermoneutral genotypes [9, 11, 12, 15–17].

The effect of vernalization on growth and phenology in white lupin is proportional to the amount of cold received by a plant but only within a specific range of temperatures [18]. The lower limit is about 0 °C, whereas the upper is about 12–17 °C [18–20]. Effective vernalization in spring ecotypes occurs relatively quickly, after about 10 days with temperatures up to 12 °C. In contrast, winter types require at least two weeks (optimum 3–4 weeks) with reported temperatures from 1 to 6 °C [8, 9, 20–22]. The timing of flowering is also controlled by the total amount of temperature (growth degree days, GDDs) [22]. Depending on the growth stage, the base temperature is between 0 and 3 °C, with a consensus of 3 °C used for GDDs calculations [18, 19, 22–24]. Flowering occurs after about 500–1000 GDDs, depending on the sowing period (autumn vs. spring), ecotype (winter type or thermoneutral) and fulfillment of vernalization requirements [22]. Vernalization dependence is a key trait for white lupin adaptation to autumn sowing in areas with some freezing temperatures during winter, given the modest frost tolerance of this species [24, 25]. In these areas, moderate or high vernalization requirement prevents flowering before winter frost occurs. Moreover, a positive correlation exists between vernalization requirements and frost tolerance during winter [22]. Vernalization-dependent genotypes also offer some flexibility in selection autumn sowing dates before the onset of winter [18, 22]. However, vernalization dependence is unfavorable under spring sowing, where it unnecessarily delays the crop flowering and maturity in the absence of any risk of frost damage. Moreover, early phenology based on vernalization independence is also an essential component of a drought escape strategy [26–28], which breeders could exploited to cope with the observed substantial increase in warm-season droughts in white lupin cultivation areas. An ecological classification of white lupin genetic resources for European breeding programs highlighted the immense significance of the different phenological types [29]. The long-term shift in climate zones for agriculture is expected to move northward by 500 to 1200 km, creating new opportunities for crop cultivation and expanding acreage for autumn-sown crops [30]. Nevertheless, the higher temperature in winter may result in incomplete fulfillment of vernalization in autumn-sown crops, resulting in shift of floral induction date [31]. This issue can be compensated by developing white lupin cultivars with phenology adapted to local agroclimatic conditions, however, it requires improving our knowledge of heritable components of vernalization responsiveness present in germplasm collections as well as the development of tools for molecular selection of the desired (early, intermediate or late) phenology.

To facilitate studies on the genetic inheritance of domestication-related traits in white lupin, a recombinant inbred line (RIL) mapping population was developed from a cross between the early flowering vernalization-independent cultivar Kiev Mutant from Ukraine and the late flowering vernalization-responsive landrace P27174 from Ethiopia [32]. Quantitative trait loci (QTL) mapping in this RIL population revealed the presence of several QTLs for time to flowering [32–35]. Polymorphic loci from the genetic map were transformed into PCR-based markers and used for correlation analysis in germplasm collection carrying domesticated and wild accessions [36]. Although that study confirmed the significance of major QTLs from the RIL population, it highlighted the lack of loci associated with the most contrasting phenotypes (very early or very late). Recently, based on the constructed high-density consensus linkage map [34], two chromosome-scale genome sequences were established [37, 38], supplemented with a pangenome assembly carrying 40 accessions [39]. These resources have enabled us to conduct genome-wide association study (GWAS) addressing phenological diversity in white lupin germplasm collection carrying genotypes from three main climate zones. Recently, GWAS was used in white lupin to highlight significant SNPs associated with anthracnose resistance, seed alkaloids, protein content, yield under moist and dry conditions, and onset of flowering [28, 40, 41].

The study aimed to find genetic variants linked to vernalization independence or responsiveness for breeding and adaptation to climate change. The germplasm diversity panel included 262 white lupin genotypes that were subjected to genotyping by whole-genome Diversity Arrays Technology sequencing (DArT-seq) and PCR-based screening of published flowering time QTLs and novel insertion/deletion (indel) polymorphisms found in the regulatory region of white lupin *FTc1* gene homolog, *LalbFTc1*. Obtained molecular data were used for association analysis encompassing greenhouse observations in Poland under ambient long-day photoperiod (spring sowing without vernalization) and field observations collected in three test environments representing diverse agroclimatic conditions and intensity of vernalization, i.e. Mediterranean and subcontinental climate regions in Italy (autumn sowing) and suboceanic climate in France (spring sowing).

## Results

### Phenotyping of the white lupin diversity panel revealed high variability in flowering time and vernalization-responsiveness

The white lupin diversity panel [10] originating from three main climate zones (tropical, subtropical and temperate) [42, 43] was evaluated in four environments: spring sowing in greenhouse with absolute lack of vernalization under long day photoperiod (Poznań) as well as in field conditions by autumn sowing with strong vernalization (Lodi), autumn sowing with moderate vernalization (Sanluri) and spring sowing with mild vernalization (Saint Sauvant).

Time from sowing to the start of flowering significantly differed between environments (Supplementary File S1). As expected, it was the longest in autumn sowing, ranging from 173 to 210 days in Lodi and from 89 to 170 days in Sanluri, followed by spring sowing in Saint Sauvant, ranging from 64 to 106 days, and spring sowing in a greenhouse in Poznań, ranging from 41 to 127 days in 2020 and from 41 to 121 days in 2021. As in greenhouse surveys we counted also time from sowing to bud emergence and to end of flowering, it was possible to compare broad sense heritability for these three traits in this environment. It reached the highest value for days to bud emergence (0.968) and days to start of flowering (0.967), followed by days to end of flowering (0.956). It should be noted that average standard deviation of flowering time within accessions in greenhouse reached 2.3 days, whereas for the whole dataset in this environment it was 15.9 days. It indicates that variability in flowering time between accessions in controlled environments was significantly higher than between genotypes within accessions. The correlation of flowering time between greenhouse and field-based phenotyping was the highest for spring sowing in Saint Sauvant (0.89), followed by autumn sowing in Sanluri (0.88) and in Lodi (0.60). All values were statistically significant, nevertheless lower value of correlation between greenhouse and Lodi reflects the influence of vernalization-responsiveness for flowering time in white lupin (see Discussion). Despite thermal differences between environments, the earliest genotypes in Lodi were also very early flowering in other environments, and similar consistency was found for very late genotypes. Indeed, fourteen genotypes from Turkey, three from Syria and one from Jordan revealed rapid flowering irrespective of the length and severity of vernalization. Total photoperiod (daylight) hours from sowing to the start of flowering (Supplementary File S2) ranged from 522.6 to 2247.9 h. It was the lowest in greenhouse (mean value 984.4 h) and the highest in Lodi (mean value 1741.8 h). This trait was highly correlated (as expected) with the number of days from sowing to the start of flowering in all environments (r value above 0.999). The cumulative number of growing degree days from sowing to start of flowering (GDDs) (Supplementary File S3) was the lowest in Saint Sauvant (517.5-1100.3), followed by Lodi (650.5–1048.0), Sanluri (816.0-1435.2), whereas the highest in greenhouse (725.7-2396.6 in 2020 and 698.1-2282.9 in 2021). Differences between environments in GDDs were the highest for vernalization-dependent accessions. Cumulative vernalization effectiveness of daily temperature (VF) significantly differed between environments, reaching at flowering stage 0 days in greenhouse (no pre-sowing vernalization), 18.23–18.67 days in Saint Sauvant, 18.11–55.73 days in Sanluri, and 117.27-121.97 days in Lodi. The number of accessions that initiated flowering before the end the vernalization period ranged from 18 in Saint Sauvant and 33 in Lodi to 158 in Sanluri (Supplementary File S4).

### DArT-seq genotyping of white lupin diversity panel provided 6735 high-quality markers

DArT-seq protocol [44, 45] executed for 262 genotypes yielded 16,713 polymorphic presence/absence (dominant) markers (SilicoDArTs) and 11,506 single-nucleotide polymorphism (SNP) markers. SilicoDArT markers achieved an average read depth of 32.8 with an average reference genome alignment e-value of 4.2E-09. Taking into consideration SNP markers, the mean read count was 21.3 for the reference allele and 16.8 for the alternative allele, with an average reference genome alignment e-value of 1.4E-09, whereas the mean genotype call count per line was 10,096, with values ranging from 7266 (LAP106d) to 10,775 (LAP047b). After missing data imputation, duplicated loci removal and minor allele frequency (MAF) filtering, 4971 SNP and 1764 SilicoDArT markers were retained for further analysis (Supplementary File S5).

### Screening of the white lupin diversity panel with PCR markers for QTLs from the linkage map identified rare LalbFTa1 gene indel

The analysis of fifteen PCR-based markers (Table 1), associated with QTLs for flowering time derived from the linkage mapping studies [35, 36], revealed the polymorphism in the set of 262 white lupin genotypes for all markers except TP47110. This marker yielded an alternative allele only in a control sample (P27174 landrace). Two other markers, FTa1-F2 and SKIP1-F2, revealed very low MAF values (1.5% and 3.0%, respectively). The FTa1-F2 marker recognizes a deletion localized in the third intron from one of the four *Arabidopsis thaliana FT* homologs present in the white lupin genome, *LalbFTa1* gene (*Lalb_Chr02g0156991*). This marker co-segregated in mapping studies with one major QTL for flowering time in a RIL population descending from Ethiopian parent germplasm [34, 35]. In the present study, the Ethiopian *LalbFTa1* allele was found only in four genotypes, three originating from Ethiopia (LAP079c, LAP084d and LAP084c) and one from Italy (LAP098a). These genotypes differed in phenology. Namely LAP079c was quite early flowering and low-responsive to vernalization, whereas the remaining showed delayed flowering without severe vernalization. Interestingly, other tested Ethiopian germplasm (6 genotypes) carried the reference *LalbFTa1* allele and were rather early flowering in all studied environments. The other major QTL marker with low MAF value, SKIP1-F2, targets non-synonymous (H to P) SNP locus (A to C) present in the coding sequence of F-box protein SKIP1 (*Lalb_Chr13g0300781*). Eight accessions that carried this mutation originated from 6 countries (Egypt, France, Italy, Portugal, Turkey, and Jordan) and showed high variability in flowering time in a controlled environment (greenhouse) and Sanluri, suggesting a possible lack of association between SKIP1-F2 allele and vernalization-independent flowering. Agarose gel electrophoregrams showing polymorphism of PCR-based markers from flowering time QTLs are provided in Supplementary File S6.

**Table 1:** PCR-based markers tagging four major QTLs for flowering time from linkage mapping studies [34–36] and their minor allele frequency (MAF) values in the white lupin diversity panel. SNPs were resolved by the cleaved amplified polymorphic sequence (CAPS) [46] or derived CAPS (dCAPS) [47] approaches. For simple PCR markers amplicon lengths are provided, whereas for CAPS and dCAPS markers – the lengths of restriction products

Marker name	Chrom.	Start position (bp)	End position (bp)	Polymorphism detection	Products Kiev Mutant (pb)	Products P27174 (bp)	MAF (%)
QTL01 MFT-FT3-F1	Chr05	6 316 914	6 316 936	PCR	295	311	18.3
QTL02 FTc1-F4	Chr14	5 849 993	5 850 017	PCR	298	291	20.6
QTL03 FY-F6	Chr01	4 477 992	4 478 013	CAPS, *Tsp*45I	233, 202	435	10.9
QTL05 VIP3-F2	Chr07	2 121 666	2 121 685	CAPS, *BspL*I	114, 85, 70	184, 85	15.1
QTL06 TP23903	Chr02	152 571	152 592	CAPS, *Bse*GI	64	49, 15	39.3
QTL07 TP235608	Chr02	14 541 014	14 541 035	CAPS, *Afl*II	217	179, 38	45.4
QTL08 TP94353	Chr02	15 049 541	15 049 562	CAPS, *Rsa*I	60, 51	111	23.7
QTL09 SKIP1-F2	Chr13	13 924 778	13 924 804	dCAPS, *Bse*DI	79	48, 31	3.8
QTL10 TP402859	Chr13	15 203 335	15 203 355	CAPS, *Hpa*II	198	112,86	27.1
QTL11 FTa1-F2	Chr02	15 000 158	15 000 178	PCR	2218	1535	1.5
QTL12 SEP3-F1	Chr13	15 042 385	15 042 404	dCAPS, *Taq*I	122, 23	145	41.6
QTL13 TP86766	Chr16	453 954	453 971	CAPS, *Dde*I	64	48, 16	41.2
QTL14 PIF4-F6	Chr04	1 105 241	1 105 263	CAPS, *Hpy*188III	138, 52	102, 52, 36	31.7
QTL15 TP47110	Chr02	635 858	635 884	CAPS, *Hpy*F3I	42, 24	66	0.0
QTL16 TP345457	Chr13	14 690 123	14 690 147	CAPS, *Bse*DI	227, 39, 12	143, 84, 39, 12	19.3

### White lupin pangenome alignment unveiled a series of indels in the LalbFTc1 gene promoter

Recent studies on the narrow-leafed and yellow lupin genomes highlighted the association between insertion-deletion (indel) polymorphism in the promoter region of *FTc1* genes (*LanFTc1* and *LlutFTc1*, respectively) and vernalization-independent early flowering [11–14]. Therefore, we investigated the potential structural polymorphism of the orthologous *FTc1* region in white lupin. Multiple alignment of genome sequences derived from 40 white lupin accessions [39] revealed a complex pattern of indel polymorphism in the regulatory region of the *LalbFTc1* gene, *Lalb_Chr14g0364281* (Table 2). Considering cv. Amiga genome as a reference [38], seven deletions and two insertions were identified. Two short deletions (7 bp and 28 bp) were found in the region localized about 800 bp upstream of the transcription start site (TSS). Another two short and one longer deletions (5 bp, 24 bp and 264 bp) and one short insertion (25 bp), were identified in the region localized 3.9–4.9 kbp upstream of the TSS. Between these two regions, a remarkably long (3936 bp) insertion was found at ~ 2.5 kbp upstream of the TSS, raising a question about the length of the functional *LalbFTc1* promoter to be considered in polymorphism analysis of genotypes carrying this insertion. In the most external region, localized 5.6-8.0 kbp upstream of the TSS, two overlapping large deletions (2388 bp and 2126 bp) were localized, having the same distal starting point (Table 2).

**Table 2:** Structural variants identified in the *LalbFTc1* gene (*Lalb_Chr14g0364281*) promoter in the white lupin pangenome sequence alignment [39]

Name	Min distance to TSS (bp)^1^	Max distance to TSS (bp)^1^
LD37 deletion, 2388 bp	-7 996	-5 609
P27174 deletion, 2126 bp	-7 996	-5 871
GR38 deletion, 24 bp	-4 915	-4 892
LAP022E insertion, 25 bp	-4 499	-4 499
LAP022E deletion, 264 bp	-4 408	-4 145
LAP022E deletion, 5 bp	-3 936	-3 932
P27174 insertion, 3818 bp	-2 495	-2 495
LAP029B deletion, 7 bp	-858	-852
LAP029B deletion, 28 bp	-780	-753
*LalbFTc1* TSS	0	0
*LalbFTc1* exon1	47	244
*LalbFTc1* exon2	356	417
*LalbFTc1* exon3	623	663
*LalbFTc1* exon4	2 310	2 533
*LalbFTc1* PolA	2 553	2 553

^1^ position relative to the transcription start site of the *LalbFTc1* gene (Lalb_Chr14, 5,850,617 bp, direction “forward”)

### PCR-based screening evidenced the presence of several LalbFTc1 gene promoter indel variants in the white lupin diversity panel

The set of 18 PCR primer pairs was designed and successfully implemented for screening 262 white lupin genotypes towards indel polymorphism and selected SNP polymorphism in the regulatory region of the *LalbFTc1* gene (Table 3). As some primer pairs generated more than two alleles corresponding to the different lengths of deletions, several scoring schemes were implemented for these products to maintain bi-allelic segregation in the GWAS dataset, and such markers were named with a, b, c and d suffixes (i.e., markers PR_58a-c, 35a-b, 36a-b, 42a-b and 71a-d). In total, 26 PCR-based markers targeting polymorphism in the *LalbFTc1* gene promoter were analyzed in the white lupin diversity panel. Only one marker (PR_43) turned out to be monomorphic in the analyzed set of genotypes. However, another ten markers (PR_32, PR_34, PR_35a, PR_36a, PR_61, PR_37, PR_38, PR_40, PR_42b, PR_71c) revealed MAF values below 5%. Consequently, they were excluded from population structure analysis and further calculations. Moreover, two other markers, PR_30 and PR_31, revealed identical segregation pattern, so only PR_30 was retained. Observed patterns of polymorphism confirmed all indel variants extracted from pangenome sequence alignment. They also designated a novel large candidate (above 6 kbp) deletion in the *LalbFTc1* gene promoter region of three Turkish genotypes LAP027a, LAP027b and LAP027c, highlighted by the lack of amplification of any PCR product from markers localized between about 1 and 8 kbp upstream of the TSS. The detected polymorphism for the studied PCR-based markers for *LalbFTc1* gene promoter indels is visualized on sample agarose gel electrophoregrams in Supplementary File S7.

**Table 3:** Markers used for PCR-based genotyping of indel polymorphism in the *LalbFTc1* gene (*Lalb_Chr14g0364281*) promoter and their minor allele frequency (MAF) in white lupin diversity panel

Name	Min distance to TSS (bp)^1^	Max distance to TSS (bp)^1^	Score “0”	Score “1”	Score “2”	MAF (%)
PR_30	-8 065	-7 254	812 bp	-	no product	14.9
PR_58a	-8 065	-5 562	2504 bp	heterozygote	378 bp	13.5
PR_58b	-8 065	-5 562	2504–378 bp	-	no product or 116 bp	7.3
PR_58c	-8 065	-5 562	2504–378 bp or no product	-	116 bp	20.6
PR_31	-7 312	-6 553	760 bp	-	no product	14.9
PR_32	-6 662	-6 060	589 bp	-	no product	0.5
PR_33	-6 242	-5 562	681 bp	-	no product	10.0
PR_34	-5 694	-4 899	796 bp	-	no product	4.2
PR_35a	-4 968	-4 320	649 bp, no product	heterozygote	625 bp	5.2
PR_35b	-4 968	-4 320	649 bp	-	625 bp, heterozygote or no product	9.5
PR_61	-4 968	-3 254	1715 bp	-	no product	2.1
PR_36a	-4 585	-3 860	726 bp or heterozygote	-	482 bp or no product	3.4
PR_36b	-4 585	-3 860	726 bp or no product	-	482 bp or heterozygote	20.6
PR_37	-3 965	-3 254	712 bp	-	no product	1.6
PR_38	-3 403	-2 695	709 bp	-	no product	1.6
PR_39	-2 908	-2 262	no product	-	647 bp	13.4
PR_40	-2 365	-1 591	775 bp	-	no product	1.0
PR_41	-1 944	-1 179	no product	-	766 bp	18.7
PR_42a	-1 259	-451	802–809 bp	-	774–781 bp	8.2
PR_42b	-1 259	-451	other variants	-	~ 850 bp	3.1
PR_71a	-970	-714	222 or 229 or 250 bp	heterozygote	257 bp	11.3
PR_71b	-970	-714	250–257 bp	heterozygote	222–229 bp	8.0
PR_71c	-970	-714	other variants	-	~ 280 bp	3.8
PR_71d	-970	-714	250 bp	-	other variants	21.0
PR_70	-875	-600	no product	-	276 bp	12.2
PR_43	-560	140	701 bp	-	no product	0.0

^1^ position relative to the transcription start site of the *LalbFTc1* gene (Lalb_Chr14, 5,850,617 bp, direction “forward”)

### Population structure analysis of the white lupin diversity panel uncovered associations with phenology and geographic origin

In total 6765 markers, including 13 QTL-based PCR markers, 17 *LalbFTc1* gene indel PCR markers, 4971 SNP and 1764 SilicoDArT markers were used for population structure, principal component analysis (PCA) and GWAS calculations (Supplementary File S5). The mean heterozygosity of markers was 4.7%, ranging from 0 to 61.8%. The heterozygosity of genotypes ranged from 0.5 to 16.2%. Within the investigated germplasm panel, 74 genotypes had heterozygosity below 1%, whereas 39 genotypes above 10%. This result was largely expected, as white lupin is a predominantly inbred crop with a cross-pollination rate of about 10% [48]. Analysis of cross-entropy graph for clustering within the range from K2 to K30 revealed two major inflection points at K = 5 and K = 12 and a cross-entropy minimum at K = 17 (Supplementary File S8). K-values within the range of 4–8 were screened for population structure analysis (Supplementary File S9).

**Fig. 1:**
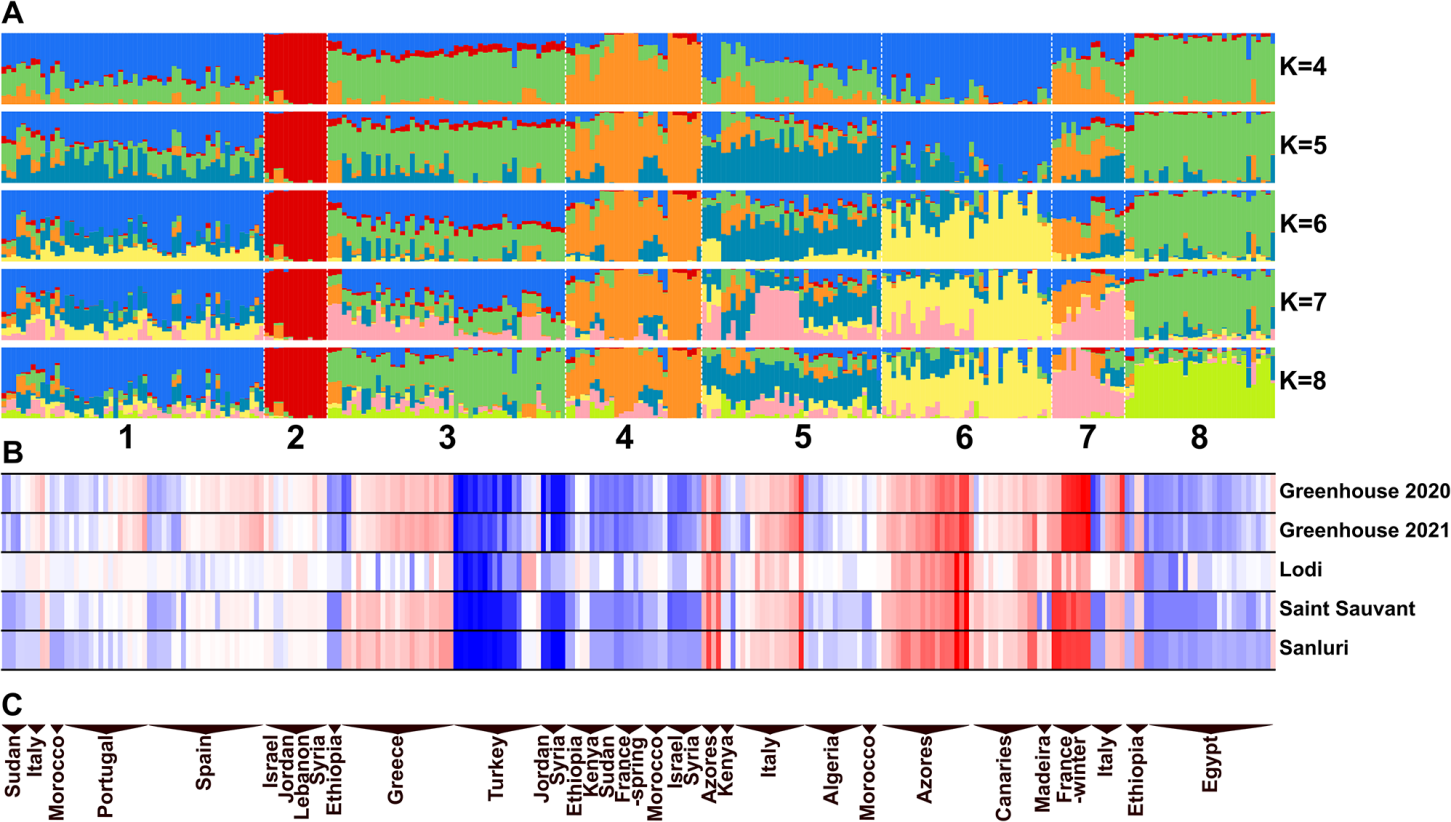
Summary of population structure analysis of 262 white lupin genotypes. The panels show STRUCTURE diagrams under different K-values (A), mean total number of growing degree days (GDDs) from sowing to flowering observed in 5 environments (B), and geographic localization of germplasm samples (C). Environments are as follows: spring sowing in controlled conditions with an absolute lack of vernalization (Greenhouse 2020 and 2021), autumn sowing in field conditions with strong vernalization (Lodi) and moderate vernalization (Sanluri) and spring sowing in field conditions with mild vernalization (Saint Sauvant). In total, 6765 markers were used for population structure analysis. Different colors mark major clusters for different K-grouping scenarios. Clustering at K8 is indicated by vertical lines and numbers from 1 to 8. GDDs were visualized by scale from blue (minimum value for the environment) through white (mean value) to red (maximum value)

**Fig. 2:**
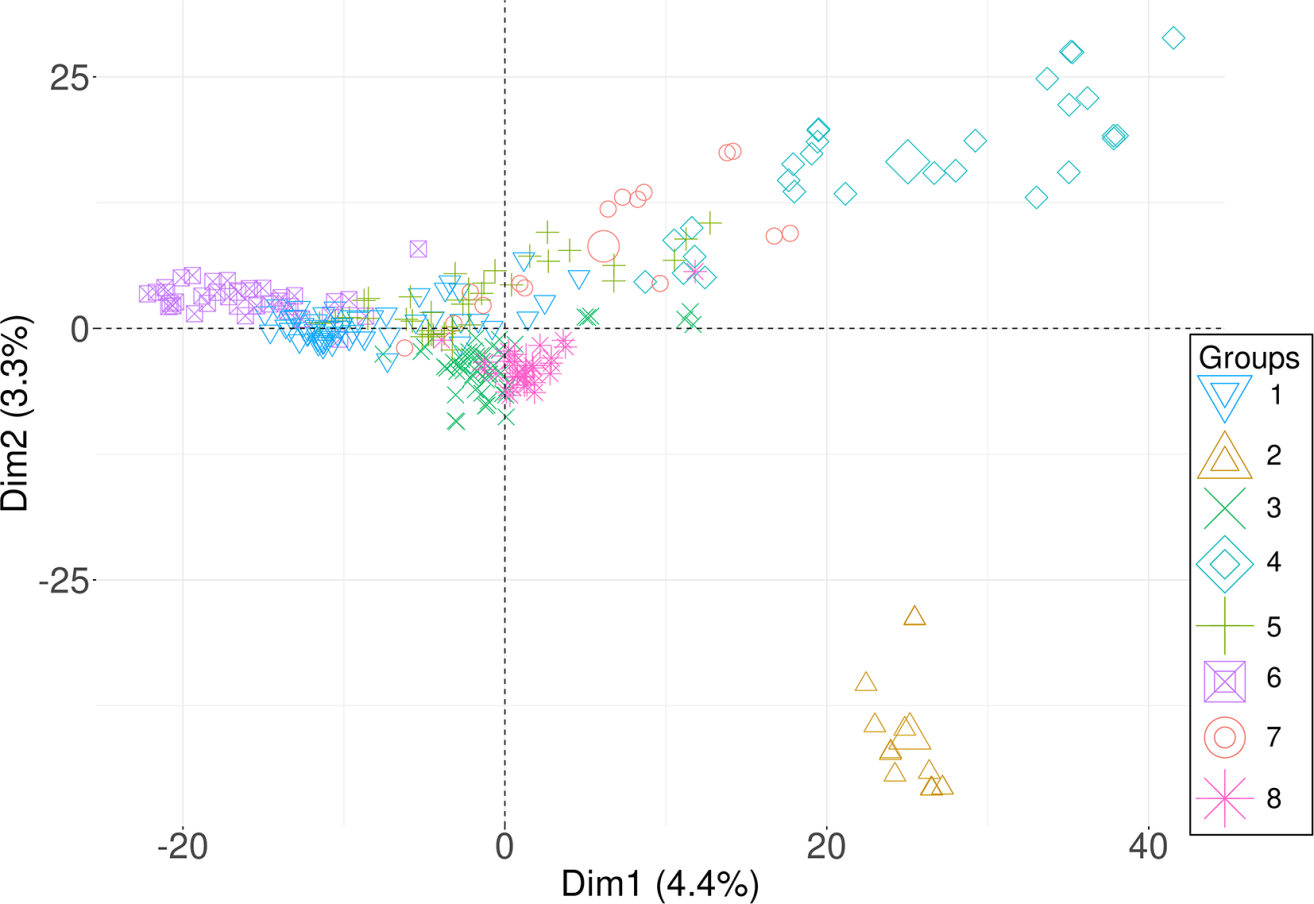
The principal component analysis (PCA) showing separation of major clusters of white lupin genotypes formed at K8. In total, 6765 markers were used for population structure analysis

Since the first tested K-value (K4), grouping started to follow differences in flowering time and geographical origin of accessions (Fig. 1). Two genotypes from Syria, seven from Lebanon, two from Israel and two from Jordan formed a cluster (No. 2) that was perfectly stable in all tested K-values. Genotypes within this cluster had very similar phenology, highlighted by low standard deviation of flowering time in all tested environments (0.6 to 4.1 days). The time to flowering of this cluster was close to the mean value observed in these environments. The separation of this cluster from other genotypes was also highlighted by the most distant position on the plot showing the first two principal components (Fig. 2).

Another relatively stable cluster (No. 4) was formed at K4 from a few genotypes originating from each of the East African resources (Ethiopia, Kenya and Sudan), Egypt, Maghreb (Morocco) and West Asia (Israel and Syria) as well as all France cultivars (both spring-type and winter-type) and one Italian genotype (Fig. 1). That cluster can be found on the distant right position on the PCA graph supporting its significant separation from the other groups (Fig. 2). This cluster revealed very high variability in flowering time, and, consequently, at higher K-values it divided into two clusters (No. 4 and 7) that differred by phenology.

A set of 26–27 genotypes from Egypt was initially grouped together with a large amount of Greek, Italian, and Turkish germplasm (cluster No. 3). However, as the analysis progressed, this association diminished at higher K-values, and the genotypes were ultimately divided into three clusters: the first consisted of Egyptian genotypes characterized by early phenology, the second one mostly comprised Italian genotypes and the third included late flowering, vernalization-responsive Greek germplasm, along with very rapid flowering and thermoneutral Turkish, Jordanian, and Syrian genotypes. (Fig. 1). These three clusters can be found at overlapping positions in the middle section of the PCA graph (Fig. 2).

Most Azorean genotypes grouped together with Madeira & Canaries germplasm across all analyzed K-values. This cluster (No. 6) revealed late or very late flowering time in all tested environments and localized at the left side of the PCA (Fig. 2). Spanish and Portuguese accessions formed a cluster with a few Maghreb (Morocco) and Italian genotypes. This group revealed intermediate flowering time with low variability between accessions. Five Ethiopian genotypes (from nine analyzed) clustered together across all tested K-values and revealed quite early flowering phenotype in all environments except Lodi.

### Newly discovered ***LalbFTc1*** indels revealed significant associations with flowering time in white lupin diversity panel

**Fig. 3:**
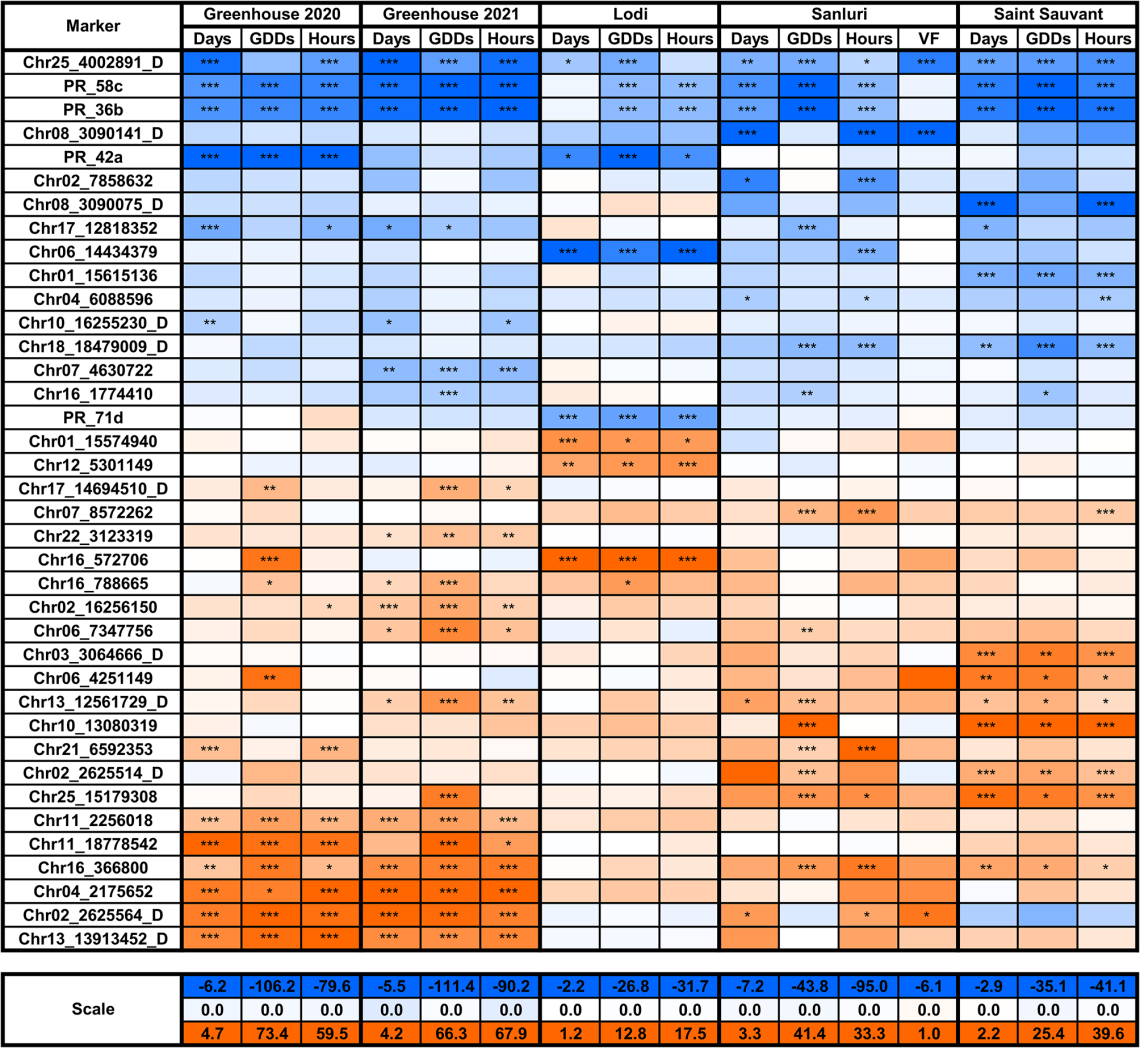
Results of genome-wide association study (GWAS) in white lupin diversity panel. The K = 5 was selected as the representative number of clusters for population structure. In total, 6765 markers obtained for 262 genotypes were analyzed. Only markers that revealed significant associations in at least three environment × trait combinations are shown. Flowering time was observed in 5 environments: spring sowing in controlled conditions with absolute lack of vernalization (Greenhouse 2020 and 2021) and in field conditions - autumn sowing with strong (Lodi) or moderate vernalization (Sanluri), and spring sowing with mild vernalization (Saint Sauvant). Trait abbreviations are as follows: GDDs, the number of growing degree days; Days, the number of calendar days; Hours, the total number of photoperiod hours; VF, the cumulative vernalization effectiveness of daily temperature. All traits were counted from the sowing date until the start of flowering. Markers were sorted from the negative to the positive effect of an alternative allele on the number of days to flowering. Colors indicate the impact and direction of effects according to minimum (blue), zero (white) and maximum (red) values provided on the scale. Asterisk (*) indicates Benjamini-Hochberg false discovery rate (FDR)-adjusted P-value in the following scheme: ***, *p* < 0.0001; **, 0.0001 ≤ *p* < 0.001; *, 0.001 ≤ *p* ≤ 0.05

**Fig. 4:**
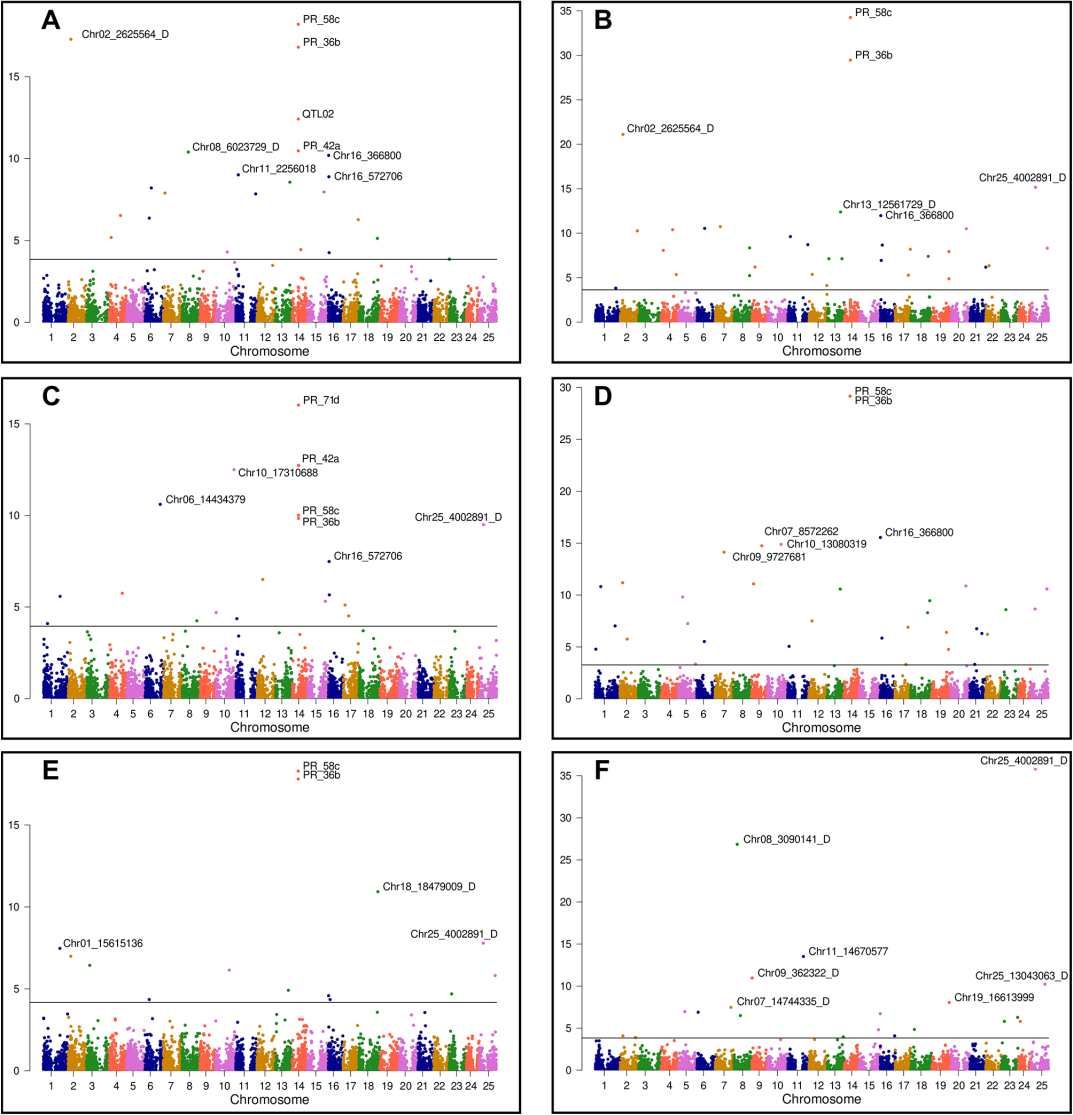
Manhattan plots for genome-wide association study (GWAS) in the white lupin diversity panel. The panels show the cumulative number of growing degree days in Greenhouse 2020 (A), Greenhouse 2021 (B), Lodi (C), Sanluri (D), Saint Sauvant (E) and the cumulative vernalization effectiveness of daily temperature in Sanluri (F). K = 5 was selected as the representative number of clusters for population structure. In total 6765 markers and 262 genotypes were analyzed. P-values are presented on y axis and chromosome positions on x axis. Benjamini-Hochberg false discovery rate (FDR)-adjusted P-value < 0.05 corresponds to the threshold indicated on the graphs as a horizontal line. For the sake of clarity, names were shown only for a few most significant markers

Based on the PCA and cross-entropy analysis, K = 5 was selected as the representative number of clusters for population structure in GWAS. Five environments (Greenhouse 2020, Greenhouse 2021, Lodi, Sanluri, and Saint Sauvant) and four traits counted from sowing to start of flowering were analyzed: the number of days, the cumulative number of growing degree days (GDDs), the total photoperiod hours and the cumulative vernalization effectiveness of daily temperature (VF). As the temperature in the greenhouse was always above the vernalization threshold, 18 environment × trait combinations were included in GWAS. The set of 195 markers revealed at least one marker-trait association with false discovery rate (FDR)-corrected P-value below the 0.05 threshold (Supplementary File S10), including 38 markers significantly associated in at least three environment × trait combinations (Table 4, Fig. 3). This set included, among others, four PCR markers recognizing *LalbFTc1* promoter indels: 2388 bp and 2126 bp deletions at the 5’ end of the promoter (marker PR_58c), a 264 bp deletion in the middle region (marker PR_36b) and a 28 bp deletion at the 3’ end of the promoter (markers PR_42a and PR_71d) (Figs. 3 and 4).

**Table 4:** Markers that revealed marker-trait associations in the white lupin genome-wide association study (GWAS) with false discovery rate corrected P-value below the 0.05 threshold in at least three environment × trait combinations. The K = 5 was selected as the representative number of clusters for the population structure

Marker	Chromosome	Position	MAF^1^	*P* < 0.05^2^	GDDs^3^	Days^3^	Hours^3^	VF^3^
PR_58c	Chr14	5,842,853	20.6	14	-57.8	-3.3	-48.2	-0.2
PR_36b	Chr14	5,846,133	20.6	14	-55.3	-3.2	-48.5	-0.2
Chr25_4002891_D	Chr25	4,002,891	20.6	14	-34.0	-3.5	-38.1	-1.7
Chr16_366800	Chr16	366,800	17.9	11	33.3	1.7	26.8	0.2
Chr02_2625564_D	Chr02	2,625,564	43.7	9	23.4	1.9	24.3	0.3
Chr13_12561729_D	Chr13	12,561,729	35.1	8	21.5	1.1	13.2	0.2
PR_42a	Chr14	5,849,359	8.2	7	-31.4	-1.9	-28.8	-0.3
Chr04_2175652	Chr04	2,175,652	6.9	6	29.4	1.9	33.1	0.3
Chr11_2256018	Chr11	2,256,018	38.5	6	20.8	1.3	16.9	0.1
Chr13_13913452_D	Chr13	13,913,452	9.2	6	26.2	2.0	28.5	0.1
Chr17_12818352	Chr17	12,818,352	13.0	6	-18.8	-1.5	-16.4	0.0
Chr25_15179308	Chr25	15,179,308	11.8	6	26.4	1.2	16.6	0.2
Chr06_4251149	Chr06	4,251,149	8.8	5	20.1	1.1	8.4	0.4
Chr11_18778542	Chr11	18,778,542	8.4	5	29.8	1.6	22.6	0.0
Chr18_18479009_D	Chr18	18,479,009	30.0	5	-15.5	-0.8	-16.3	-0.2
Chr02_2625514_D	Chr02	2,625,514	44.1	4	16.1	1.2	15.8	-0.2
Chr02_16256150	Chr02	16,256,150	46.0	4	14.2	1.0	11.3	0.1
Chr06_7347756	Chr06	7,347,756	18.1	4	20.9	1.0	11.6	0.1
Chr06_14434379	Chr06	14,434,379	6.3	4	-9.4	-1.1	-16.6	-0.1
Chr08_3090075_D	Chr08	3,090,075	8.0	4	-10.8	-1.5	-19.3	-0.5
Chr10_13080319	Chr10	13,080,319	7.3	4	17.5	1.1	17.1	-0.1
Chr16_572706	Chr16	572,706	23.3	4	19.1	0.8	8.2	0.2
Chr16_788665	Chr16	788,665	22.5	4	16.6	0.9	12.7	0.1
Chr21_6592353	Chr21	6,592,353	35.9	4	10.5	1.2	18.2	0.2
Chr01_15574940	Chr01	15,574,940	21.9	3	4.6	0.2	10.6	0.1
Chr01_15615136	Chr01	15,615,136	22.9	3	-7.7	-1.0	-12.1	-0.1
Chr02_7858632	Chr02	7,858,632	5.2	3	-7.4	-1.9	-22.7	-0.4
Chr03_3064666_D	Chr03	3,064,666	9.5	3	8.6	1.0	12.3	-0.1
Chr04_6088596	Chr04	6,088,596	21.6	3	-6.4	-1.0	-12.6	-0.3
Chr07_4630722	Chr07	4,630,722	44.5	3	-14.9	-0.7	-10.8	-0.1
Chr07_8572262	Chr07	8,572,262	39.9	3	12.4	0.7	11.3	0.1
Chr08_3090141_D	Chr08	3,090,141	8.8	3	-11.3	-1.9	-31.7	-2.0
Chr10_16255230_D	Chr10	16,255,230	38.2	3	-3.2	-0.8	-8.9	-0.1
Chr12_5301149	Chr12	5,301,149	28.8	3	1.6	0.3	5.6	-0.1
PR_71d	Chr14	5,849,948	21.0	3	-7.8	-0.5	-4.1	0.0
Chr16_1774410	Chr16	1,774,410	45.2	3	-13.1	-0.7	-8.8	-0.1
Chr17_14694510_D	Chr17	14,694,510	38.7	3	15.4	0.5	8.8	0.0
Chr22_3123319	Chr22	3,123,319	48.9	3	9.6	0.7	10.6	0.0

^1^ Minor allele frequency (%)

^2^ The number of analyzed environment × trait combinations with significant marker-trait associations

^3^ Mean effect of an alternative allele calculated for all tested environments. Traits are as follows: GDDs, the cumulative number of growing degree days; Days, the number of calendar days; Hours, the total number of photoperiod hours; VF, the cumulative vernalization effectiveness of daily temperature. All traits were counted from the sowing date until the start of flowering. In total, 6765 markers and 262 genotypes were used for GWAS.

**Fig. 5:**
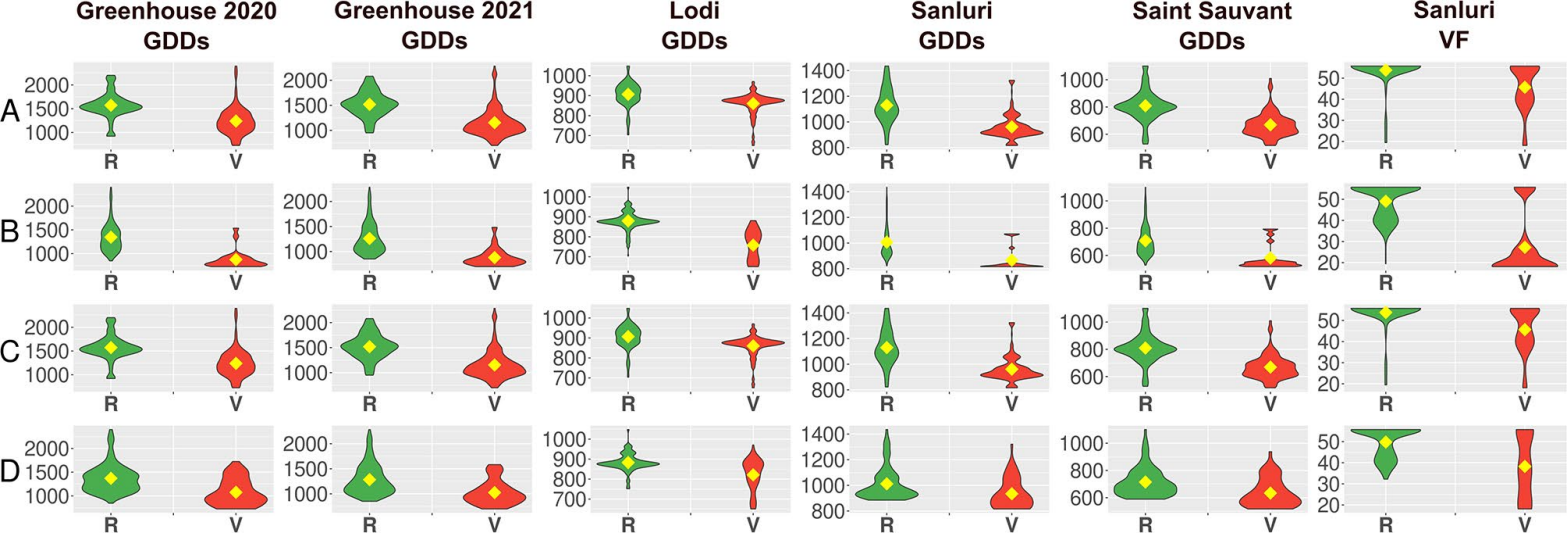
Allelic effects on the number of growing degree days (GDDs) and the cumulative vernalization effectiveness of daily temperature (VF) from sowing to flowering of white lupin for *LalbFTc1* gene PCR markers: PR_36b (A), PR_42a (B), PR_58c (C) and PR_71d (D). R stands for the reference allele (0), whereas V for an alternative allele (2) – see Table 3. Flowering time was observed in 4 environments: spring sowing in controlled conditions with absolute lack of vernalization (Greenhouse 2020 and 2021) as well as in field conditions - autumn sowing with strong (Lodi) or moderate vernalization (Sanluri), and spring sowing with mild vernalization (Saint Sauvant). Diamonds indicate mean values

Considering the number environment × trait combinations with significant associations, PR_36b and co-segregating PR_58c were among the most frequently associated markers in the whole dataset used for GWAS (together with the Chr25_4002891_D marker). PR_36b and PR_58c markers revealed high versatility across different agroclimatic conditions, highlighted by significant associations with GDDs and total photoperiod hours in all tested environments and with days to flowering in all environments except Lodi. The latter two *LalbFTc1* indel PCR markers revealed significant associations with days to flowering, GDDs, and total photoperiod hours in Greenhouse 2020 and Lodi (PR_42a) or only in Lodi (PR_71d). In a given scoring scheme (Table 3), alternative alleles (shorter products) were always selective for earliness (Fig. 5). It should be noted that these markers differed from each other by the frequency of alternative alleles, which ranged from 8.2% (PR_42a), through 21.0% (PR_71d) to 79.4% (PR_36b and PR_58c) (Table 4). Therefore, these four markers could constitute a ready-to-use mini array for future PCR-based selection towards early or late flowering in white lupin breeding.

To survey potential indel polymorphism in promoter regions of the remaining three *FT* homologs present in the white lupin genome, markers for *LalbFTa1*, *LalbFTa2*, and *LalbFTc2* genes were analyzed in a sub-population of 86 accessions representing observed diversity of flowering time in the studied diversity panel. From 43 primer pairs tested, 17 yielded polymorphic products: 5 for *LalbFTa1*, 9 for *LalbFTa2*, and 3 for *LalbFTc2* (Supplementary File S11). Seven markers (2 for *LalbFTa1* and 5 for *LalbFTa2*) had MAF values above 0.05. Polymorphic markers revealed remarkably less significant correlations with flowering time (r values between − 0.25 and 0.29) than four *LalbFTc1* markers (PR_36b, PR_42a, PR_58c, and PR_71d), highlighted by GWAS as significantly associated (r values between − 0.68 and − 0.45 in the same germplasm panel) (Supplementary File S12).

### GWAS confirmed the significance of three major flowering time QTL regions reported by linkage mapping studies

**Fig. 6:**
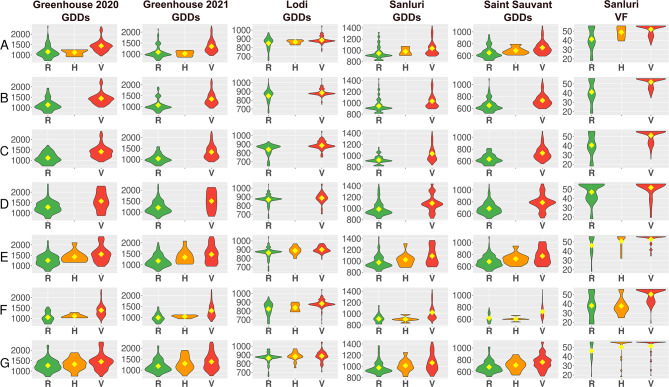
Allelic effects on the number of growing degree days (GDDs) and the cumulative vernalization effectiveness of daily temperature (VF) from sowing to flowering of white lupin for seven DArT-seq markers, Chr02_2625514_D (A), Chr02_2625564_D (B), Chr13_12561729_D (C), 13913452_D (D), Chr16_366800 (E), Chr16_572706 (F) and Chr16_788665 (G), originating from the chromosome segments overlapping with previously reported major QTL regions. R stands for the reference allele (0), V for an alternative allele (2), and H for a heterozygote (1). Flowering time was observed in four environments: spring sowing in controlled conditions with absolute lack of vernalization (Greenhouse 2020 and 2021) as well as in field conditions - autumn sowing with strong (Lodi) or moderate vernalization (Sanluri), and spring sowing with mild vernalization (Saint Sauvant). Diamonds indicate mean values

The 38 highly associated markers (Table 4) also encompassed seven DArT-seq markers originating from the chromosome segments overlapping with three major QTL regions identified by the linkage mapping studies [34, 35]. Namely, the Chr02_2625514_D and Chr02_2625564_D markers corresponding to a QTL localized on the linkage group ALB02 at 2.2 cM, the Chr13_12561729_D and Chr13_13913452_D markers matching a QTL from the linkage group ALB13 at 96.2 or 99.3 cM, and the Chr16_366800, Chr16_572706, and Chr16_788665 markers tagging a QTL from the linkage group ALB16 at 0.2 or 2.2 cM (Figs. 3 and 4). Alternative alleles of all these markers were selective towards late flowering, whereas heterozygotes revealed an intermediate phenotypic effect (Fig. 6). In both years, the highest number of environment × trait combinations with significant associations were revealed for the greenhouse. Nevertheless, two markers, Chr13_12561729_D and Chr16_366800, revealed significant associations with at least one trait in all studied environments, whereas the Chr16_572706 marker was particularly selective to Lodi and Chr02_2625514_D to Saint Sauvant. Markers Chr16_366800, Chr02_2625564_D and Chr13_12561729_D were the fourth, fifth, and sixth most frequently associated markers in this GWAS study (Fig. 3). On the other hand, PCR-based markers from the linkage map (Table 1), designed to track these QTLs, did not reveal significant associations with studied traits.

### GWAS highlighted novel candidate regulatory regions for flowering time in white lupin

**Fig. 7:**
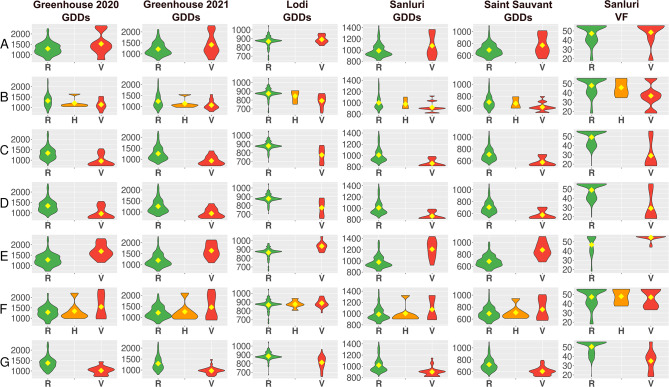
Allelic effects on the number of growing degree days (GDDs) and the cumulative vernalization effectiveness of daily temperature (VF) from sowing to flowering of white lupin for seven DArT-seq markers, Chr04_2175652 (A), Chr06_14434379 (B), Chr08_3090141_D (C), Chr08_3090075_D (D), Chr10_13080319 (E), Chr11_18778542 (F) and Chr25_4002891_D (G), tagging novel QTL regions. R stands for the reference allele (0), V for an alternative allele (2), whereas H for a heterozygote (1). Flowering time was observed in 4 environments: spring sowing in controlled conditions with absolute lack of vernalization (Greenhouse 2020 and 2021) as well as in field conditions - autumn sowing with strong (Lodi) or moderate vernalization (Sanluri), and spring sowing with mild vernalization (Saint Sauvant). Diamonds indicate mean values

The remaining 27 highly associated markers highlighted novel loci localized on 16 chromosomes (Table 4). The most remarkable in this subset was the Chr25_4002891_D marker, because it was, together with the PR_36b and PR_58c markers, the most frequently associated marker in this study, showing significant associations with at least two traits in every environment (Fig. 3, Supplementary File S10). Moreover, this marker was remarkably associated with vernalization progress (VF) in Sanluri (FDR-corrected P-value 8.7E-34), revealing a very strong negative effect of an alternative allele (-5.1) as compared to other markers (Fig. 3). Six other markers may also attract special attention, as they were very selective in particular environments: Chr04_2175652 and Chr11_18778542 in Greenhouse, Chr06_14434379 in Lodi, Chr08_3090141_D in Sanluri, Chr08_3090075_D and Chr10_13080319 in Saint Sauvant. The high selectivity of these markers in particular environments was reflected by extreme values of allelic effects. Markers Chr04_2175652, Chr10_13080319 and Chr11_18778542 revealed maximum values of an alternative allele effect for at least two environment × trait combinations in the whole dataset, whereas markers Chr06_14434379, Chr08_3090075_D, Chr08_3090141_D – the minimum values (Supplementary File S10, Fig. 7).

### Rapid linkage disequilibrium decay

**Fig. 8:**
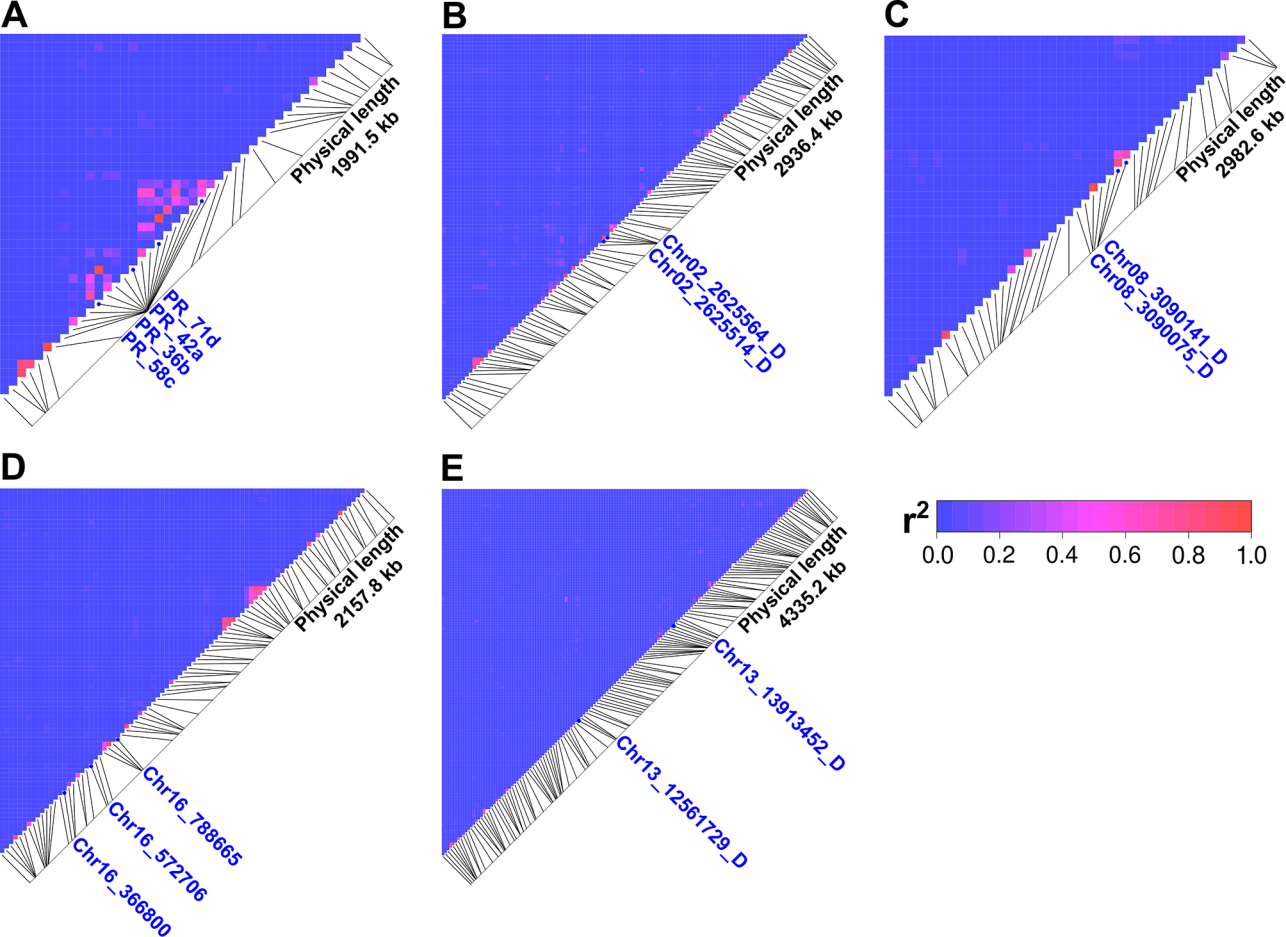
Linkage disequilibrium (LD) plots for white lupin genomic regions carrying significant markers localized in chromosomes Lalb_Chr14 (A), Lalb_Chr02 (B), Lalb_Chr08 (C), Lalb_Chr16 (D) and Lalb_Chr13 (E). The r^2^ values between significant SNPs are shown. Red indicates high measures of LD, while blue indicates low LD.

Five genome regions (around 2–4 Mbp) carrying significantly associated markers, localized in chromosomes Lalb_Chr02, Lalb_Chr08, Lalb_Chr13, Lalb_Chr14 (*Lalb_FTc1* gene) and Lalb_Chr16, were subjected to linkage disequilibrium (LD) analysis. This analysis revealed the presence of an LD block in the promoter region of the *Lalb_FTc1* gene, carrying, among others, PR_36b, PR_42a, PR_58c and PR_71d PCR indel markers (Fig. 8A). Nevertheless, associations quickly diminished with distance between sites in distal part of the promoter and in the coding sequence. For regions localized in chromosomes Lalb_Chr02, Lalb_Chr08 and Lalb_Chr16 only very small LD blocks carrying 3 loci were identified in the proximity of markers highlighted by GWAS as significant (Fig. 8B-D), whereas for the region in chromosome Lalb_Chr13 no LD block was found (Fig. 8E).

## Discussion

### Germplasm resources for improvement of white lupin adaptation towards higher latitudes

The population structure analysis revealed a germplasm separation originating from geographically isolated regions or belonging to different phenological types (spring- or winter). This finding agrees with the results of previous phylogenetic studies that grouped white lupin accessions into several clades, highlighting the high diversity of *graecus*-type accessions and the genetic distinctiveness of some Ethiopian landraces that putatively evolved in high isolation [39, 49, 50]. In general, the grouping pattern in our study agreed with the existing variability in flowering time and vernalization responsiveness. We could identify at least four different clusters providing sources of early flowering germplasm for spring-sowing breeding, including one group carrying several rapid flowering genotypes from Turkey, Syria and Jordan that outperformed current French spring-type cultivars by 10–20 days. This finding has high perspective interest in white lupin adaptation to the expected global northward shift of agricultural climate zones [30] and the current emphasis on breeding of legume plants adapted to spring sowing in high latitudes [51]. As reported previously, autumn-sown genotypes with strong vernalization requirement (winter type) formed a separate clade [39]. The difference in mean flowering time between the two most extreme clades was remarkable, reaching 67 days for Sanluri (autumn sowing with moderate vernalization) and 61 days for the greenhouse experiment (spring sowing without vernalization). Plants in the greenhouse were cultivated under temperatures not lower than 18 °C, i.e. at least several degrees above the vernalization threshold which is about 12 °C [20]. In contrast, in Lodi the mean daily temperature from October to February lowered to 5.7 °C, resulting in effective vernalization for about four months (VF value of 121.97 days). Vernalization conditions in Sanluri and Saint Sauvant were only partially fulfilled, due to higher mean temperature in the juvenile growth phase (12.6 °C and 10.7 °C, respectively) and a lower number of days with vernalization-enabling conditions, resulting in VF values of 55.73 and 18.67 days, respectively. The numbers of GDDs from sowing to flowering observed in Lodi and Sanluri matched the range reported for white lupin [18, 22] whereas in Saint Sauvant and both greenhouse experiments were significantly higher, especially for late flowering genotypes. It highlighted the great vernalization requirements of those accessions. Four significantly associated SNPs identified in our study (Chr01_5255422, Chr01_5921843, Chr13_1140246 and Chr13_1469866) originate from similar genome regions as two of seven SNPs significantly associated with days to flowering during drought tolerance experiment in Lodi [28]. Interestingly, plants in the reported drought study were sown in mid-February, allowing partial vernalization, whereas one of those SNPs, Chr13_1140246, in our experiment, was significantly associated with cumulative vernalization effectiveness of daily temperature until flowering in Lodi. Both observations emphasize the vernalization-responsive component of the white lupin flowering regulatory network.

On the other hand, numerous genotypes with intermediate phenology revealed in this study suggests a complex genetic architecture of flowering time control in white lupin. This situation differs from that described for narrow-leafed lupin, where the number of intermediate genotypes is very low, and just a few key mutations controlling flowering time were identified hitherto [12–14, 52]. The low number of germplasm accessions with early flowering that were available for narrow-leafed lupin breeding in the mid-1960s (just two) caused a significant genetic bottleneck that resulted in a large loss of genetic diversity in the domesticated germplasm [53–55]. The identification of untapped germplasm resources with early phenology from different phylogenetic clades could prevent such as genetic erosion in white lupin breeding. The third Old World cultivated lupin species, *Lupinus luteus*, revealed a genetic architecture of flowering time similar to white lupin, with a continuous trait distribution and several mutations associated with extreme phenology (early or late) identified and supplemented with PCR markers for molecular selection [11]. The present study provided four PCR indel markers enabling selection towards earliness or vernalization responsiveness, along with several candidate SNP markers that await further implementation.

### Indel polymorphism in *FT* regulatory regions and environmental adaptation

Our study revealed a significant association between flowering time and indel polymorphism in three regions in the *LalbFTc1* promoter, the first located from − 7996 bp to -5609 bp in relation to the TSS, the second from − 4408 bp to -4145 bp, and the third from − 780 bp to -753 bp. *LalbFTc1* in one of the four *FT* homologs present in the white lupin genome [11]. Research on the model plant, *Arabidopsis thaliana*, evidenced that *FT* is a key component of the flowering regulatory network that integrates signals from various pathways, including those related to ambient temperature, vernalization and photoperiod [56–58]. The *A. thaliana* genome encodes only two *FT*-like genes, *FT* and a one close homolog, *TWIN SISTER OF FT* (*TSF*), that are partially sub-functionalized into photoperiod signaling (long vs. short day) and ambient temperature response [59, 60]. Regulation of *FT* expression is performed at two major levels: epigenetic (mainly by histone modifications) and genetic (by transcription factor binding) [61–64]. Regulatory regions of *FT* in *A. thaliana* include an enhancer located about 5.5 kilobases (kb) upstream of the transcriptional start site (TSS), a relatively long promoter sequence (~ 5 kb) with a few conserved blocks, three *FT* gene introns, and an enhancer located 1 kb downstream of the gene [57, 62, 64–66]. A physical convergence of cis-regulatory elements from all major environment-responsive biological pathways controlling flowering induction at the *FT* gene [57] opened up opportunities for advancement in environmental adaptation during evolution or domestication and breeding by fixation of occasional sequence polymorphism. For example, the major QTL for flowering time in a *A. thaliana* intercross-recombinant inbred line population was located 6.7-kb upstream of the *FT* coding sequence, in a region carrying two large overlapping indels and several other polymorphisms [67]. Structural variants of *FT* promoter (a deletion of ~ 250 bp and an insertion of ~ 1.1 kb) were also found in natural *A. thaliana* populations in Eurasia and were associated with geographic distribution into lower and higher latitudes, respectively [68].

Legume genomes typically contain more *FT*-like genes than the genome of *A. thaliana*, which were putatively preserved after ancestral and lineage-specific whole-genome duplication (WGD) events [59, 69, 70]. These genes were assigned into three subclades: *FTa*, *FTb* and *FTc* [71]. In general, gene duplication can be considered as a mechanism of genomic adaptation to a changing environment [72]. Thereby, selection pressures might be relaxed, providing some opportunity for independent evolution and functional novelty [73]. The occurrence of an ancestral legume WGD event highly converges with the Cretaceous–Paleogene (K–Pg) mass extinction event [74, 75]. Evolution of vernalization responsiveness in temperate plants could be facilitated both by rapid short-term cooling during K–Pg extinction and consecutive global cooling during Eocene–Oligocene transition [76–78]. Dating of major climatic events provides a possible explanation of the reason why vernalization pathways differ between plant families: they do, because plant families were already separated at the time of those climate changes [57].

Among legumes, the molecular control of flowering has mainly been studied in soybean, as this species is natively a short day plant, whereas many current cultivation areas are located under long day photoperiod. More than a dozen early maturity loci have been identified in soybean hitherto. Corresponding genes were identified for most of them, including three *FLOWERING LOCUS T* (*FT*) homologs for the loci *E9*, *E10* and *qDTF-J1*, namely *GmFT2a*, *GmFT4* and *GmFT5a*, that differ for functional mutations and provide adaptation to low and high latitudes, respectively [79–81]. Moreover, the geographic distribution of sequence polymorphism at soybean *GmFT2a* and *GmFT5a* genes revealed strong latitudinal gradient, highlighting ongoing selection for long juvenile alleles in the tropics [82]. In white lupin such an adaptive mutation for low latitudes could be attributed to a 683 bp deletion in the third intron of *LalbFTa1* gene, associated with delayed flowering of some Ethiopian landraces [34–36].

### *FTc1* promoter indels confer vernalization independence in domesticated Old World lupin species

Vernalization independence in *Medicago truncatula spring* mutants was found to be associated with retroelement insertions at *MtFTa1*, causing overexpression of this gene [83]. In chickpea, a major QTL for vernalization response explaining the majority of phenotypic variance was localized at LG3, overlapping with the main domestication locus controlling growth habit and early flowering under short days [84, 85]. Phenotypic effects were associated with a high expression of the *FT* gene cluster (*FTa1*, *FTa2*, and *FTc*), hypothetically driven by unknown cis-acting genetic change [84]. In lentil (*Lens culinaris*), vernalization independence was reportedly linked with de-repression of the *LcFTa1* gene, putatively due to identified sequence polymorphism at *LcFTa1–LcFTa2* cluster: a large 7441 bp deletion in the *LcFTa1–LcFTa2* intergenic region and a 245 bp deletion located in the *LcFTa1* promoter, 3712 bp upstream of the start codon [86]. Interestingly, the coordinates of the latter indel match three overlapping *LanFTc1* promoter indel variants found in narrow-leafed lupin and the *LLutFTc1* promoter indel found in yellow lupin, all of them conferring overexpression of promoted genes and vernalization independence in those species [11–14]. Moreover, indel coordinates in these lupin species overlap with a 264 bp deletion in the middle region (-4145 bp) of *LalbFTc1* promoter discovered in the present study (marker PR_36b). The direction of effects (early flowering of shorter alleles) was the same for a 28 bp deletion (markers PR_42a and PR_71d) associated with rapid flowering of Turkish, Syrian and Jordanian genotypes (located 780 bp upstream of the *LalbFTc1* start codon) and large *FTc1* promoter indels in lentil and two lupin species studied hitherto [11–14, 86]. Rapid LD decay was revealed around the promoter of the *LalbFTc1* gene in the white lupin diversity panel. The same phenomenon was observed around the *LanFTc1* gene in the narrow-leafed lupin germplasm collection carrying domesticated and wild accessions [13].

## Other possible components of flowering time regulatory network in white lupin

Genomic positions of selected DArT-seq markers that revealed significant marker-trait associations (Figs. 6 and 7) were referred to gene annotations reported in the white lupin genome portal [38, 39] (https://www.whitelupin.fr). Six markers were found in exons, three in introns, four in untranslated regions (UTRs) and only one in a region lacking any annotations (Table 5). Taking into consideration expected protein sequences, an alternative allele of Chr06_14434379 marker conferred synonymous mutation, whereas alternative alleles of Chr11_18778542, Chr16_366800 and Chr16_788665 markers – nonsynonymous mutations, namely Ser to Arg, Asn to Asp and Glu to Gly. Interestingly, three gene-based DArT-seq markers were also localized in miRNA clusters. These clusters revealed a remarkably high number of hits to sequences deposited in RNAcentral miRbase [87], namely Cluster_43033 for a presence/absence SilicoDArT marker Chr08_3090075_D − 419, including 362 long non-coding RNAs (lncRNAs), with the best hit e-value of 2.4e-131 (BNAP_LNC001257 from *Brassica napus*), Cluster_70755 for a presence/absence SilicoDArT marker Chr13_13913452_D − 752, including 646 lncRNAs, with the best hit e-value of 4.3e-153 (PVUL_LNC004086 from *Phaseolus vulgaris*) and Cluster_127516 for a presence/absence SilicoDArT marker Chr25_4002891_D – 955, including 947 lncRNAs, with the best hit e-value of ~ 0.0 (LANG_LNC003687 from *Lupinus angustifolius*).

Long non-coding RNAs control developmental processes such as flowering via vernalization and autonomous pathways, seed germination, light- and auxin-regulated development, and are also involved in plant responses to abiotic and biotic stress [88–90]. Moreover, long non-coding RNAs drive RNA-dependent DNA methylation to generally suppress transposons in the genome as well as to repress the transcription of specific protein-coding genes, including *FLOWERING WAGENINGEN* (*FWA*) conferring a late flowering *A. thaliana* phenotype [88, 91]. Here, a long non-coding RNA Cluster_43033 was identified in an ATPase gene *Lalb_Chr08g0233891*. There is little information on the contribution of ATPase genes into flowering pathways, except for the *FLOWERING REPRESSOR AAA + ATPase 1* (*AaFRAT1*) that regulates perennial flowering in a vernalization-dependent manner in *Arabis alpine*, a mountainous plant from the Brassicaceae family [92]. Taking into consideration that SilicoDArT markers may represent methylation variation at restriction sites, the absence of an allele of the Chr08_3090075_D marker in early flowering accessions from Turkey, Syria and Jordan agrees with the hypothesis of an involvement of RNA-dependent DNA methylation mediated by long non-coding RNAs. Nevertheless, to draw any further conclusions, validation by transcriptomic and epigenetic profiling of germplasm carrying opposite allele phases in contrasting environmental conditions should be performed.

**Table 5:** Genetic annotation of selected DArT-seq markers, highlighted by GWAS as significantly associated

Marker	Gene	Region	Annotation	miRNA cluster	miRBase
Chr02_2625514_D	Lalb_Chr02g0145521	3’UTR	hypothetical protein	-	-
Chr02_2625564_D	Lalb_Chr02g0145521	3’UTR	hypothetical protein	-	-
Chr04_2175652	Lalb_Chr04g0250871	3’UTR	hypothetical protein	-	-
Chr06_14434379	Lalb_Chr06g0173521	2nd exon	putative EF-hand binding protein	-	-
Chr08_3090075_D	Lalb_Chr08g0233891	10th exon	putative proton-exporting ATPase	Cluster_43033	+
Chr08_3090141_D	Lalb_Chr08g0233891	10th exon	putative proton-exporting ATPase	Cluster_43033	+
Chr10_13080319	Lalb_Chr10g0097551	19th intron	putative exoribonuclease II	-	-
Chr11_18778542	Lalb_Chr11g0074371	1st exon	putative LysM, EEIG1/EHBP1 domain-containing protein	-	-
Chr13_12561729_D	-	-	-	-	-
Chr13_13913452_D	Lalb_Chr13g0300751	8th intron	Putative initiation factor eIF-4 gamma, MA3	Cluster_70755	+
Chr16_366800	Lalb_Chr16g0376931	2nd exon	putative UDP-glucose 4-epimerase	-	-
Chr16_572706	Lalb_Chr16g0377361	4th intron	putative DNA-directed RNA polymerase	-	-
Chr16_788665	Lalb_Chr16g0377681	9th exon	hypothetical protein	-	-
Chr25_4002891_D	Lalb_Chr25g0281301	3’UTR	hypothetical protein	Cluster_127516	+

## Conclusions

Our study revealed the substantial agreement of population structure with germplasm differences in phenology and geographical origin, and highlighted spring-type germplasm for white lupin breeding targeting high latitudes. We identified a set of markers from PCR-based and DArT-seq datasets that were significantly associated with flowering time in a range of environments and tagged three indels from the *LalbFTc1* promoter and several other loci localized in different white lupin chromosomes. Our results supported the convergence of *FTc1* promoter indel evolution into vernalization pathway in Old World lupin species, and provided a set of molecular markers for tracking of such indels in white lupin breeding programs. The substantially polygenic control of white lupin flowering time supports the development of genomic selection models exploiting DArT-seq genotyping technology and four *LalbFTc1* promoter PCR markers.

### Methods

#### White lupin germplasm diversity panel

The plant material encompassed 262 genotypes randomly selected from 120 accessions representing 11 landrace and 2 cultivar pools [10]. Landraces originated from seven national pools (Azores, Egypt, Greece, Italy, Portugal, Spain, and Turkey) and four transnational pools (East Africa, Madeira & Canaries, Maghreb, and West Asia), each represented by at least eight accessions. Cultivars represented spring-type and winter-type crops included in the French Register of Varieties, each represented by four entries. Collection sites represented diverse climatic conditions such as tropical and subtropical highlands (Ethiopia), cold semi-arid (i.e. some locations in Anatolia and Maghreb), dry-summer subtropical (i.e. hot-summer Mediterranean: the majority of collection sites around Mediterranean Basin), humid subtropical (i.e. warm-summer Mediterranean: Azores), humid temperate (i.e. oceanic: French cultivars). These regions also diverged by photoperiod during the juvenile phase of white lupin growth, ranging from about 9–10 h in winter sowing in northern regions of the Mediterranean Basin to 11–12 h in Ethiopia and 12–14 h in spring sowing in France. The list of accessions is provided in Supplementary File S13. World landrace collection held by Council for Agricultural Research and Economics (CREA) is based one the majority of landrace accessions that were received from INRA Lusignan (now INRAE) in 2002, of which INRA’s accession name is reported in the last column of Supplementary File S13. The material was provided within the framework of the long-standing scientific collaboration between INRA Lusignan and ISCF Lodi (now CREA-ZA Lodi). From each of these accessions, CREA extracted, genotyped, multiplied and characterized up to 4 individual genotypes. CREA’s world landrace collection has been characterized by CREA and Jouffray-Drillaud (currently Cérience).

### Phenotyping of flowering time

Field observations were performed during the 2004–2005 growing season as a part of a landrace evaluation study [10] performed under autumn sowing in Lodi (Lombardy, Italy, 45°19’ N, 9°03’ E; representing a temperate subcontinental climate) and Sanluri (Sardinia, Italy, 39°30’ N, 8°50’ E; representative of hot-summer Mediterranean climate) as well as under spring sowing in Saint Sauvant (western France, 46°22’ N 0°5’ E; representative of an oceanic climate). Sowing dates were 14th October in Lodi, 29th October in Sanluri, and 9th March in Saint Sauvant. Data on minimum and maximum air temperature, cumulative growing degree days (GDDs), cumulative vernalization effectiveness of daily temperature (VF) and total photoperiod hours recorded in Lodi, Sanluri, Saint Sauvant and Greenhouse during the course of experiments are provided in Supplementary File S14. Detailed conditions of plot sizes, randomization and plant cultivation were provided in the paper reporting the results of landrace evaluation [10]. Days to onset of flowering from sowing were counted when 50% of plants for a given plot developed first fully colored petals.

Greenhouse observations were performed by spring sowing (19th March 2020 and 11th March 2021) at the Institute of Plant Genetics, Polish Academy of Sciences, Poznań, Poland (52°26′ N 16°54′ E) in Poznań (western Poland, representative of temperate subcontinental climate) under ambient long-day photoperiod, increasing from about 12 to 16 h during plant cultivation. Automatic heating was used to keep the minimum air temperature above 18 °C, whereas cooling was maintained by temperature-dependent window-opening system (activated at 22 °C). Flowering time was recorded as the number of days from sowing to the observation of the first fully colored petal. Observations were made in three biological replicates.

Cumulative growing degree days (GDDs) were calculated using the formula:

$$\:GDDs={\sum\:}_{t=1}^{n}\text{m}\text{a}\text{x}(Td-Tb;0)$$

Where *t* and *n* are days from sowing and the total number of days from sowing to the start of flowering, *Td* is a daily mean temperature, whereas *Tb* corresponds to the base temperature of white lupin parameterized in this study as 3 °C [18, 22]. GDD values for fractional days were calculated on a linear scale.

Daily mean temperature was calculated using the formula:

$$\:Td=\frac{Tmax+Tmin}{2}$$

where *Tmax* and *Tmin* are daily maximum and minimum temperatures.

Cumulative vernalization effectiveness of daily temperature (VF) was calculated using the formula proposed by Wu et al. 2017 [93]:

$$\:VF=\sum\:_{t=1}^{n}\text{m}\text{a}\text{x}(1-{\left[\frac{Topt-Td}{Tamp}\right]}^{2};0)$$

where *t* and *n* are days from sowing and the total number of days from sowing to the start of flowering, *Td*, *Topt*, and *Tamp* are the mean daily temperature, optimal vernalization temperature and thermal semi-amplitude of vernalizing effect, respectively. *Topt* and *Tamp* for white lupin were parameterized in this study as 3 °C and 9 °C corresponding to the range of vernalization-effective temperature between − 6 °C and + 12 °C. VF value of 1 corresponds to 1 day (24 h) of optimal vernalization conditions.

### DNA isolation

Two young upper leaves (about 50–100 mg tissue per collection tube) were collected from 5 week-old plants (a single representative per genotype cultivated in the greenhouse) and immediately frozen under liquid nitrogen. Homogenization of frozen plant tissue was performed using TissueLyser II (Qiagen, Hilden, Germany) and two stainless steel beads (ø 5 mm, Qiagen) placed in a 2 mL tube (Eppendorf, Hamburg, Germany) for 45 s at 30 rpm. DNA isolation was performed with Maxwell^®^ RSC PureFood GMO and Authentication Kit (Promega, Mannheim, Germany) harnessing automated station Maxwell^®^ RSC 48 Instrument (Promega) with standard protocol, yielding on average 100 ± 29 µg DNA per sample. DNA concentration and quality were estimated using a NanoDrop 2000 (ThermoFisher Scientific, Warsaw, Poland).

### DArT-seq genotyping

DNA isolates were subjected to Lupin DArTseq (1.0) protocol, with 2.5 mln reads sequencing depth (Illumina Novaseq6000). DArT-seq protocol and genotype calling were performed by Diversity Arrays Technology Pty Ltd. (University of Canberra, Bruce, Australia). DArTseq generated two types of data: scores for presence/absence (dominant) markers (SilicoDArTs) and standard single nucleotide polymorphism (SNP) markers. SilicoDArTs represent several possible polymorphisms: SNPs and small indels in restriction enzyme recognition sites, larger insertions/deletions in restriction fragments and methylation variation at restriction sites. The reference allele corresponds to the allele present in white lupin genome assembly GCA_009771035.1.

### PCR-based genotyping of indel polymorphism in the *FT* gene promoters

As in the two other Old World lupin crop species (i.e., narrow-leafed lupin and yellow lupin) indel polymorphism in the regulatory region of *FTc1* homologs (*LanFTc1* and *LlutFTc1*, respectively) revealed association with time to flowering and vernalization responsiveness [11–14], we decided to supplement DArT-seq dataset with PCR markers targeting structural variation in the promoter region of white lupin *FTc1* homolog, *LalbFTc1*. DNA sequence carrying the *LalbFTc1* gene (*Lalb_Chr14g0364281*) [11] with 8100 bp of 5’ regulatory region (full promoter with proximal and distal CCAAT boxes) was extracted from the white lupin genome assembly [38]. The set of primer pairs (Table 3) was designed to amplify overlapping PCR products spanning the region from the 8065 bp upstream of the transcription start site to the first exon of the *LalbFTc1* gene. Moreover, additional PCR primers were designed, targeting particular indels identified in the multiple sequence alignment of white lupin pangenome carrying 40 accessions [39]. The same procedure was performed for the remaining white lupin *FT* homologs, *LalbFTa1* (*Lalb_Chr02g0156991*), *LalbFTa2* (*Lalb_Chr21g0317021*) and *LalbFTc2* (*Lalb_Chr09g0331851*).

Primers were designed using Primer 3 Plus [94] implemented in Geneious Prime [95], whereas sequence alignment was performed using the progressive Mauve algorithm [106] assuming genome collinearity. Primer sequences are provided in Supplementary File S15. PCR products up to 2 kb in length were amplified using GoTaq G2 Flexi DNA Polymerase (Promega) whereas longer products used GoTaq^®^ Long PCR Master Mix (Promega). Length differences were visualized by agarose gel electrophoresis with the agarose concentration (1–3%) adjusted to follow the size of the expected products. Small indels (shorter than about 50 bp) were resolved using high resolution 3:1 agarose (Serva, Heidelberg, Germany) whereas larger ones with wide range agarose (Serva). Selected polymorphic amplicons were directly Sanger-sequenced using BigDye^®^ Terminator v3.1 Cycle Sequencing Kit (Applied Biosystems) and 96-capillary 3730xl DNA Analyzer (Applied Biosystems) by Genomed (Warsaw, Poland). *LalbFTc1* markers were analyzed in the whole panel (262 accessions), whereas *LalbFTa1*, *LalbFTa2* and *LalbFTc2* markers in a sub-population of 86 accessions representing contrasting values of flowering time in studied environments and allelic composition of significantly associated markers.

### PCR-based genotyping of flowering time QTLs from linkage map

Besides DArT-seq and *LalbFTc1* indel markers, the white lupin germplasm panel was subjected to genotyping with the set of fifteen PCR-based markers (Table 1) tagging four major QTLs for flowering time from linkage mapping studies [34, 35] and several candidate genes that in germplasm collection revealed significant correlation between sequence polymorphism and time to flowering [36]. Depending on the availability of restriction enzymes, SNPs were resolved by the cleaved amplified polymorphic sequence (CAPS) [46] or derived CAPS (dCAPS) [47] approaches. Full-length original gel images for cropped gels displayed in Supplementary Files S6 and S7 were provided in Supplementary File SS16.

### Calculation of heritability

Heritability in a broad sense was calculated using the formula:

$$\:{H}^{2}=\frac{{\sigma\:}_{G}^{2}}{{\sigma\:}_{G}^{2}+\raisebox{1ex}{${\sigma\:}_{E}^{2}$}\!\left/\:\!\raisebox{-1ex}{$r$}\right.}$$

$$\:{\sigma\:}_{G}^{2}={(MS}_{G}-{MS}_{E})/r\:\text{a}\text{n}\text{d}\:{\sigma\:}_{E}^{2}={MS}_{E},$$

Where:

$$\:{\sigma\:}_{G}^{2}-genotype\:variance$$

$$\:{\sigma\:}_{E}^{2}-residual\:variance$$

$$\:{MS}_{G}-Mean\:square\:of\:genotype$$

$$\:{MS}_{E}-Mean\:square\:of\:residual$$

$$\:r\:-replication$$

### Imputation of genotypes and population structure analysis

All marker data were transformed to 0, 1, 2 code, where 0 stands for the homozygote, 2 for the alternative allele homozygote, and 1 for the heterozygote. For data transformation, custom python script were used. For missing data imputation was performed using beagle software version 4.1 [96] with its default settings. Duplicated loci with identical segregation were removed leaving a single representative. That prepared data was filtered for minor allele frequency (MAF) with threshold of 0.05. Q matrix with population structure for the estimation of ancestral populations (K) in range 2 to 30 was calculated using ‘snmf’ function from the LAE package [97]. The analysis was done for each K-value, using 3000 replications and 5000 iterations. The best run was obtained using cross-entropy criterion. Based on the obtained results for further analysis, K-values in the range of 4–13, 15 and 17 were used. In parallel, principal component analysis (PCA) analysis of 6 765 SNP markers and 262 genotypes was conducted using the prcomp function from R Software.

### Genome-wide association mapping and visualization

GWAS was performed using the Bayesian-information and Linkage-disequilibrium Iteratively Nested Keyway (BLINK) model [98] implemented in the GAPIT R package [99]. In the analysis, we accounted for population structure (Q) through a Qmatrix and for relationships among individuals through a kinship (K) matrix [100], both using the marker data. For each trait, Qmatrix of covariates with K = 5 was used. The significance threshold for marker-trait associations (MTA) was set to *p* = 0.05 after applying the false discovery rate (FDR) [101] correction. Visualization of population structure, PCA map and violin plots was made in R software using packages ggplot2 [102], GAPIT [99] factoextra [103] and pophelper [104]. LD graphs were prepared using LDheatmap [105], whereas Manhattan plots in qqman package (https://cran.r-project.org/web/packages/qqman/index.html).

### Electronic supplementary material

Below is the link to the electronic supplementary material.

Supplementary Material 1: Supplementary_File_S1.xlsx: Phenotypic observations recorded in studied environments for white lupin germplasm diversity panel.

Supplementary Material 2: Supplementary_File_S2.xlsx: Total photoperiod (day light) hours from sowing to start of flowering recorded in studied environments for white lupin germplasm diversity panel.

Supplementary Material 3: Supplementary_File_S3.xlsx: Cumulative number of growing degree days from sowing to start of flowering (GDDs) recorded in studied environments for white lupin germplasm diversity panel.

Supplementary Material 4: Supplementary_File_S4.xlsx: Cumulative vernalization effectiveness of daily temperature (VF) from sowing to start of flowering recorded in studied environments for white lupin germplasm diversity panel.

Supplementary Material 5: Supplementary_File_S5.xlsx: DarT-seq and PCR-based markers used for population structure analysis and genome-wide association study in white lupin germplasm diversity panel.

Supplementary Material 6: Supplementary_File_S6.pdf: Agarose gel electrophoregrams showing polymorphism of PCR-based markers tagging white lupin flowering time quantitative trait loci (QTLs) from linkage mapping studies.

Supplementary Material 7: Supplementary_File_S7.pdf: Agarose gel electrophoregrams showing polymorphism of PCR-based markers developed for white lupin LalbFTc1 gene promoter indels.

Supplementary Material 8: Supplementary_File_S8.pdf: Values of the cross-entropy criterion for a number clusters ranging from K1 to K30.

Supplementary Material 9: Supplementary_File_S9.xlsx: Results of population structure analysis in white lupin germplasm diversity panel.

Supplementary Material 10: Supplementary_File_S10.xlsx: FDR-corrected P-values and phenotypic effects of markers analyzed in genome-wide association study of white lupin germplasm diversity panel for flowering time in controlled environment and field conditions.

Supplementary Material 11: Supplementary_File_S11.xlsx: Results of PCR-based screening of indel polymorphism in promoter regions of LalbFTa1, LalbFTa2, LalbFTc1 and LalbFTc2 genes in white lupin germplasm diversity panel.

Supplementary Material 12: Supplementary_File_S12.xlsx: Correlations between studied traits and PCR-based markers tagging indel polymorphism in promoter regions of LalbFTa1, LalbFTa2, LalbFTc1 and LalbFTc2 genes in white lupin germplasm diversity panel.

Supplementary Material 13: Supplementary_File_S13.xlsx: The list of genotypes from white lupin germplasm diversity panel analyzed in the study.

Supplementary Material 14: Supplementary_File_S14.xlsx: Minimum and maximum air temperature, cumulative growing degree days (GDDs), cumulative vernalization effectiveness of daily temperature (VF) and total photoperiod hours recorded in Lodi, Sanluri, Saint Sauvant and Greenhouse during the course of experiments.

Supplementary Material 15: Supplementary_File_S15.xlsx: Primer sequences of PCR-based markers developed for white lupin LalbFTa1, LalbFTa2, LalbFTc1 and LalbFTc2 gene promoter indels and QTL screening.

Supplementary Material 16: Supplementary_File_S16.pdf: Full-length original gel images for cropped gels displayed in Supplementary Files.

Supplementary Material 17: The list of supplementary files.

## Data Availability

CREA’s world landrace collection has been characterized by CREA in Lodi and evaluated by CREA and Jouffray-Drillaud (currently Cérience), specifically by two co-authors of this work (PA and NH). No voucher specimen has been deposited anywhere by CREA. However, the original landraces of INRA Lusignan’s world collection (from which individual genotypes were extracted, multiplied and genotyped) have become part of INRAE’s germplasm collection in Dijon and, as such, are available according to INRAE’s regulations for germplasm exchange. All data generated or analyzed during this study are included in this published article and its supplementary information files. The sequence variant data have been deposited in the European Variation Archive (EVA) at European Molecular Biology Laboratory - European Bioinformatics Institute (EMBL-EBI) as a study “Lupinus albus genome-wide association study for phenology traits” under project PRJNA939025, analysis accession number ERZ16297462.
