# Supplementary material for: A GWAS study highlights significant associations between a series of indels in a FLOWERING LOCUS T gene promoter and flowering time in white lupin (Lupinus albus L.)
Source: BMC Plant Biol. 2024 Jul 29;24:722. doi: 10.1186/s12870-024-05438-1 (PMC11285409; doi:10.1186/s12870-024-05438-1)

Sandra Rychel-Bielska, Wojciech Bielski, Anna Surma, Paolo Annicchiarico, Jolanta Belter, Bartosz Kozak, Renata Galek, Nathalie Harzic, Michał Książkiewicz

A GWAS study highlights significant associations between a series of indels in a *FLOWERING LOCUS T* gene promoter and flowering time in white lupin (*Lupinus albus* L.)

BMC Plant Biology

Supplementary File S6. Agarose gel electrophoregrams showing polymorphism of PCR-based markers tagging white lupin flowering time quantitative trait loci (QTLs) from linkage mapping studies.

K –Kiev Mutant; D – P27174; 0 – “reference” allele; 1 – heterozygote; 2 – “variant” allele

QTL1_MFT-FT3-F1

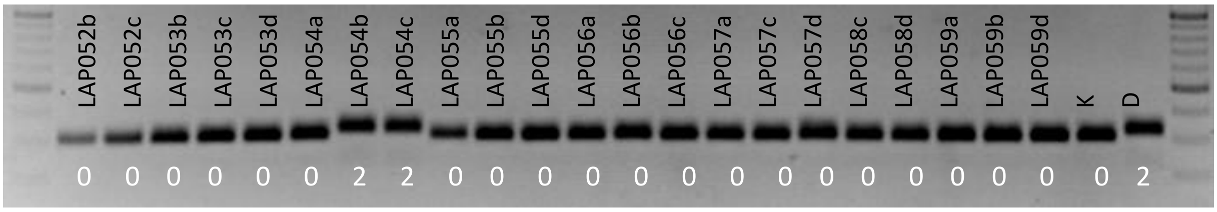

QTL2_FTc1-F4

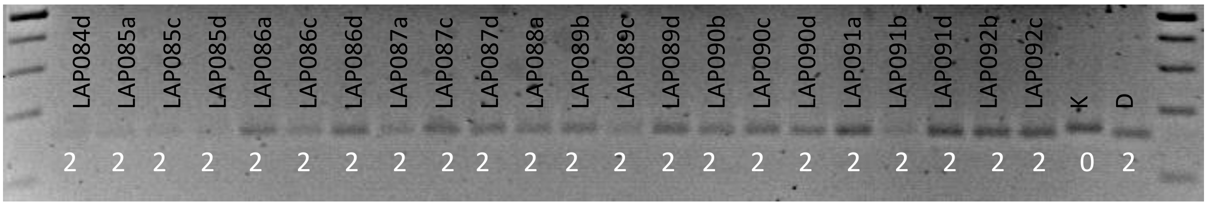

QTL3_FY-F6

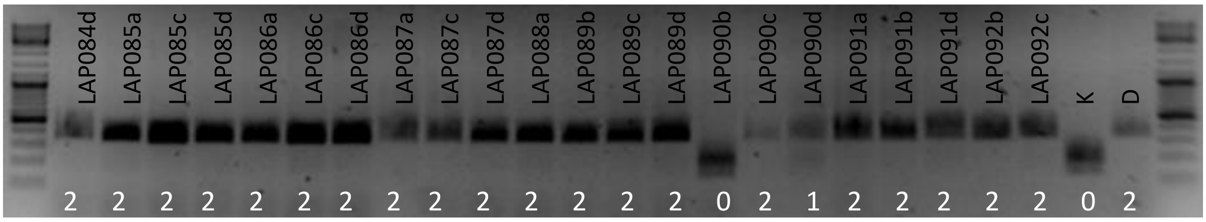

QTL5_VIP3-F2

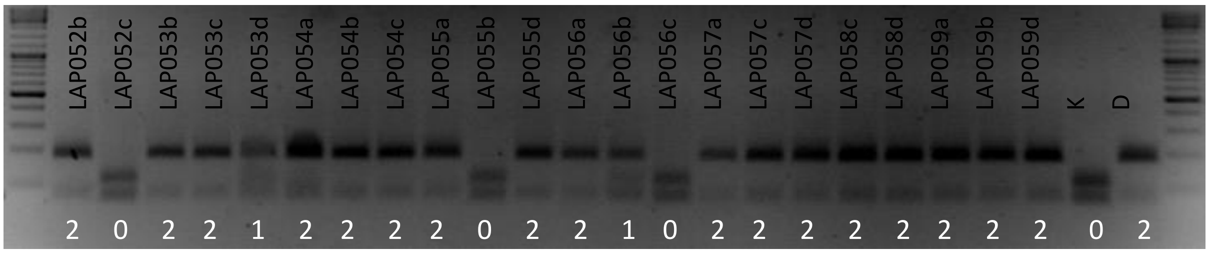

QTL6_TP2390

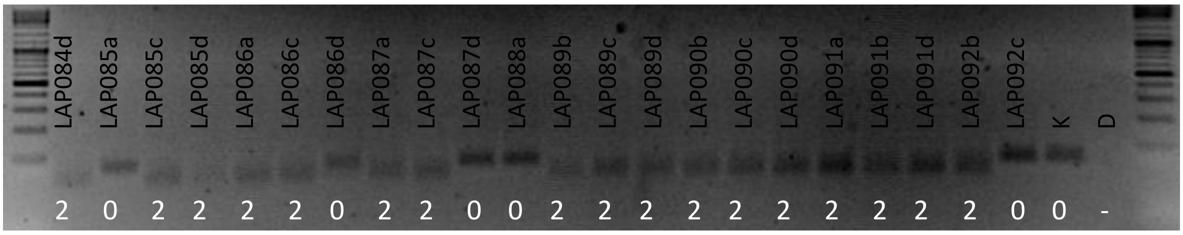

QTL7_TP235608

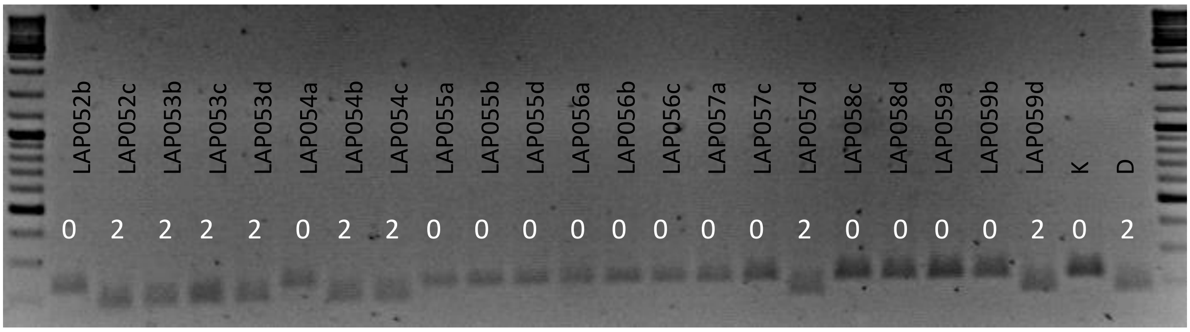

QTL8_TP94353

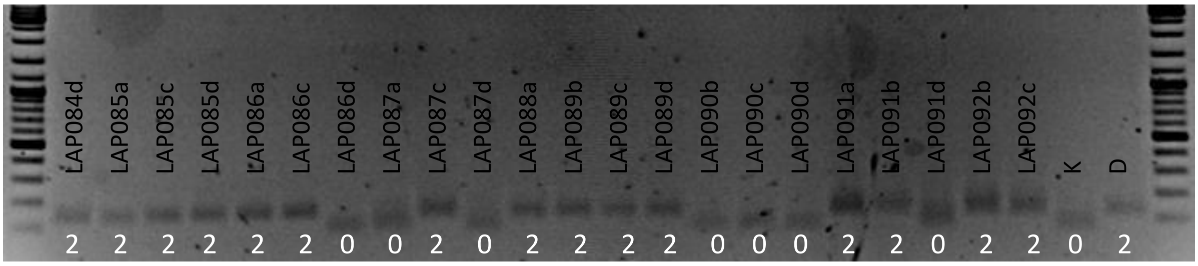

QTL9_SKIP1-F2

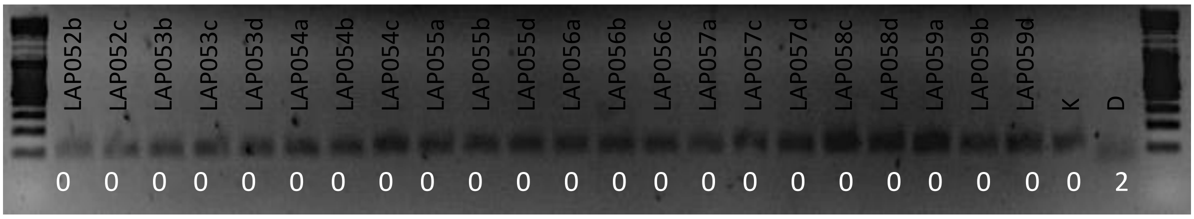

QTL10_TP402859

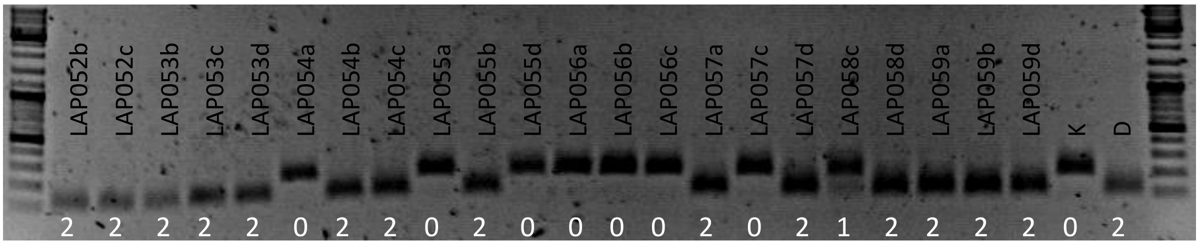

QTL11_FTa1-F2

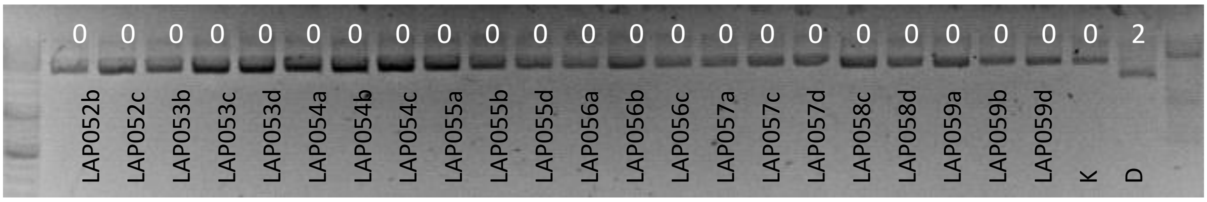

QTL12_SEP3-F1

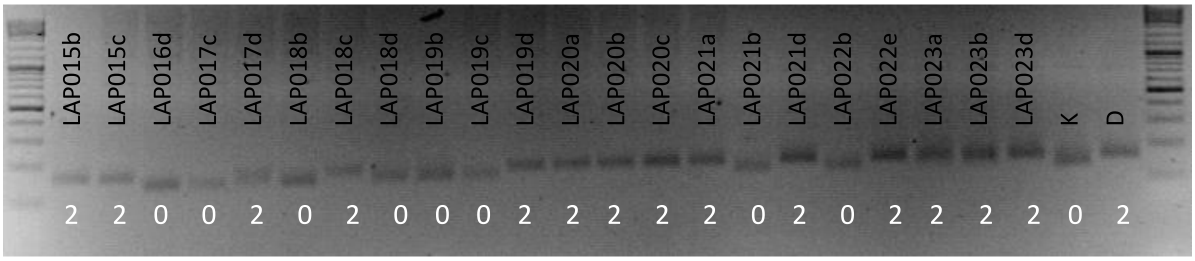

QTL13_TP86766

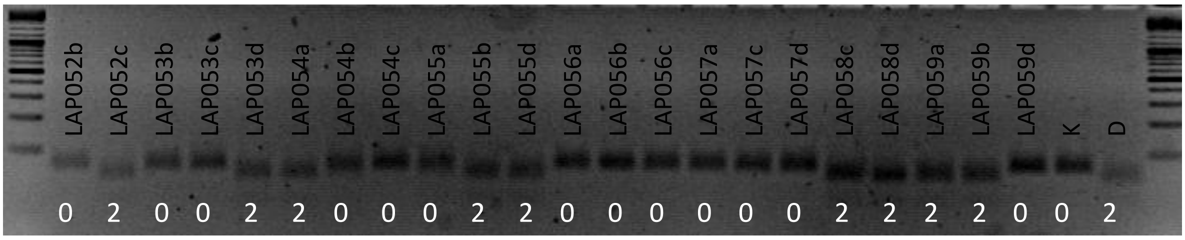

QTL14_PIF4-F6

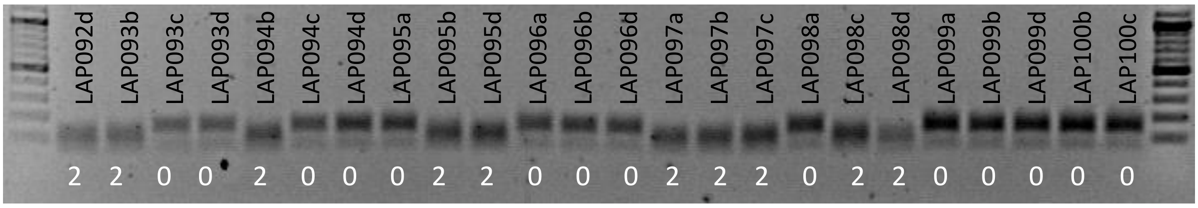

QTL15_TP47110

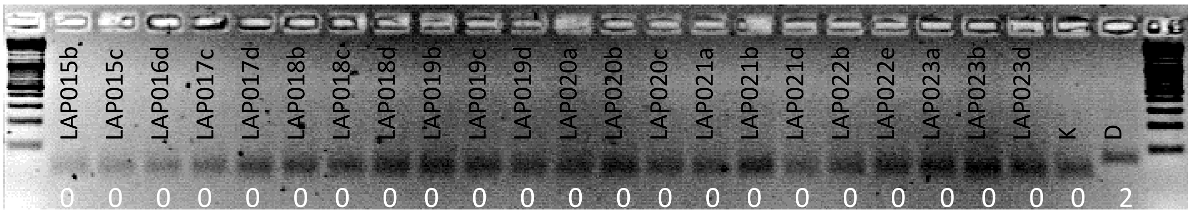

QTL16_TP345457

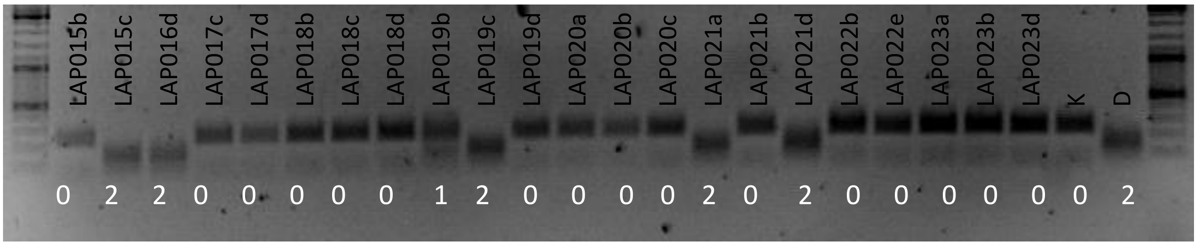

Supplement: Supplementary file 6 — Supplementary Material 6: Supplementary_File_S6.pdf: Agarose gel electrophoregrams showing polymorphism of PCR-based markers tagging white lupin flowering time quantitative trait loci (QTLs) from linkage mapping studies. [file 12870_2024_5438_MOESM6_ESM.pdf]
