# Supplementary material for: A GWAS study highlights significant associations between a series of indels in a FLOWERING LOCUS T gene promoter and flowering time in white lupin (Lupinus albus L.)
Source: BMC Plant Biol. 2024 Jul 29;24:722. doi: 10.1186/s12870-024-05438-1 (PMC11285409; doi:10.1186/s12870-024-05438-1)
Supplement: Supplementary file 7 — Supplementary Material 7: Supplementary_File_S7.pdf: Agarose gel electrophoregrams showing polymorphism of PCR-based markers developed for white lupin LalbFTc1 gene promoter indels. [file 12870_2024_5438_MOESM7_ESM.pdf]

Sandra Rychel-Bielska, Wojciech Bielski, Anna Surma, Paolo Annicchiarico, Jolanta Belter, Bartosz Kozak, Renata Galek, Nathalie Harzic, Michał Książkiewicz

A GWAS study highlights significant associations between a series of indels in a *FLOWERING LOCUS T* gene promoter and flowering time in white lupin (*Lupinus albus* L.)

BMC Plant Biology

Supplementary File S7. Agarose gel electrophoregrams showing polymorphism of PCR-based markers developed for white lupin *LalbFTc1* gene promoter indels.

K –Kiev Mutant; D – P27174; 0 – "reference" allele; 1 – heterozygote; 2 – "variant" allele

PR_30

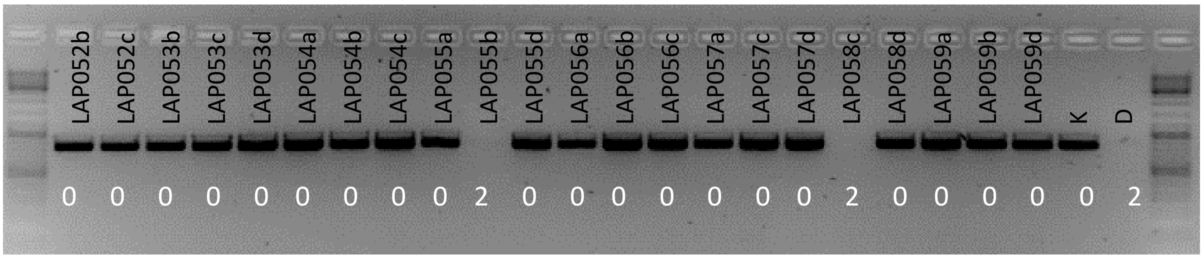


PR_31

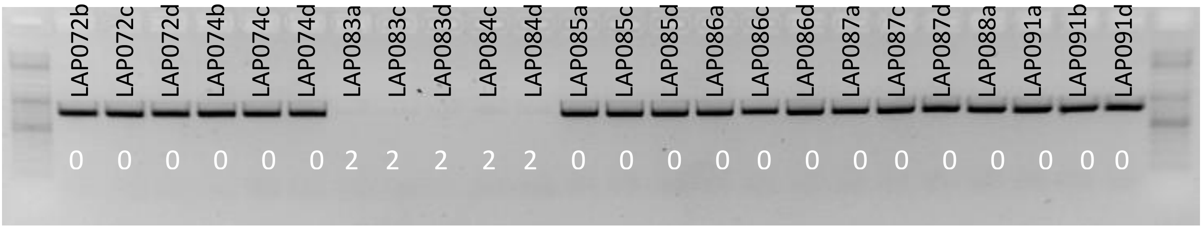


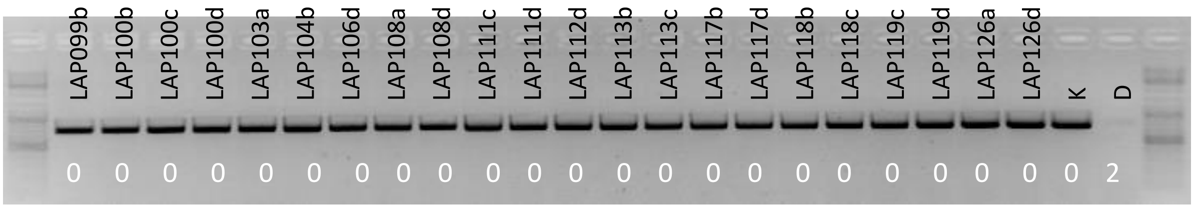


PR_32

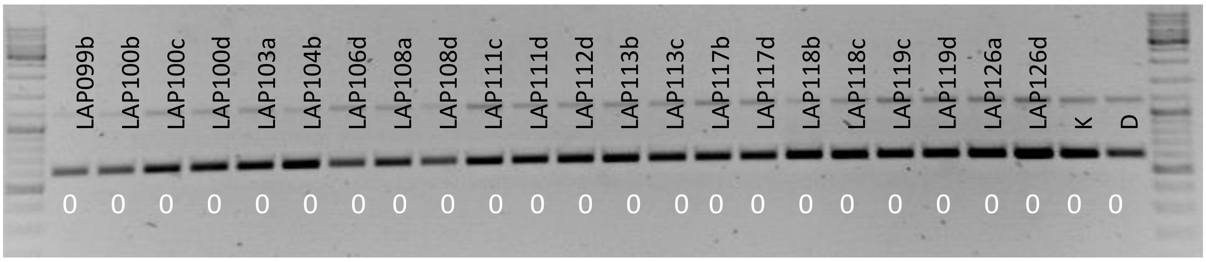

PR_33

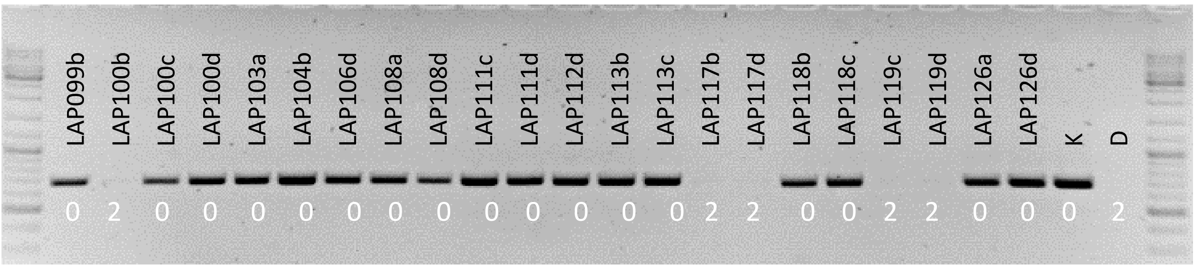


PR_34

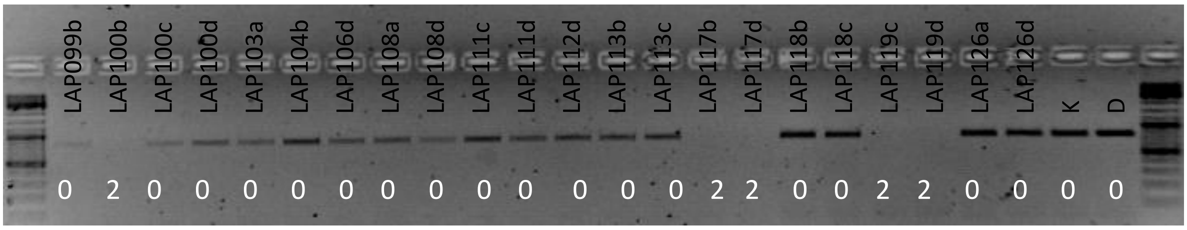


PR_35a

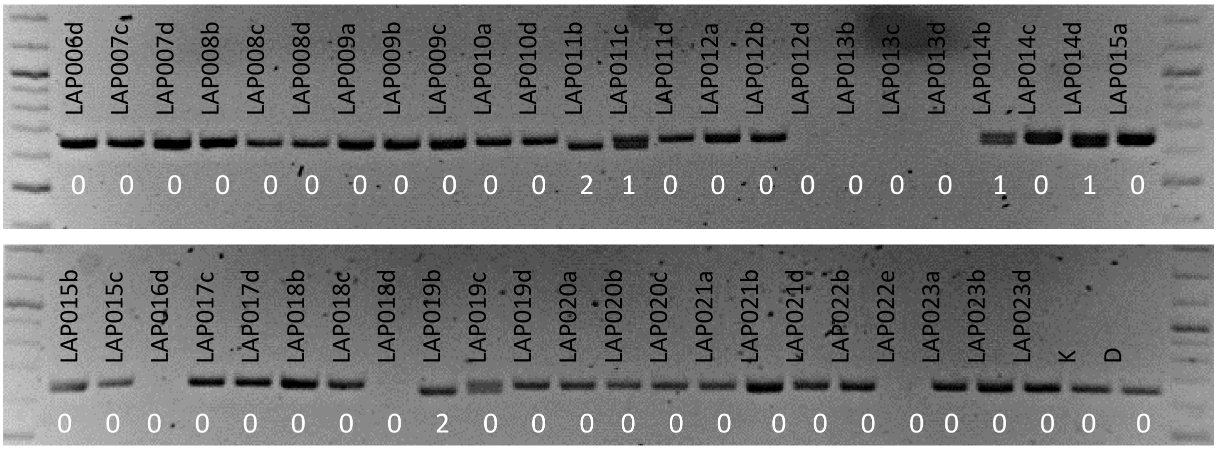


PR_35b

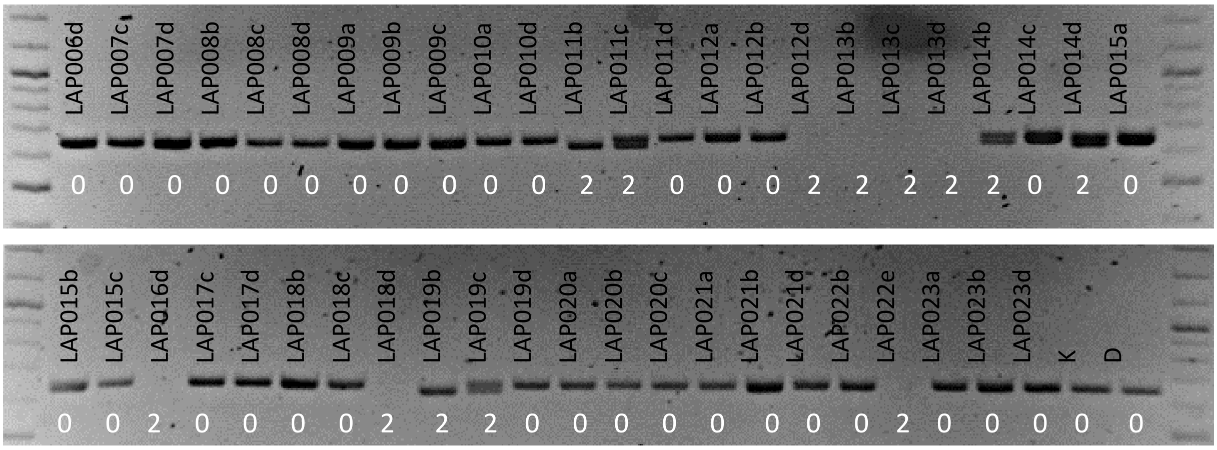

PR_61

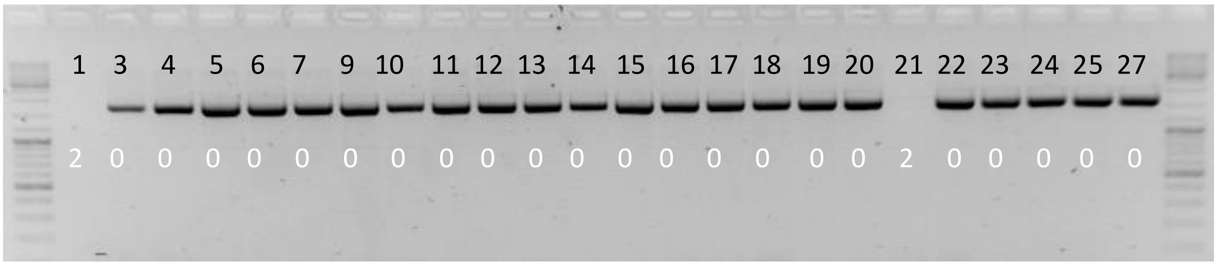


PR_36a

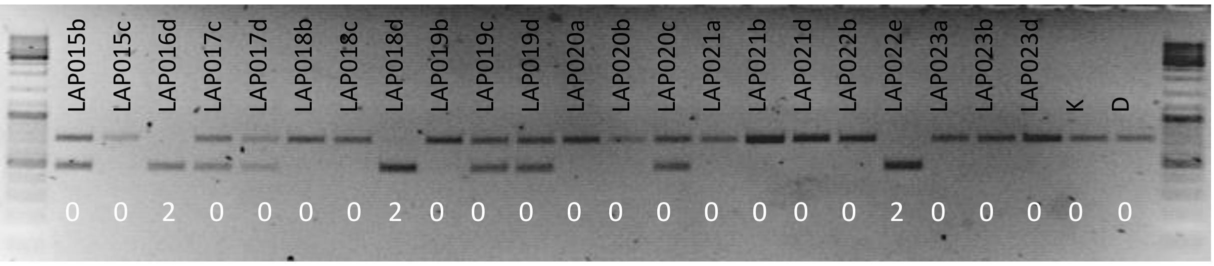


PR_36b

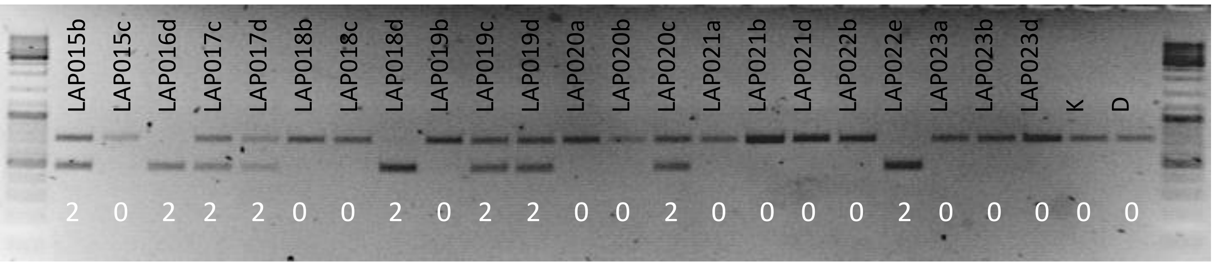


PR_37

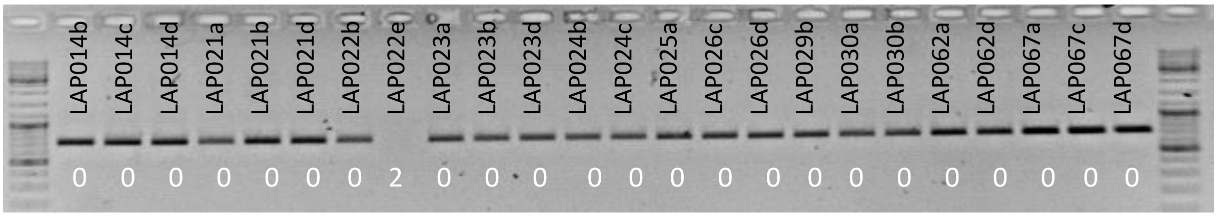


PR_38

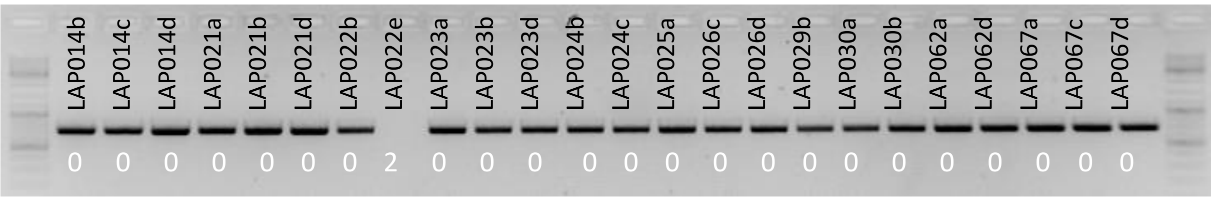

PR_39

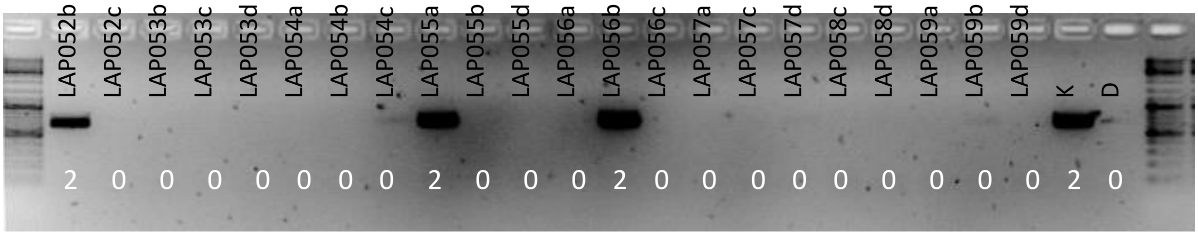


PR_40

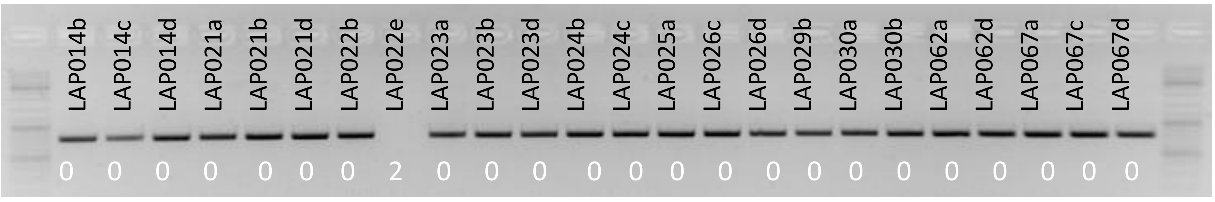


PR_41

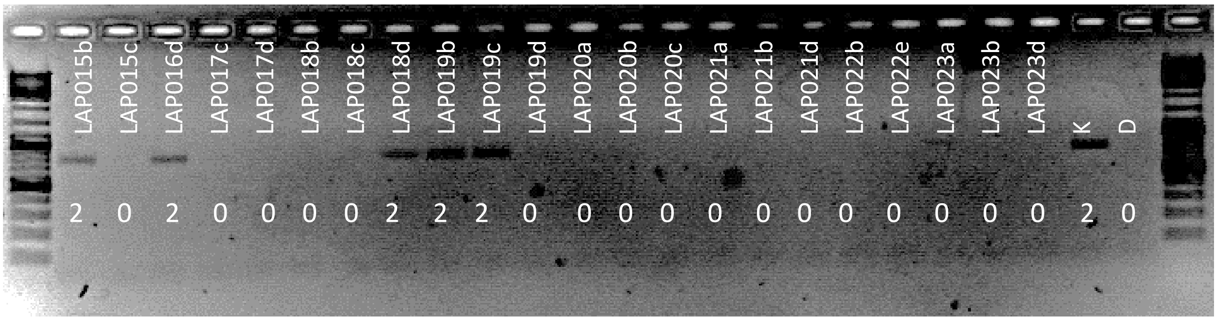


PR_42a

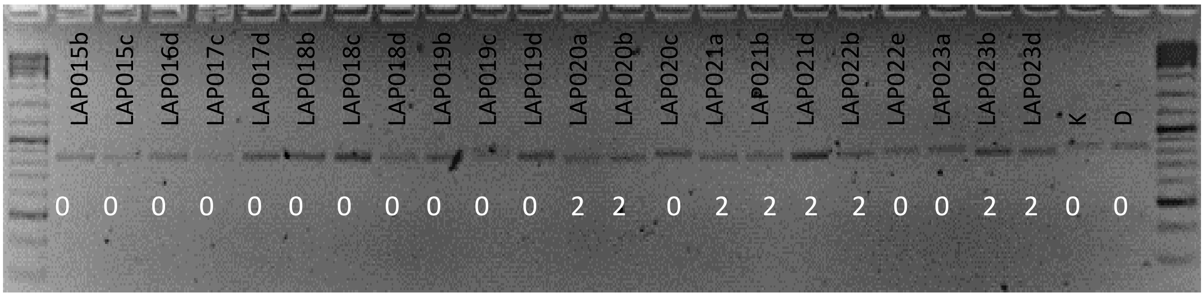


PR_42b

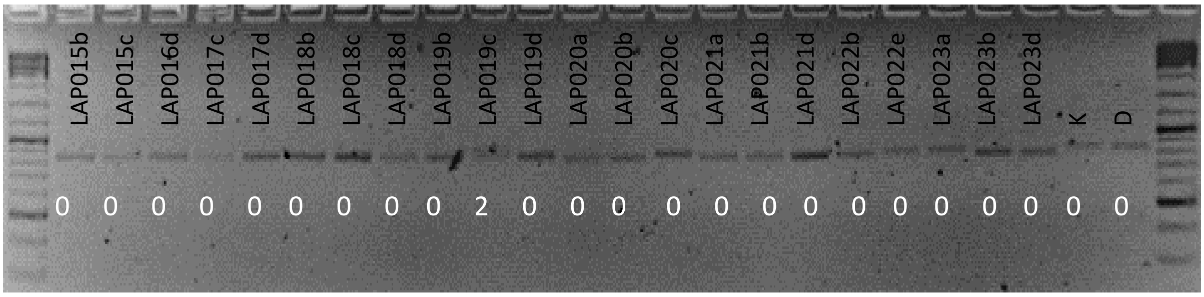

PR_43

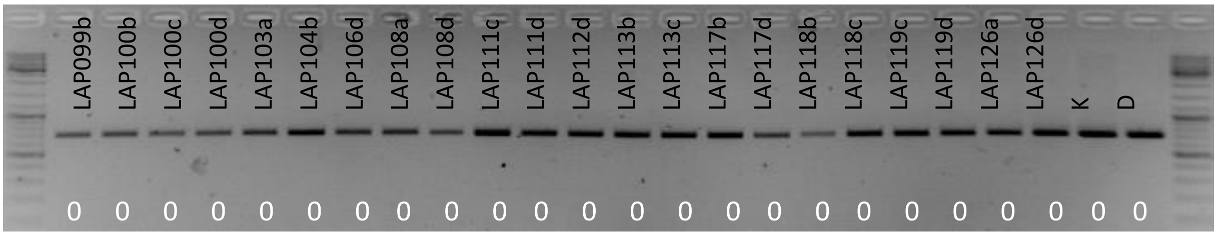


PR_58a

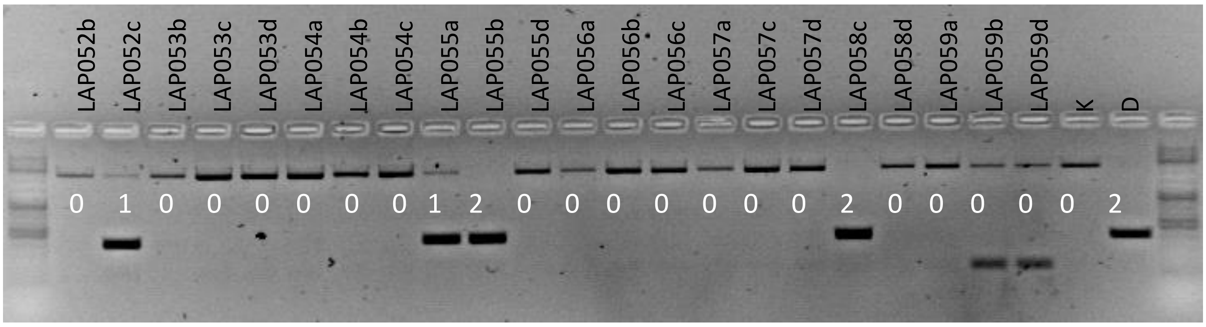


PR_58b

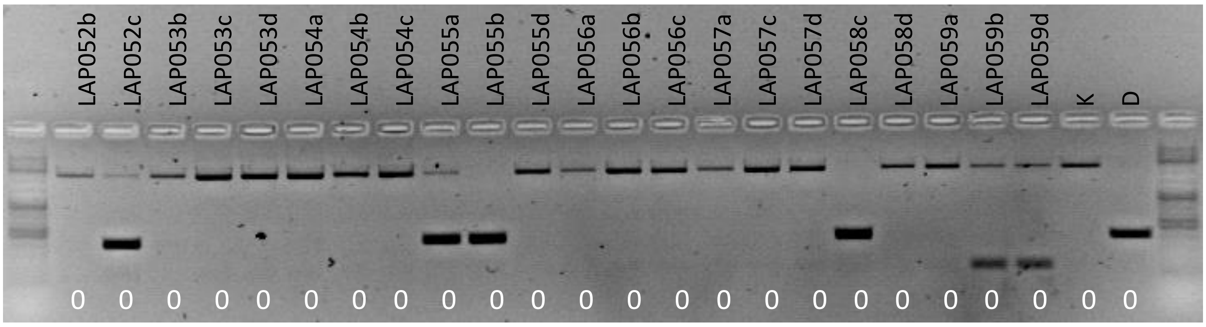


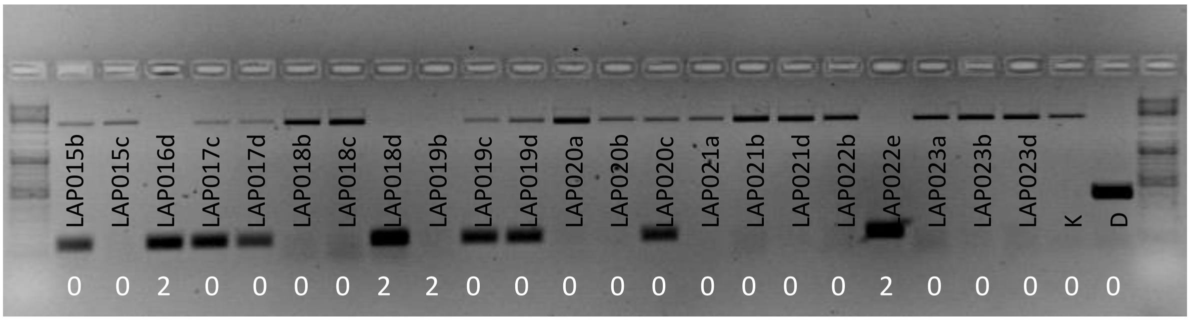

PR_58c

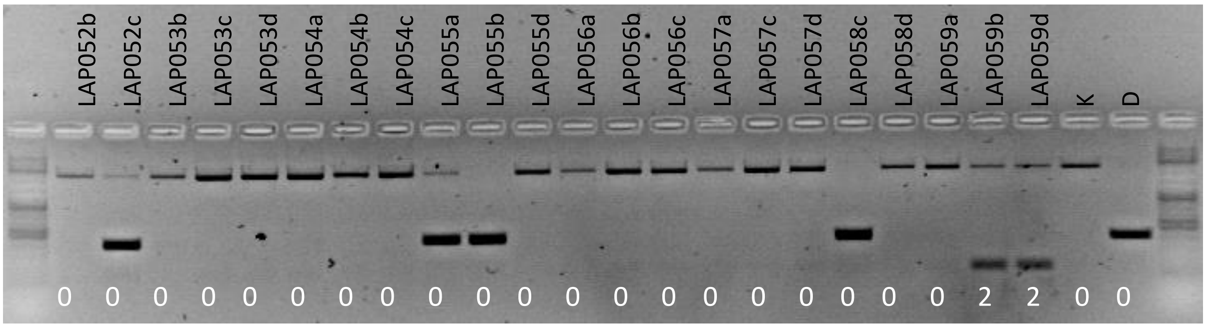


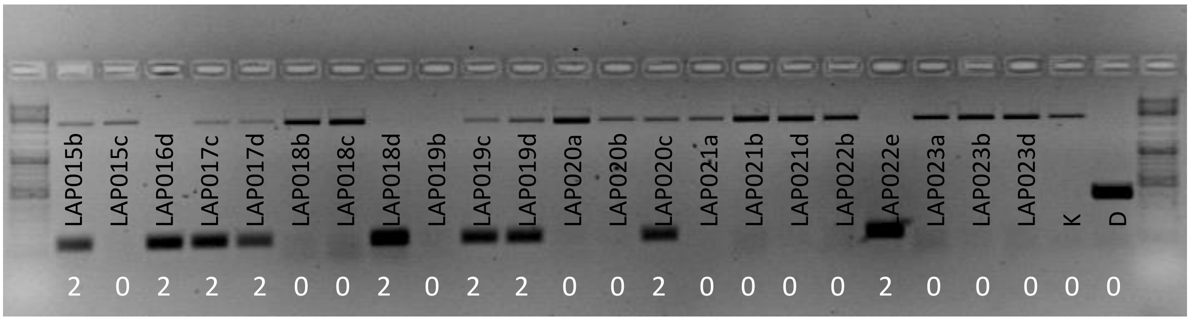


PR_70

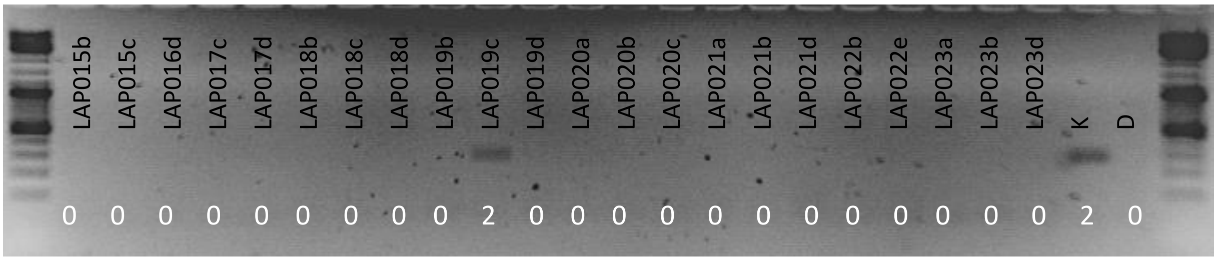


PR_71a

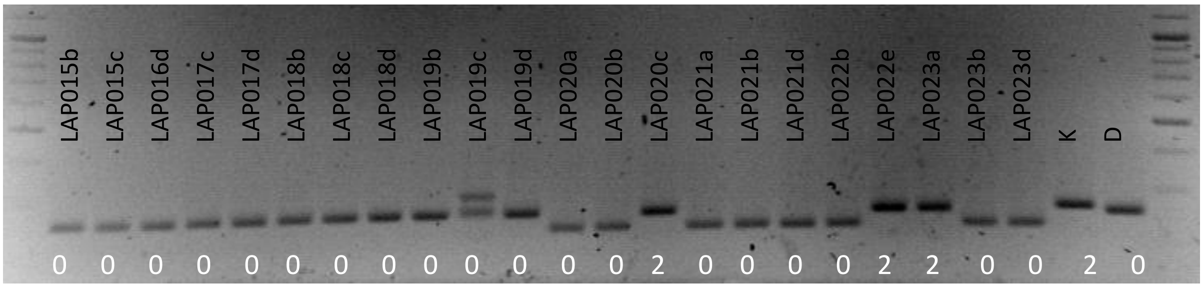


PR_71b

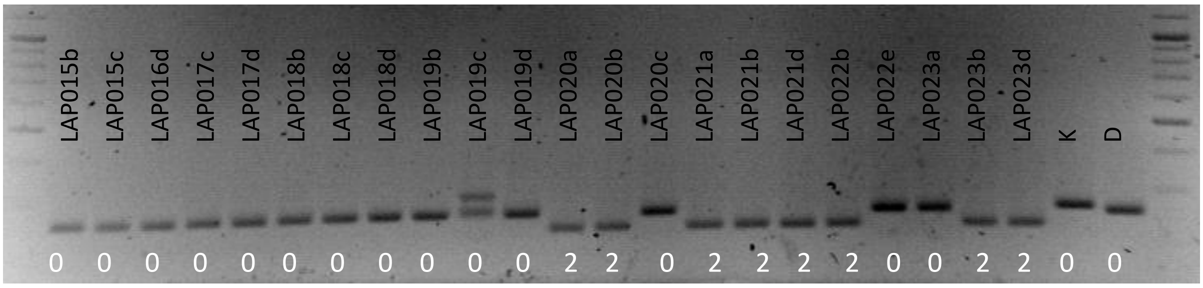

PR_71c

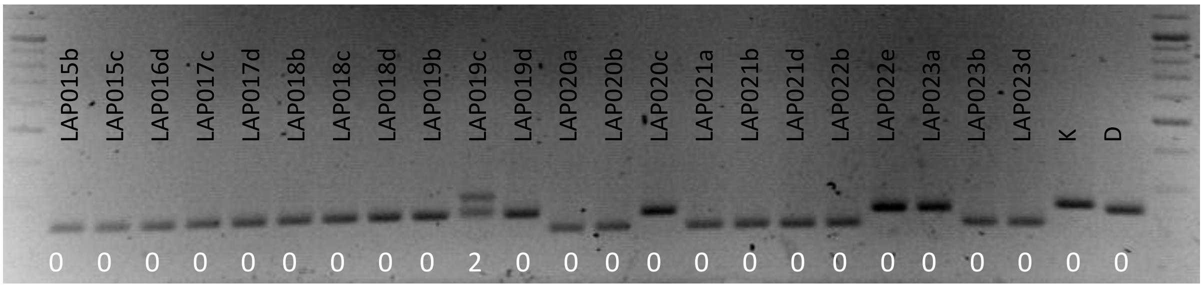
LAP015b
LAP015c
LAP016d
LAP017c
LAP017d
LAP018b
LAP018c
LAP018d
LAP019b
LAP019c
LAP019d
LAP020a
LAP020b
LAP020c
LAP021a
LAP021b
LAP021d
LAP022b
LAP022e
LAP023a
LAP023b
LAP023d
K
D
0 0 0 0 0 0 0 0 0 2 0 0 0 0 0 0 0 0 0 0 0 0 0 0

PR_71d

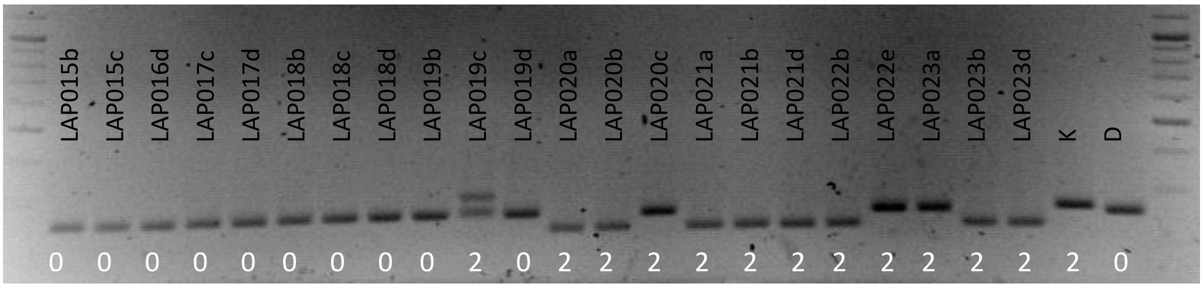
LAP015b
LAP015c
LAP016d
LAP017c
LAP017d
LAP018b
LAP018c
LAP018d
LAP019b
LAP019c
LAP019d
LAP020a
LAP020b
LAP020c
LAP021a
LAP021b
LAP021d
LAP022b
LAP022e
LAP023a
LAP023b
LAP023d
K
D
0 0 0 0 0 0 0 0 0 2 0 2 2 2 2 2 2 2 2 2 2 2 2 0
