# Supplementary material for: A GWAS study highlights significant associations between a series of indels in a FLOWERING LOCUS T gene promoter and flowering time in white lupin (Lupinus albus L.)
Source: BMC Plant Biol. 2024 Jul 29;24:722. doi: 10.1186/s12870-024-05438-1 (PMC11285409; doi:10.1186/s12870-024-05438-1)

Sandra Rychel-Bielska, Wojciech Bielski, Anna Surma, Paolo Annicchiarico, Jolanta Belter, Bartosz Kozak, Renata Galek, Nathalie Harzic, Michał Książkiewicz

A GWAS study highlights significant associations between a series of indels in a *FLOWERING LOCUS T* gene promoter and flowering time in white lupin (*Lupinus albus* L.)

BMC Plant Biology

Supplementary File S8. Values of the cross-entropy criterion for a number clusters ranging from K1 to K30.

6765 markers obtained for 262 white lupin genotypes were used for calculations.

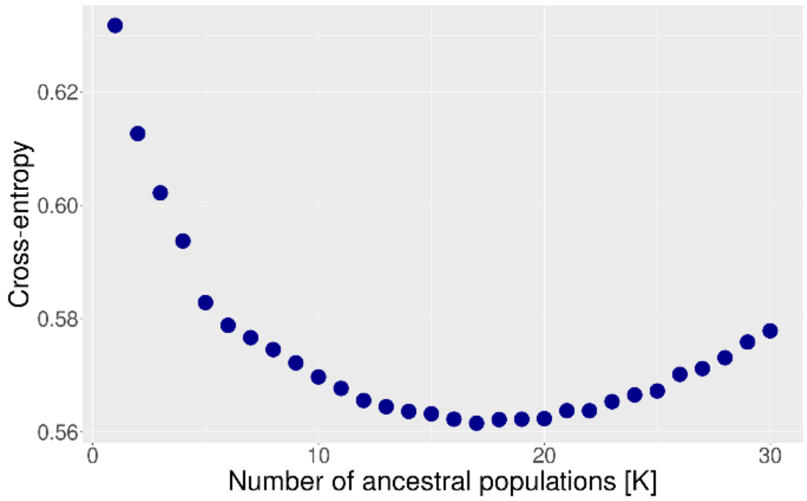

Supplement: Supplementary file 8 — Supplementary Material 8: Supplementary_File_S8.pdf: Values of the cross-entropy criterion for a number clusters ranging from K1 to K30. [file 12870_2024_5438_MOESM8_ESM.pdf]
