# Supplementary material for: A GWAS study highlights significant associations between a series of indels in a FLOWERING LOCUS T gene promoter and flowering time in white lupin (Lupinus albus L.)
Source: BMC Plant Biol. 2024 Jul 29;24:722. doi: 10.1186/s12870-024-05438-1 (PMC11285409; doi:10.1186/s12870-024-05438-1)

Sandra Rychel-Bielska, Wojciech Bielski, Anna Surma, Paolo Annicchiarico, Jolanta Belter, Bartosz Kozak, Renata Galek, Nathalie Harzic, Michał Książkiewicz

A GWAS study highlights significant associations between a series of indels in a *FLOWERING LOCUS T* gene promoter and flowering time in white lupin (*Lupinus albus* L.)

BMC Plant Biology

Supplementary File S16. Full-length original gel images for cropped gels displayed in Supplementary Files.

QTL1

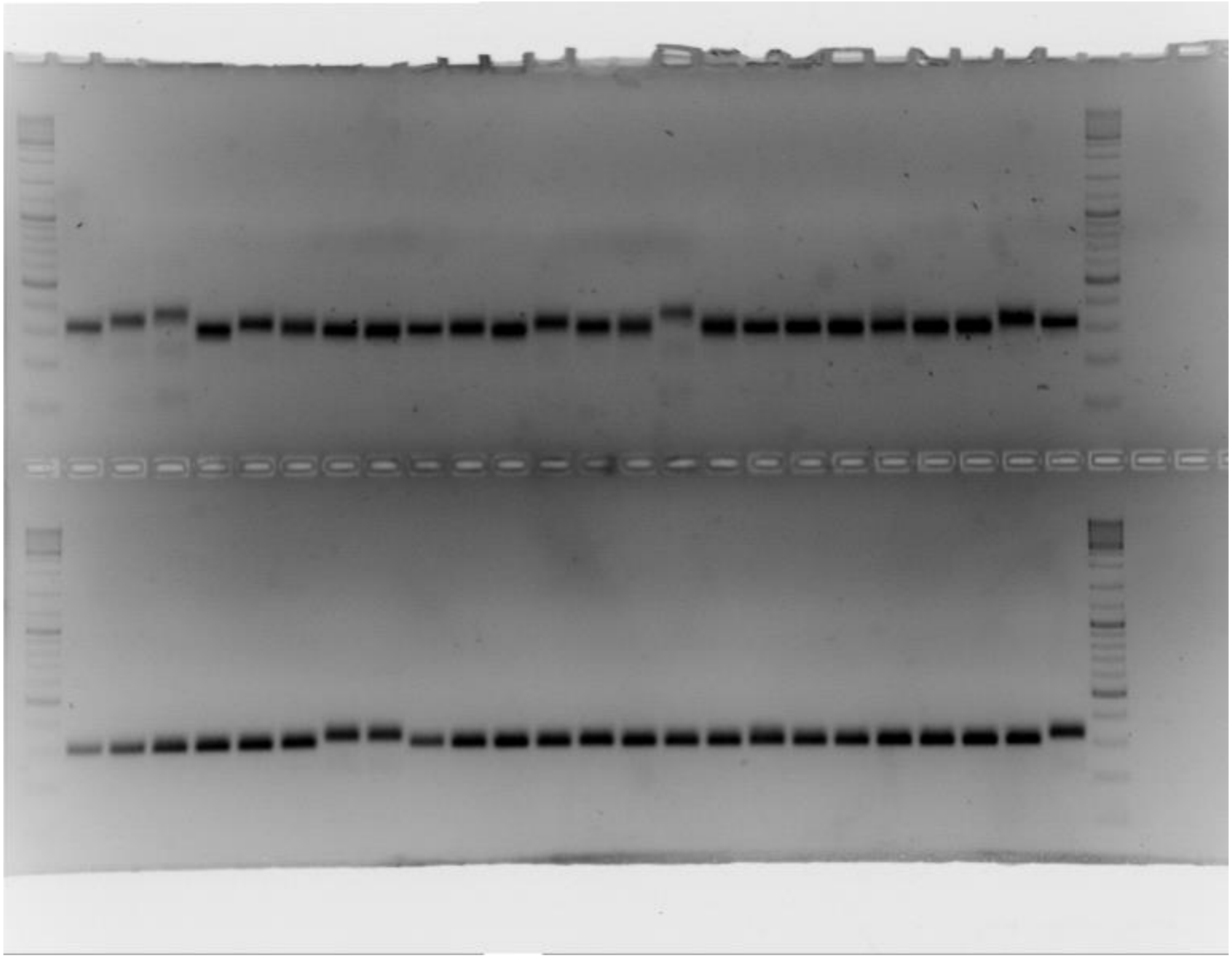

QTL2

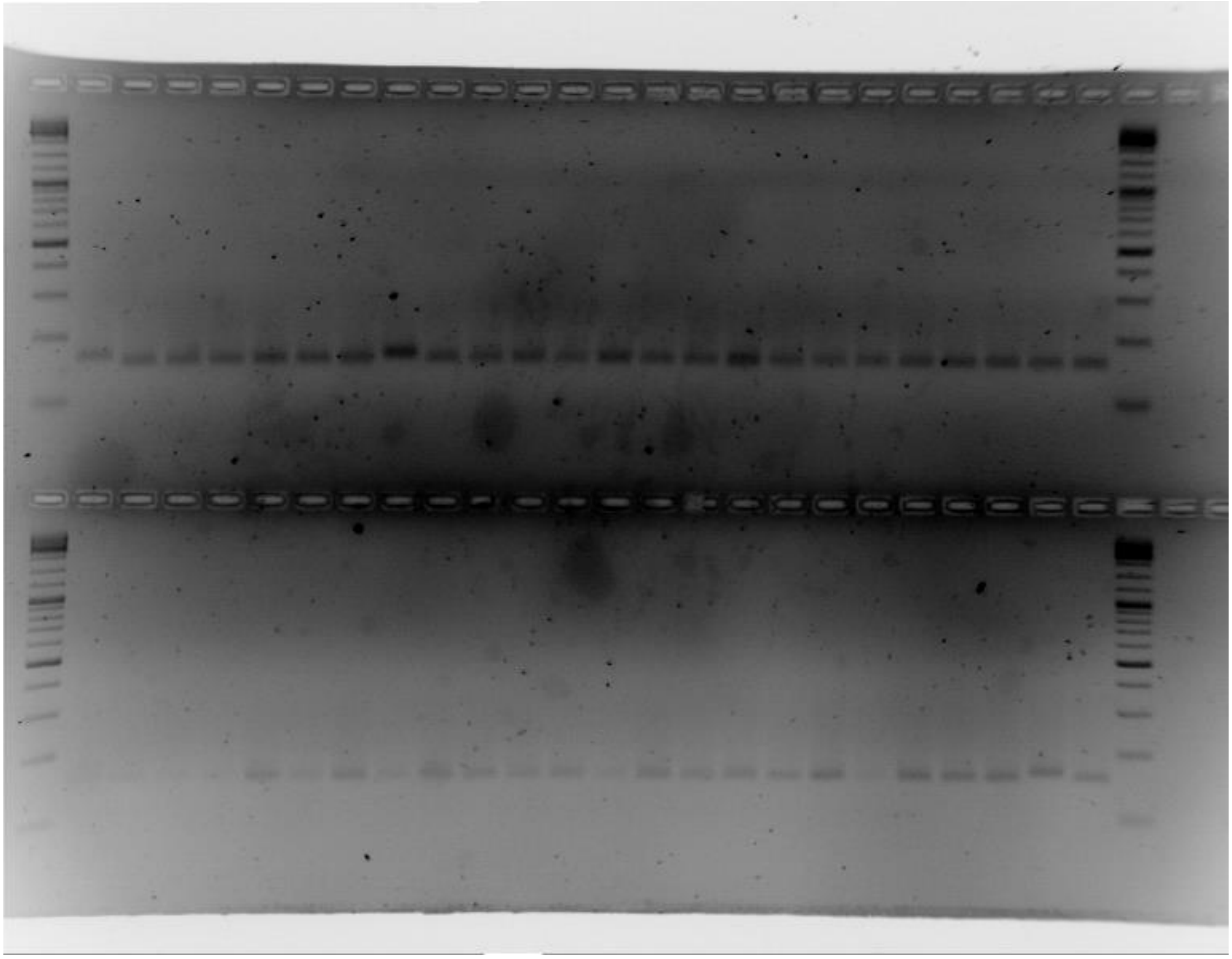

QTL3

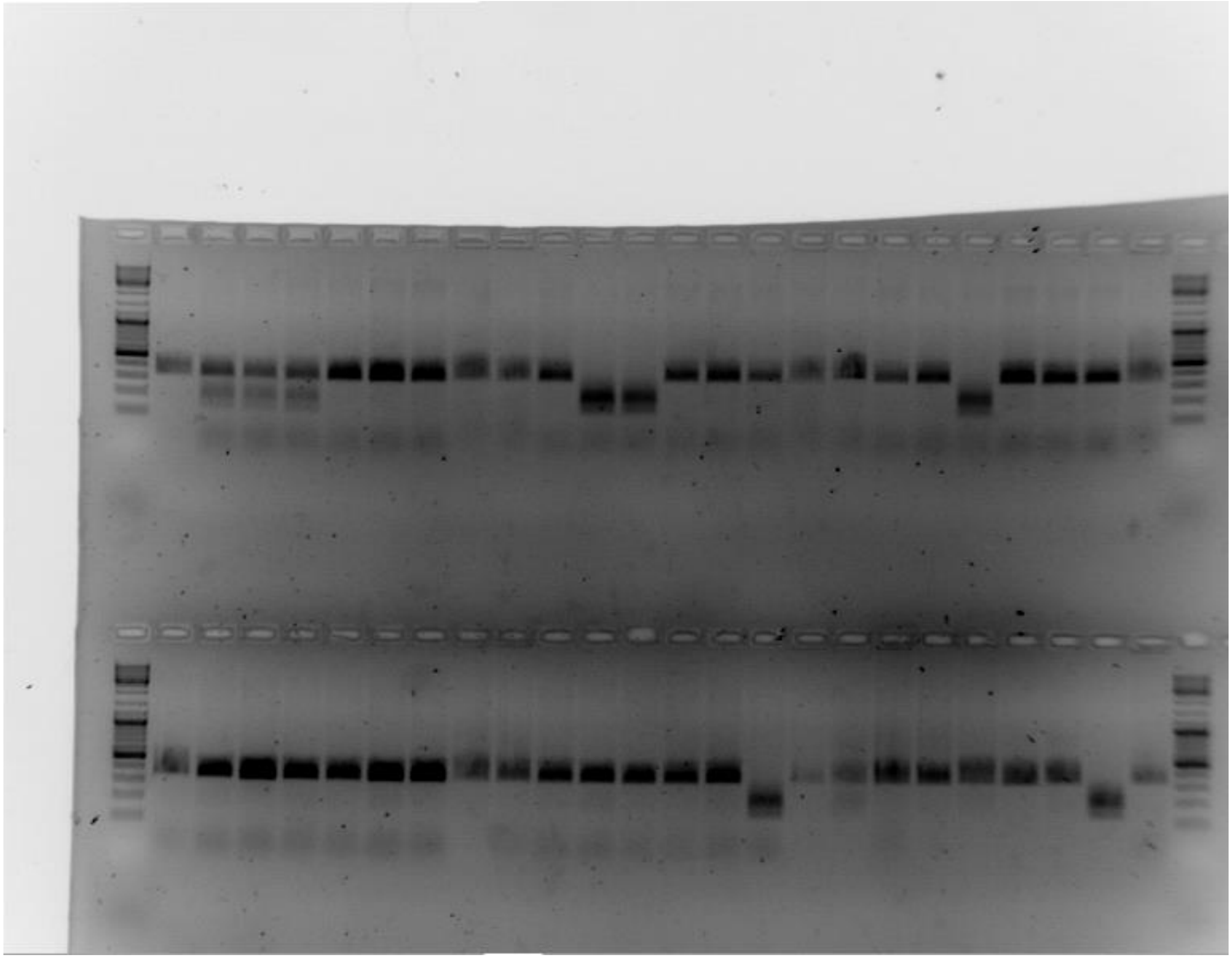

QTL5

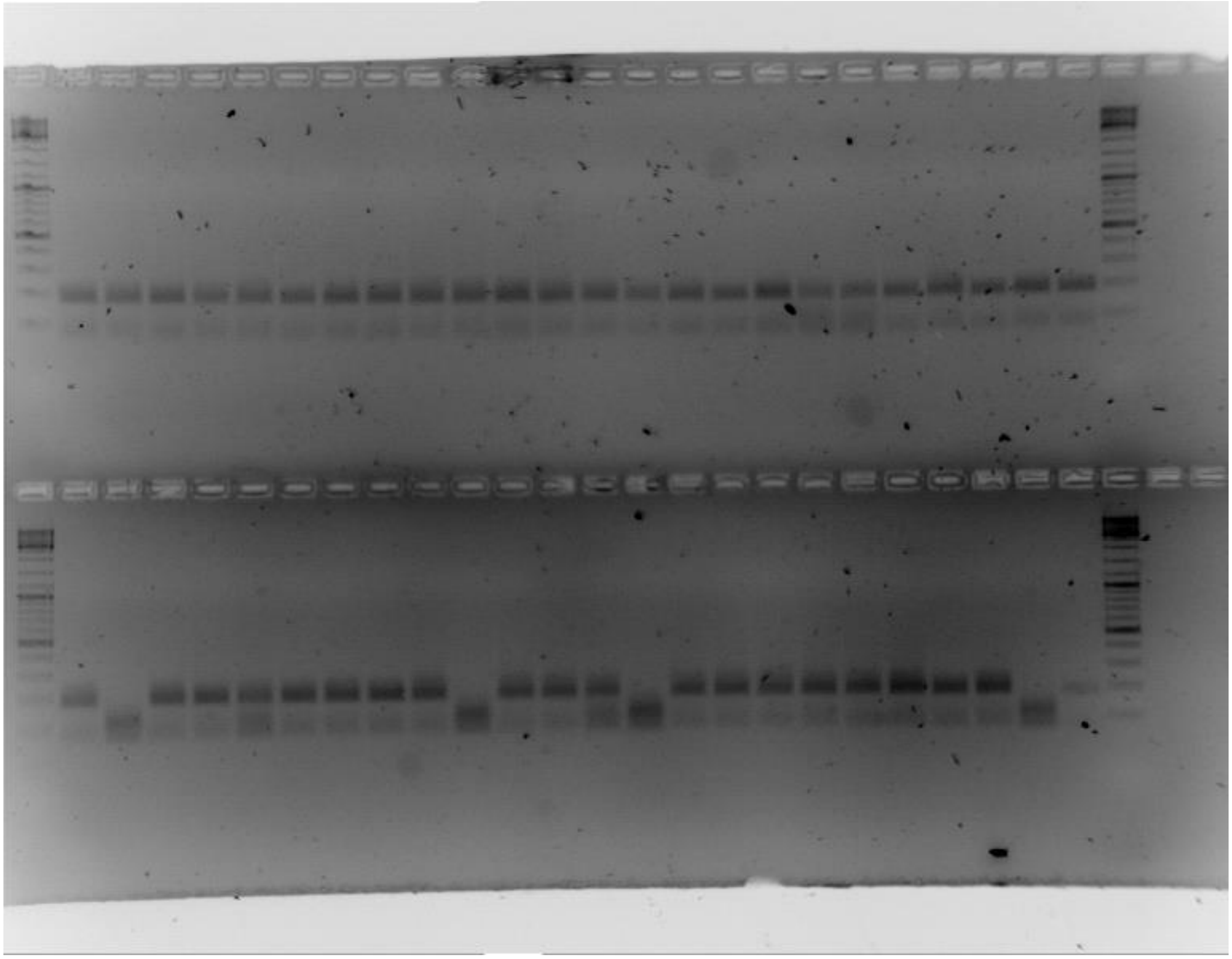

QTL6

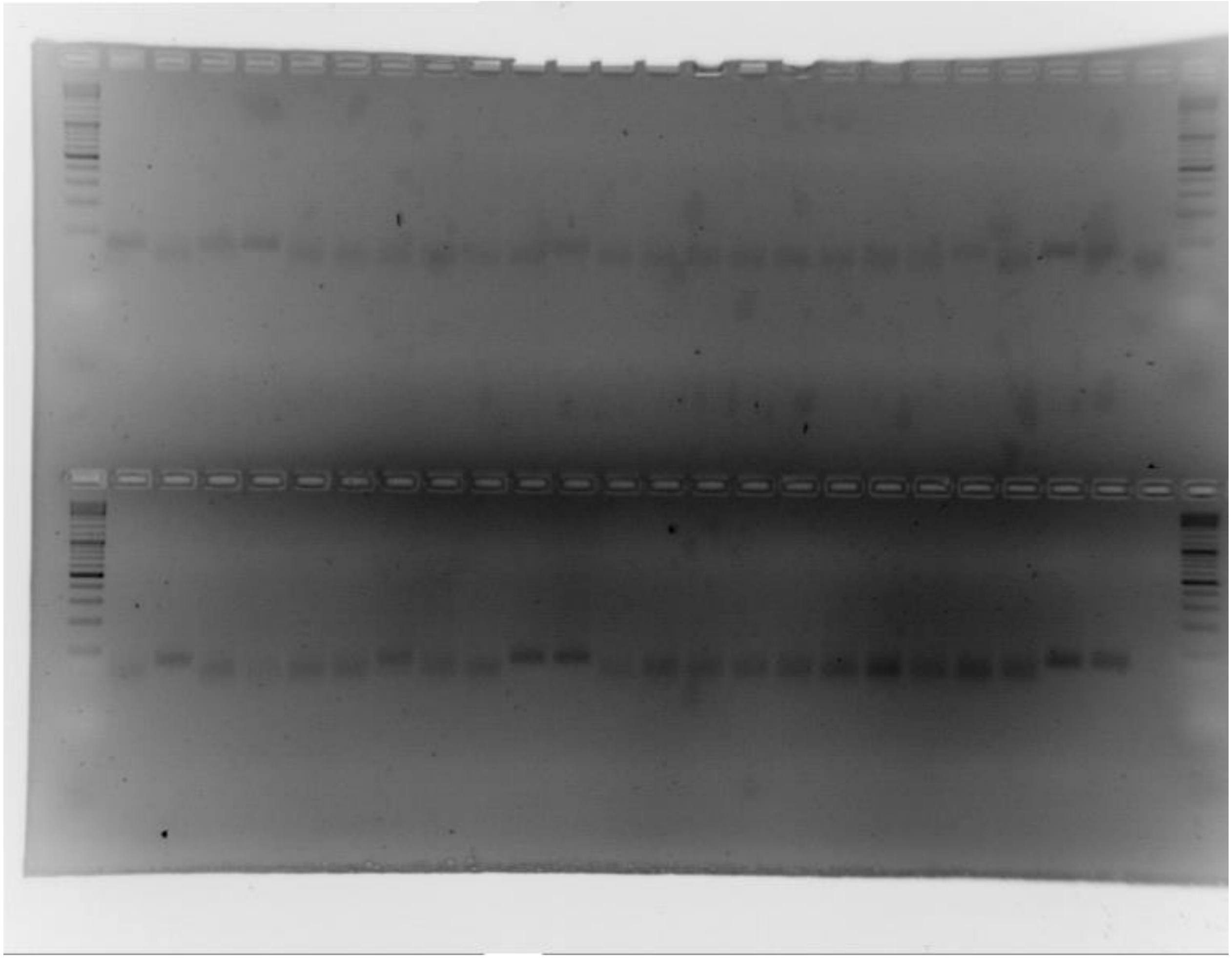

QTL7

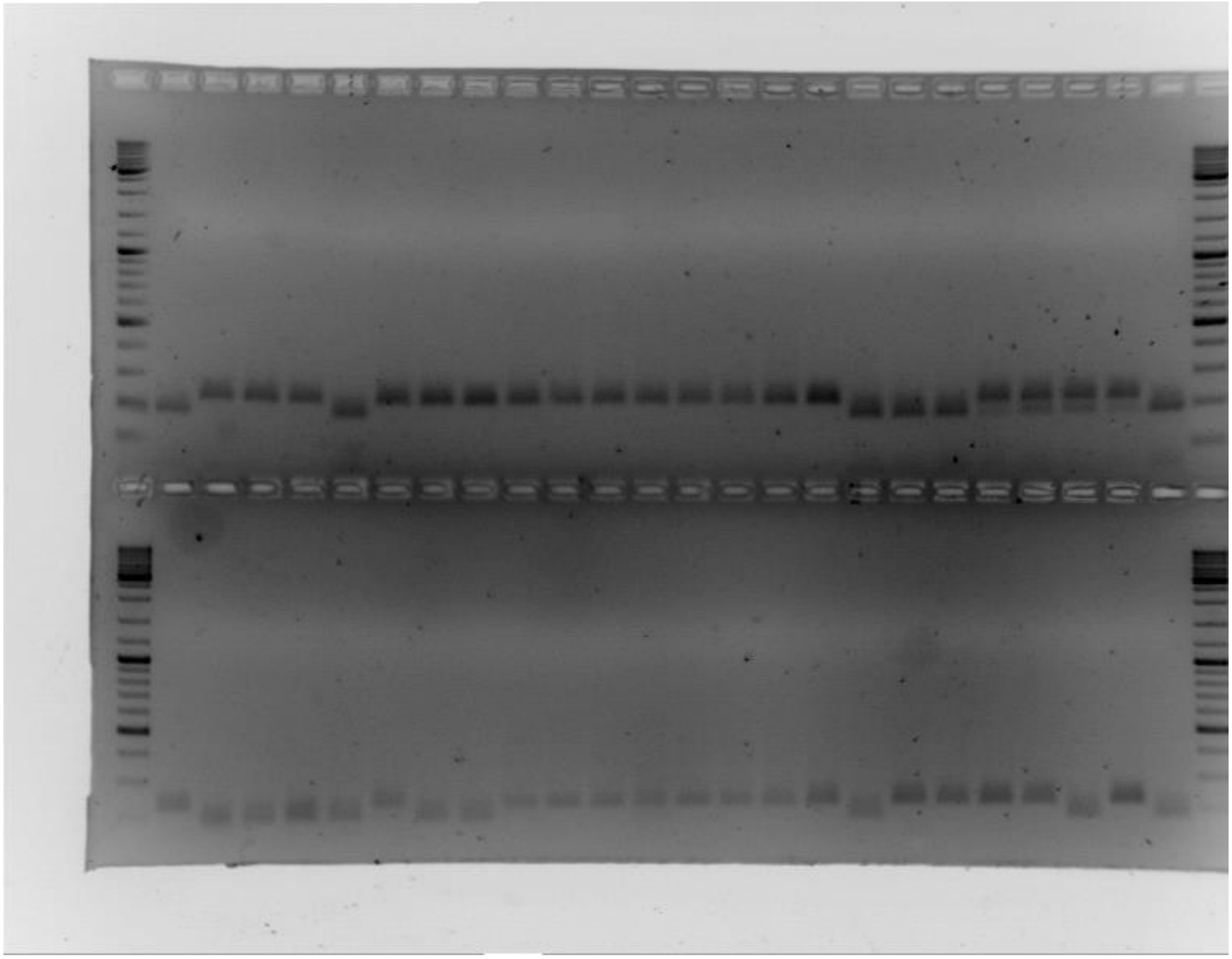

QTL8

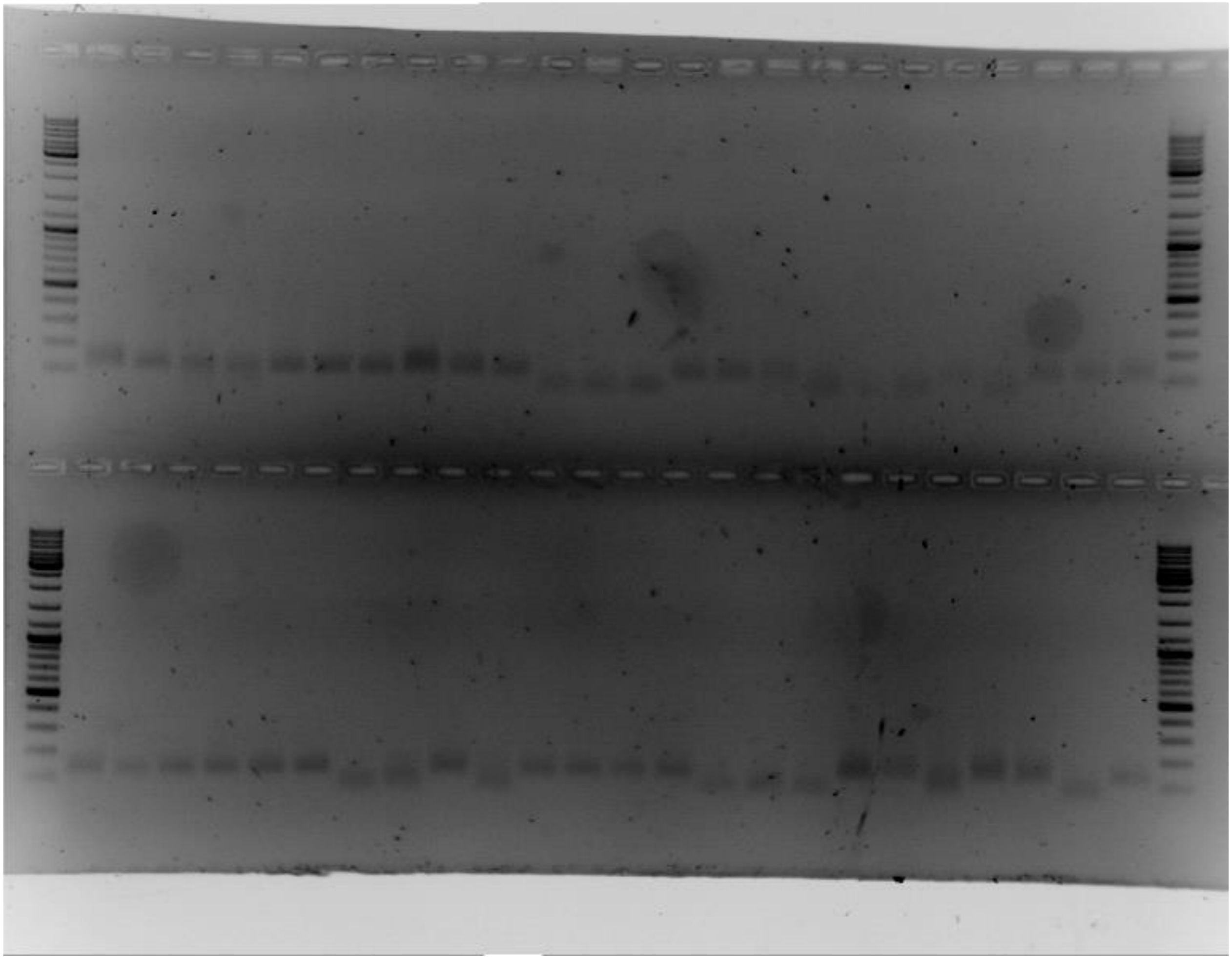

QTL9

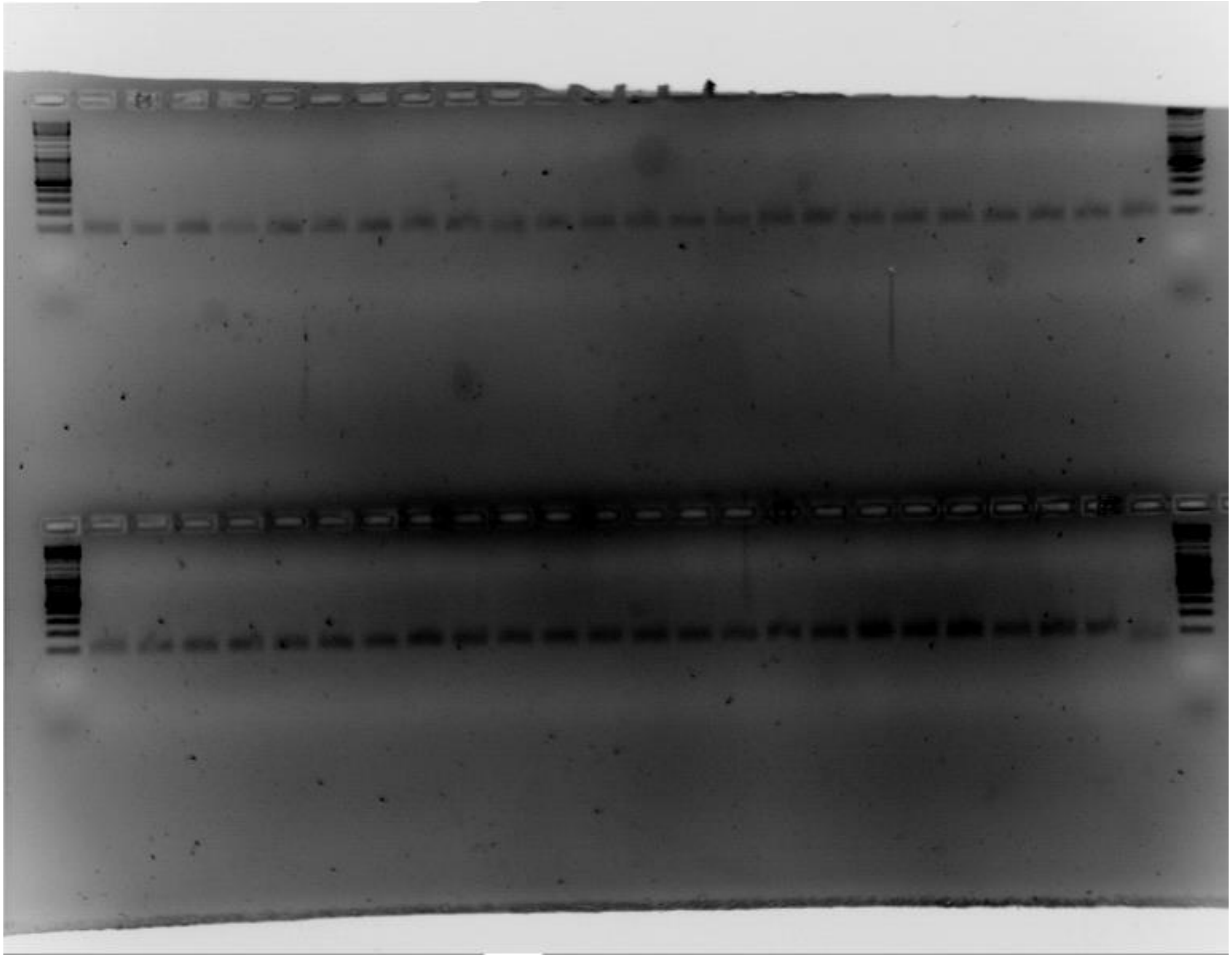

QTL10

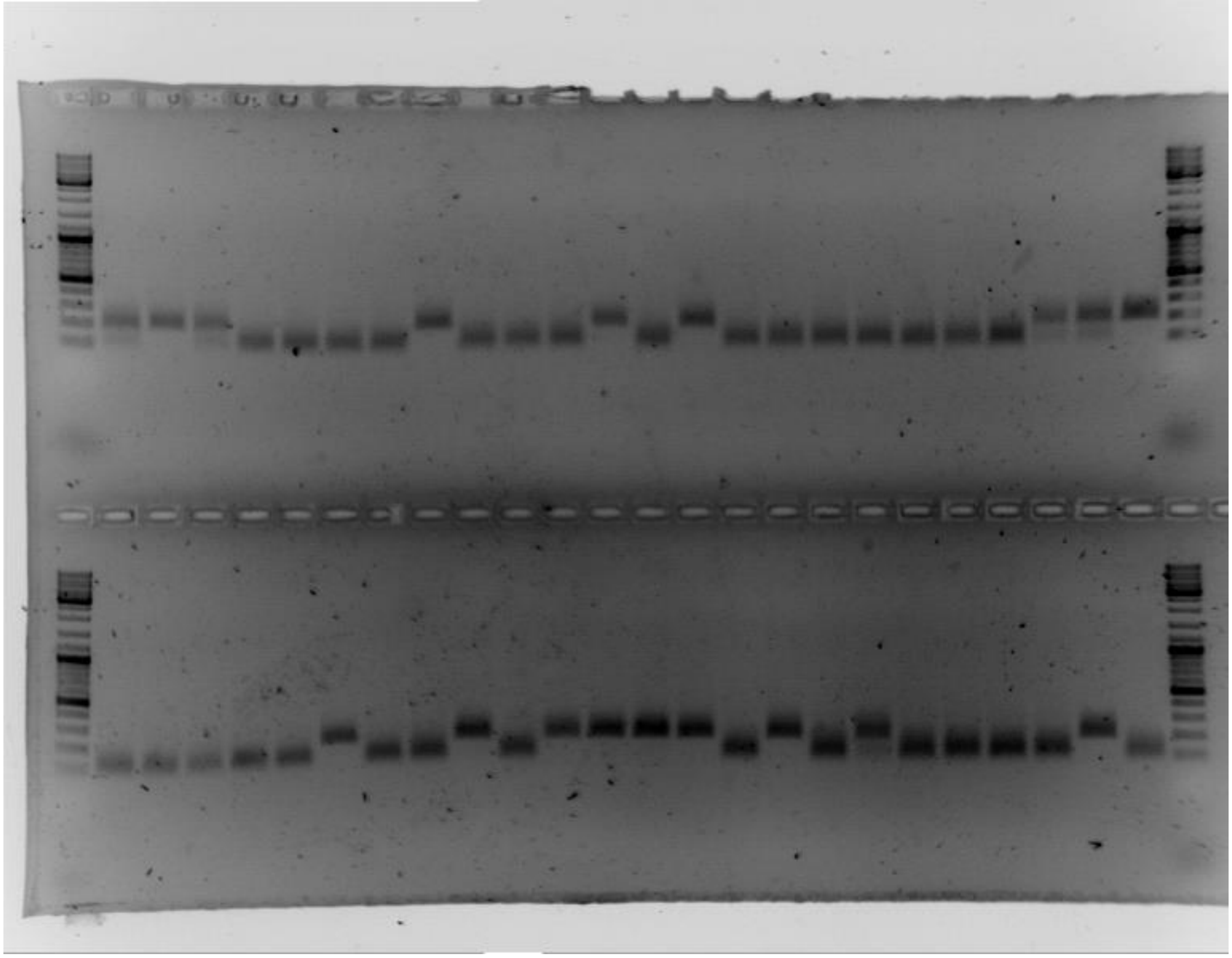

QTL11

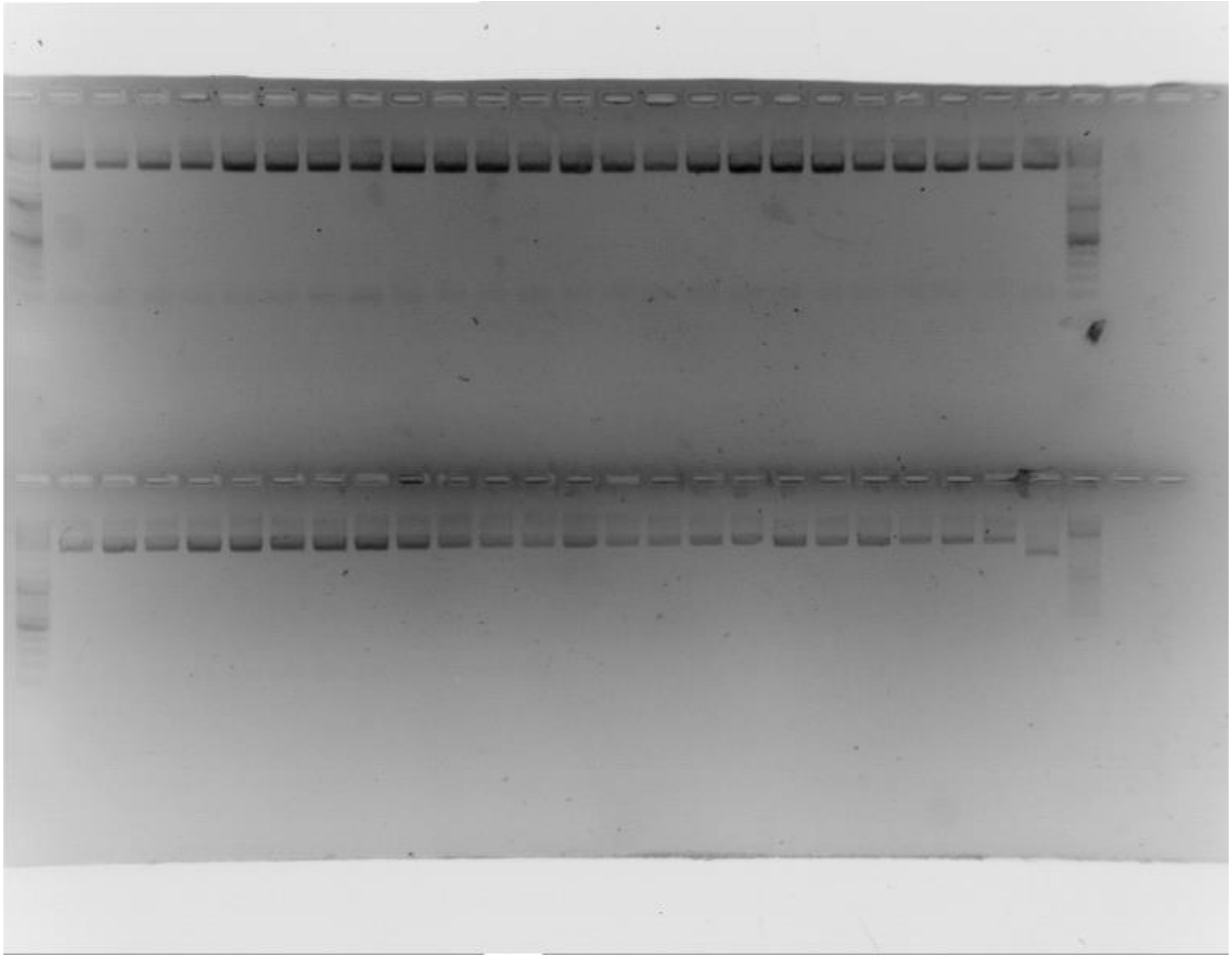

QTL12

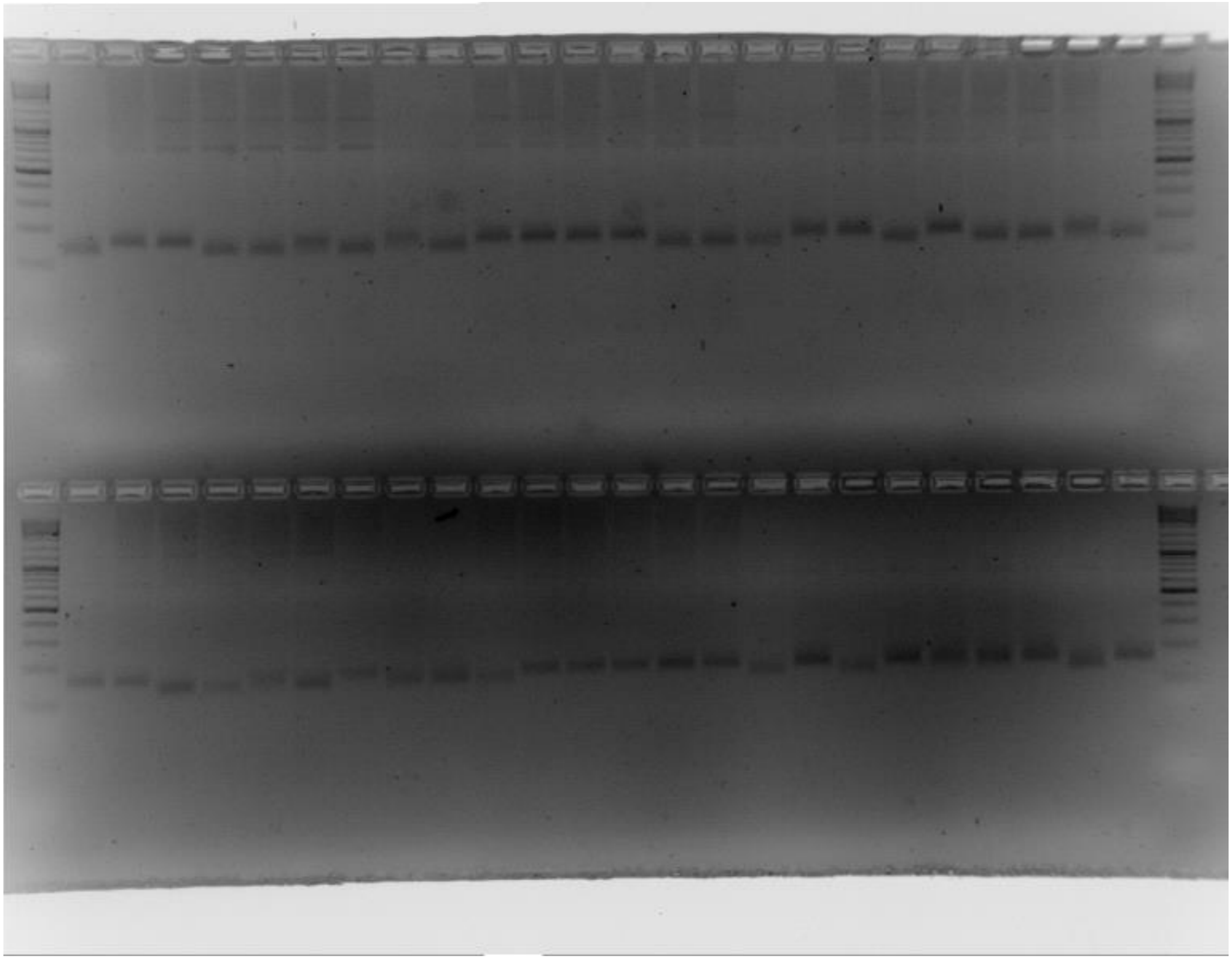

QTL13

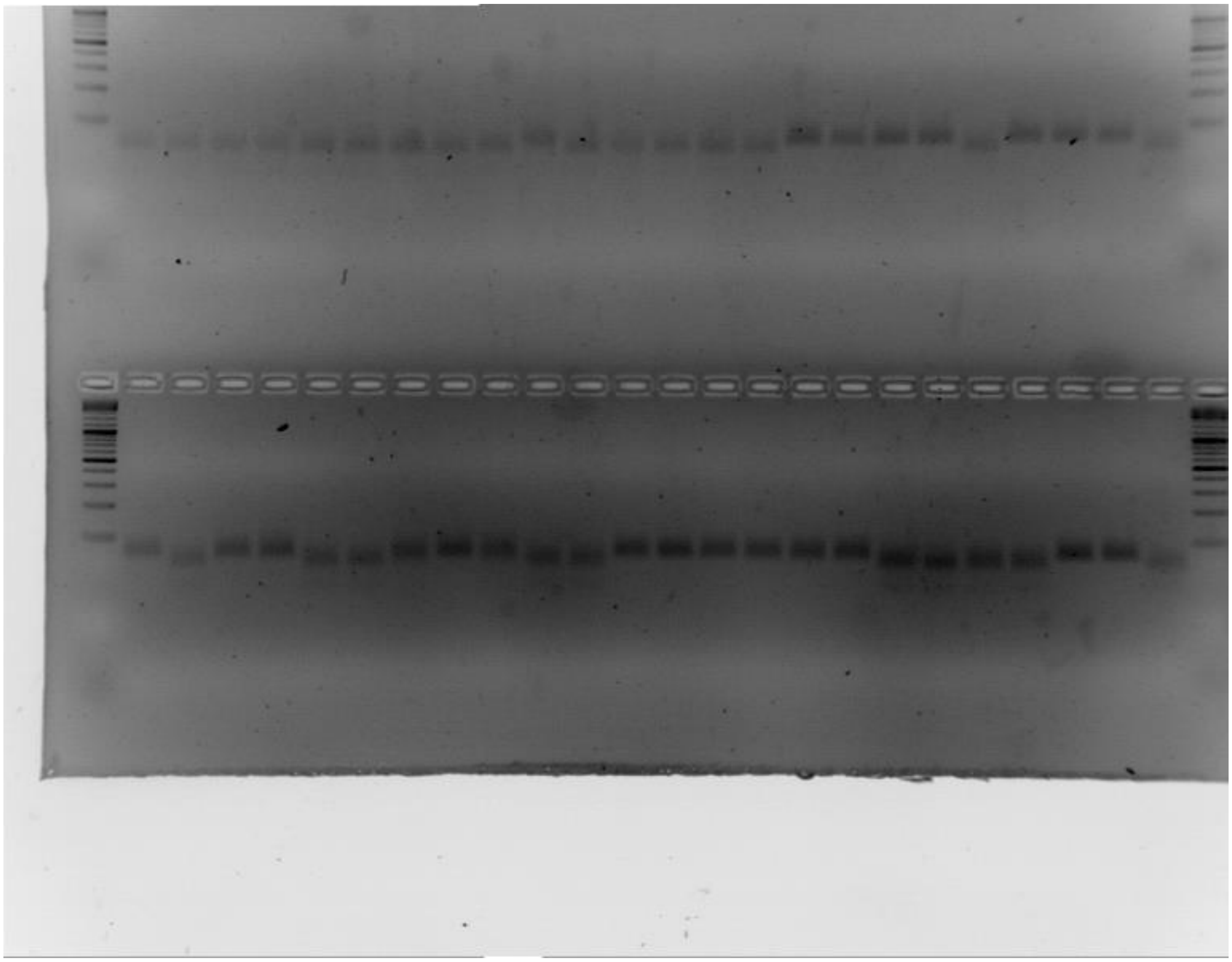

QTL14

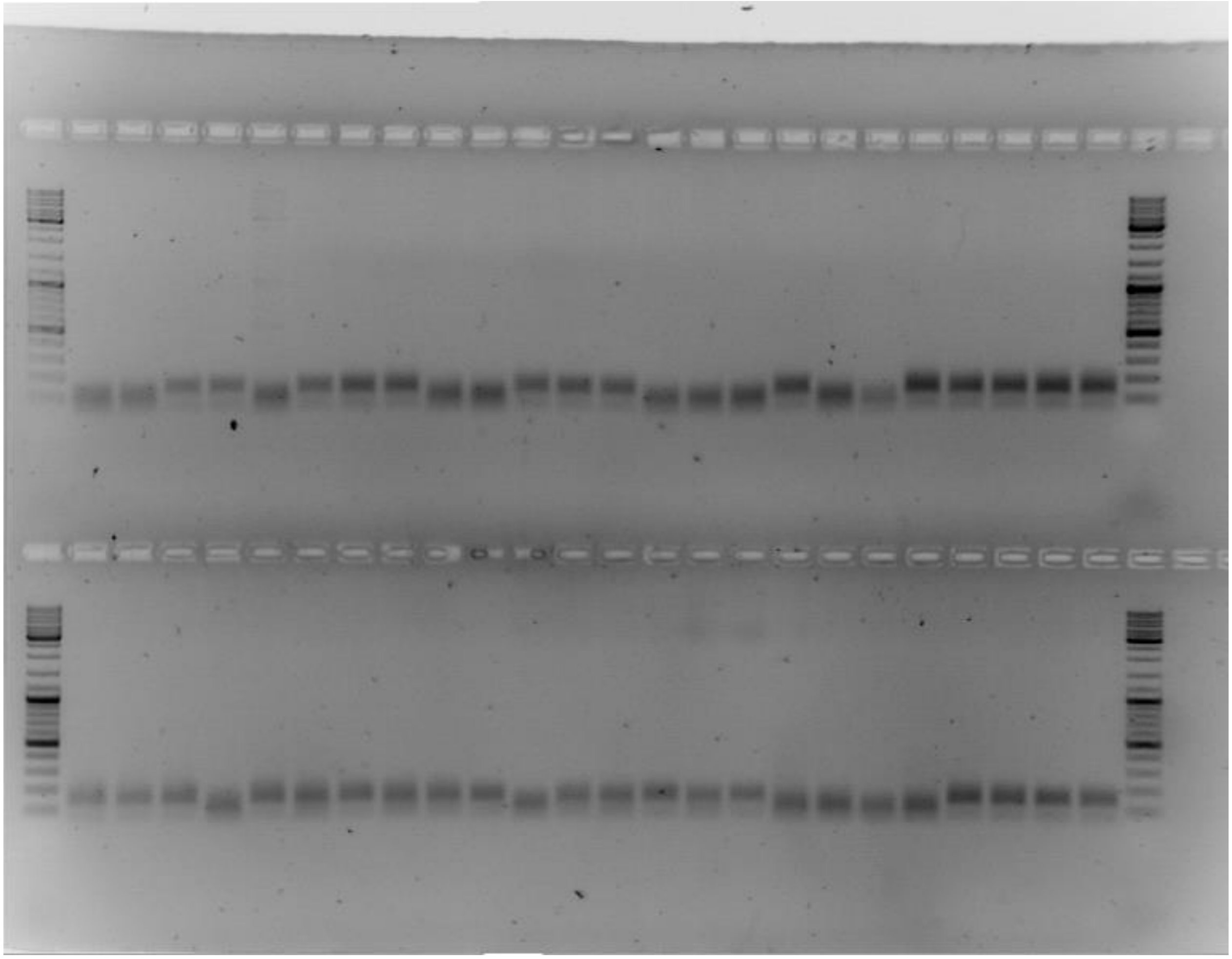

QTL15

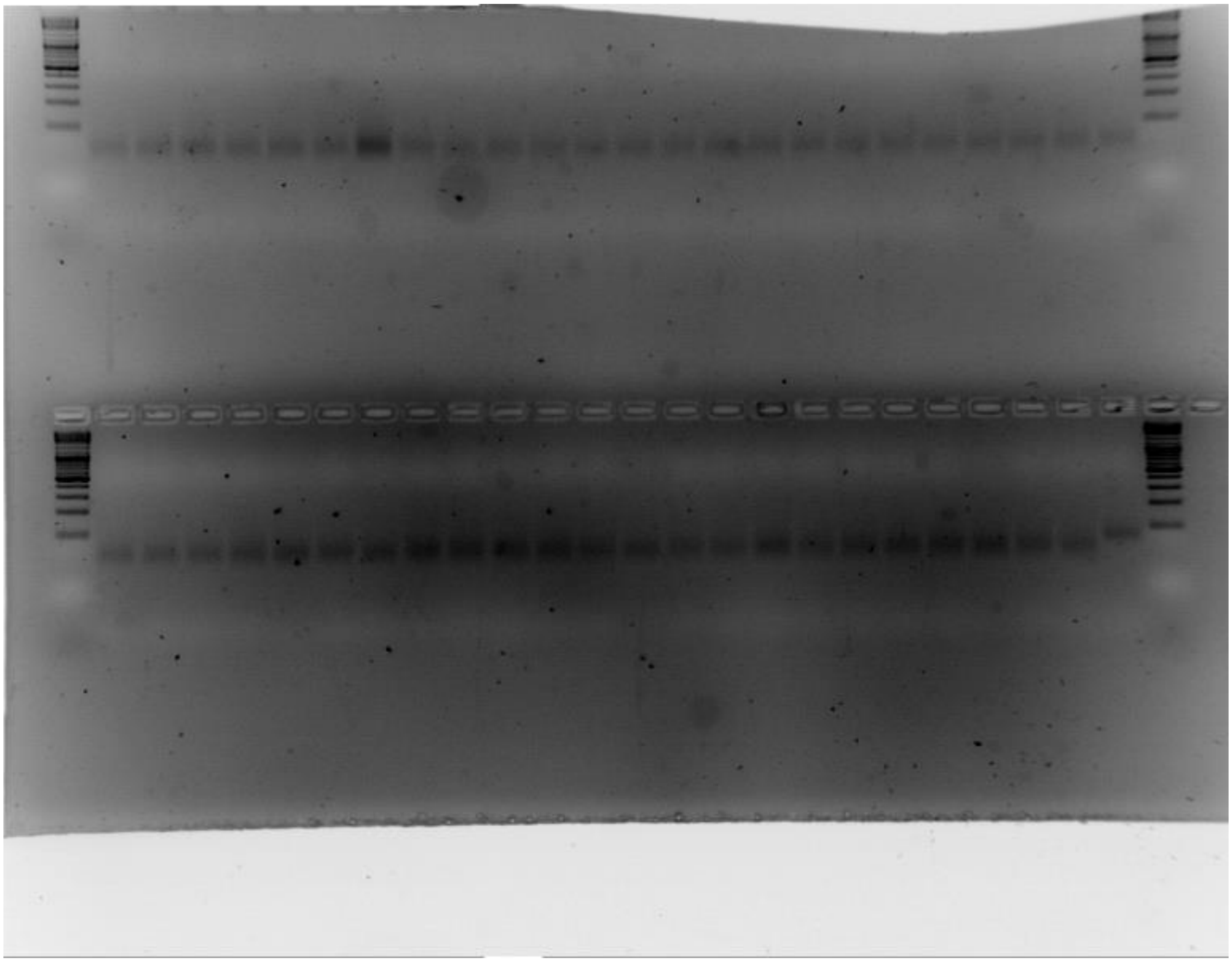

QTL16

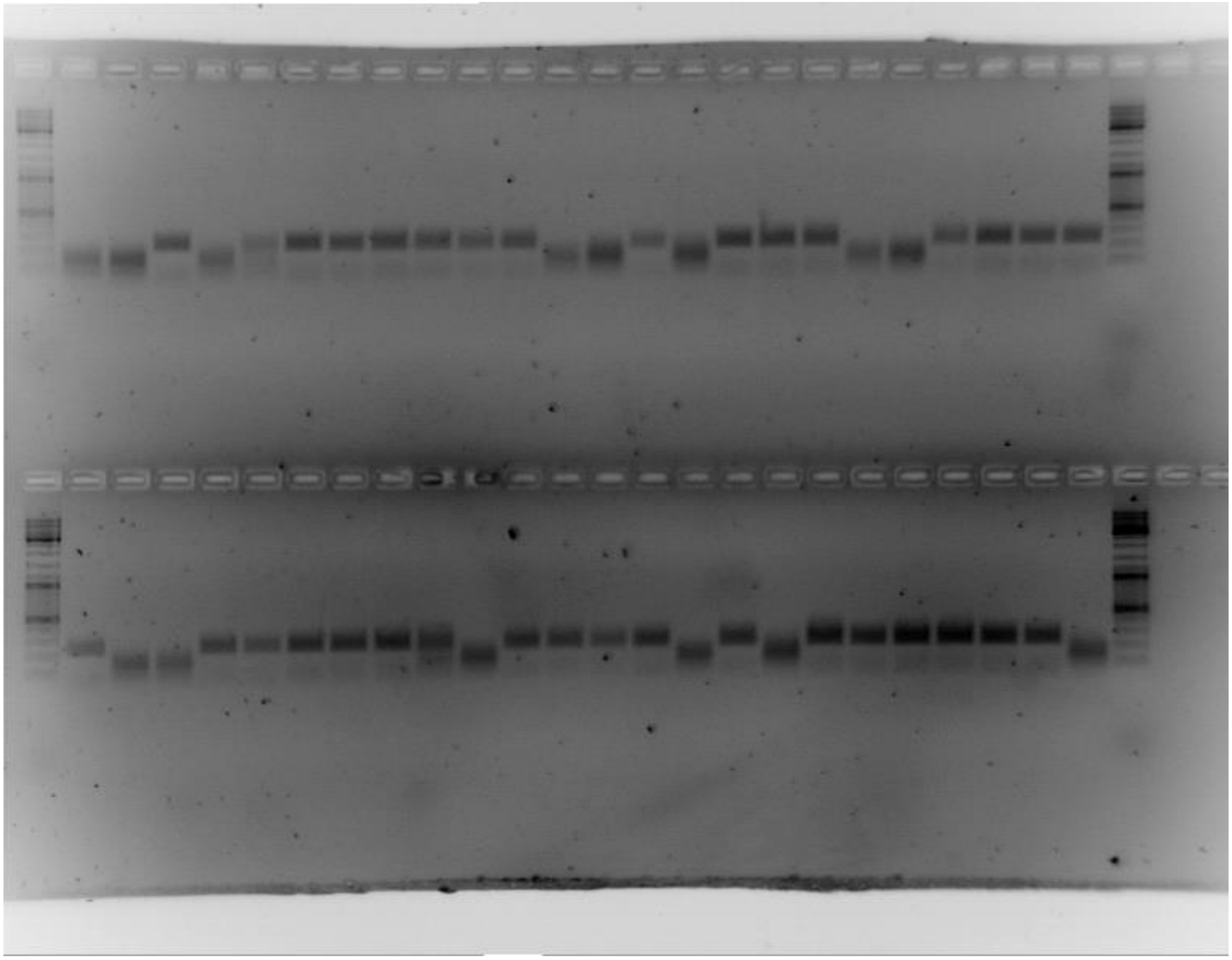

PR_30

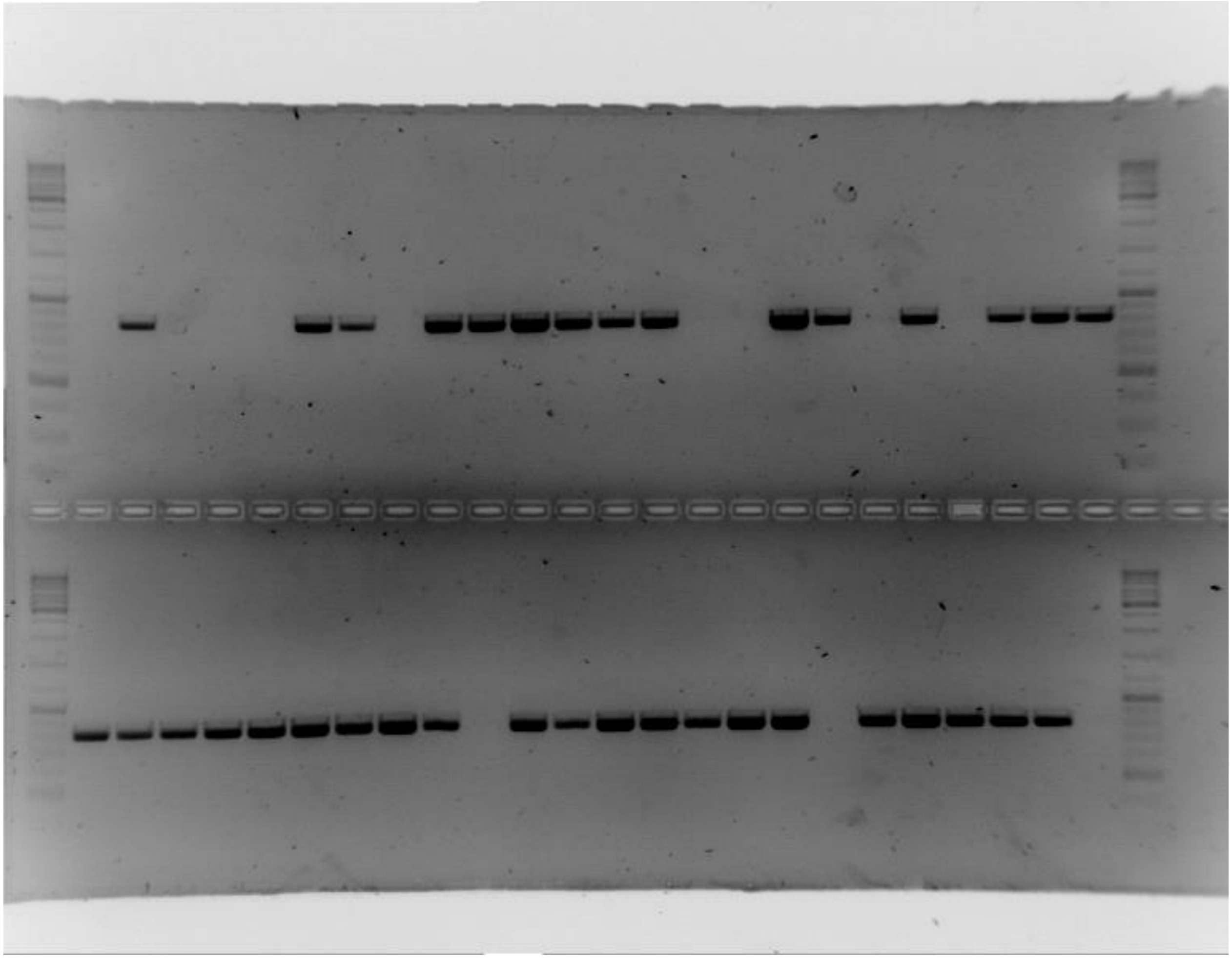

PR_31

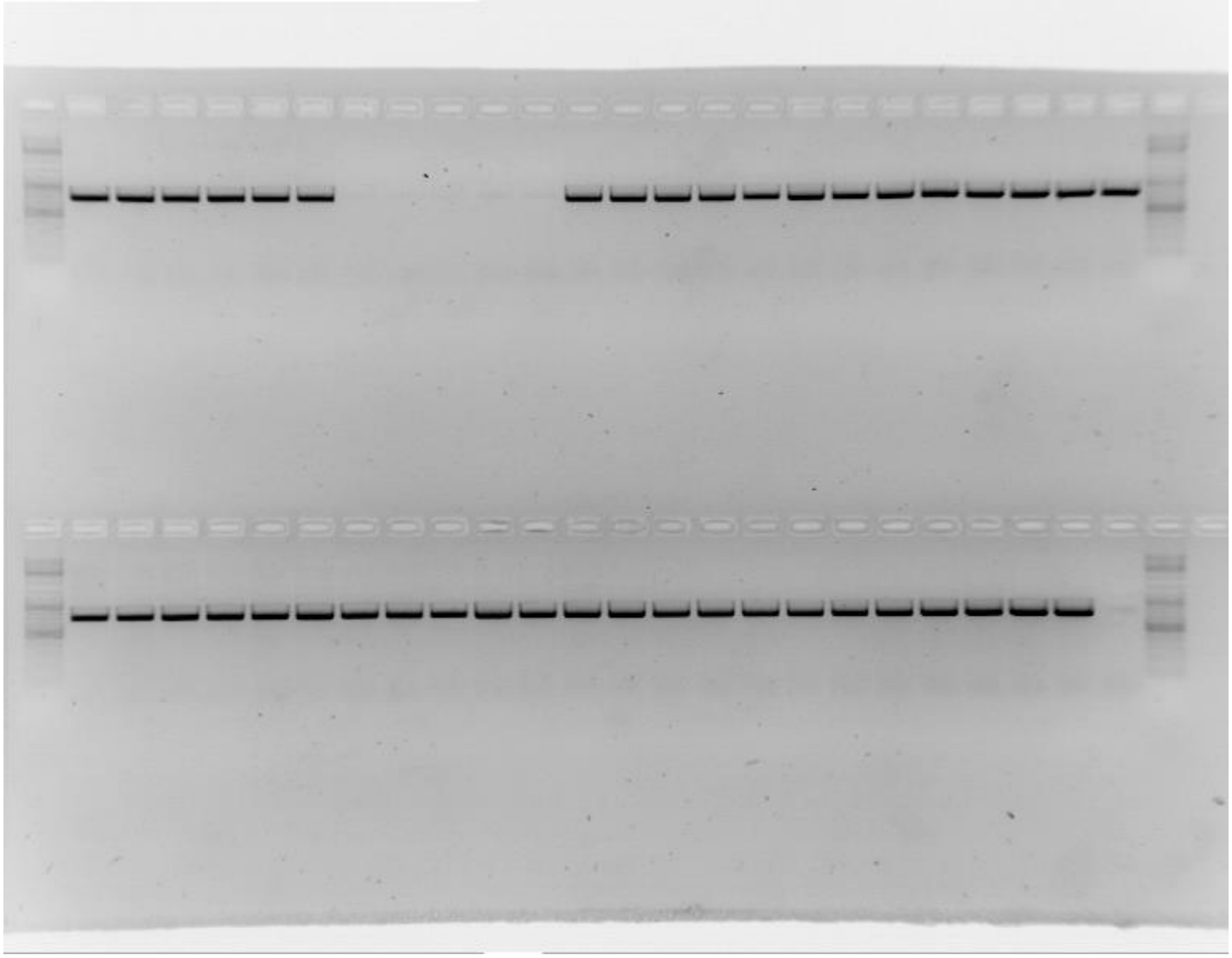

PR_32

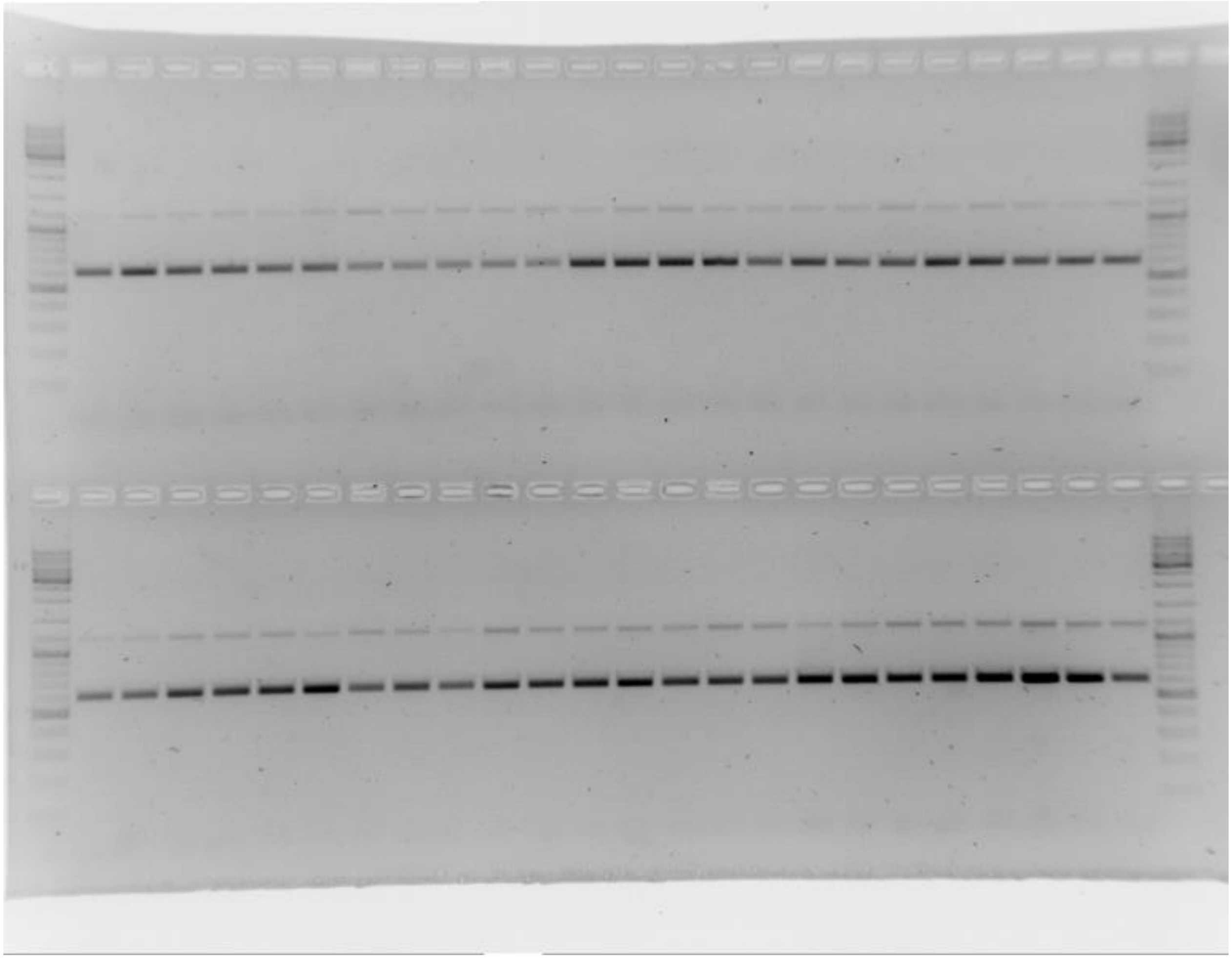

PR_33

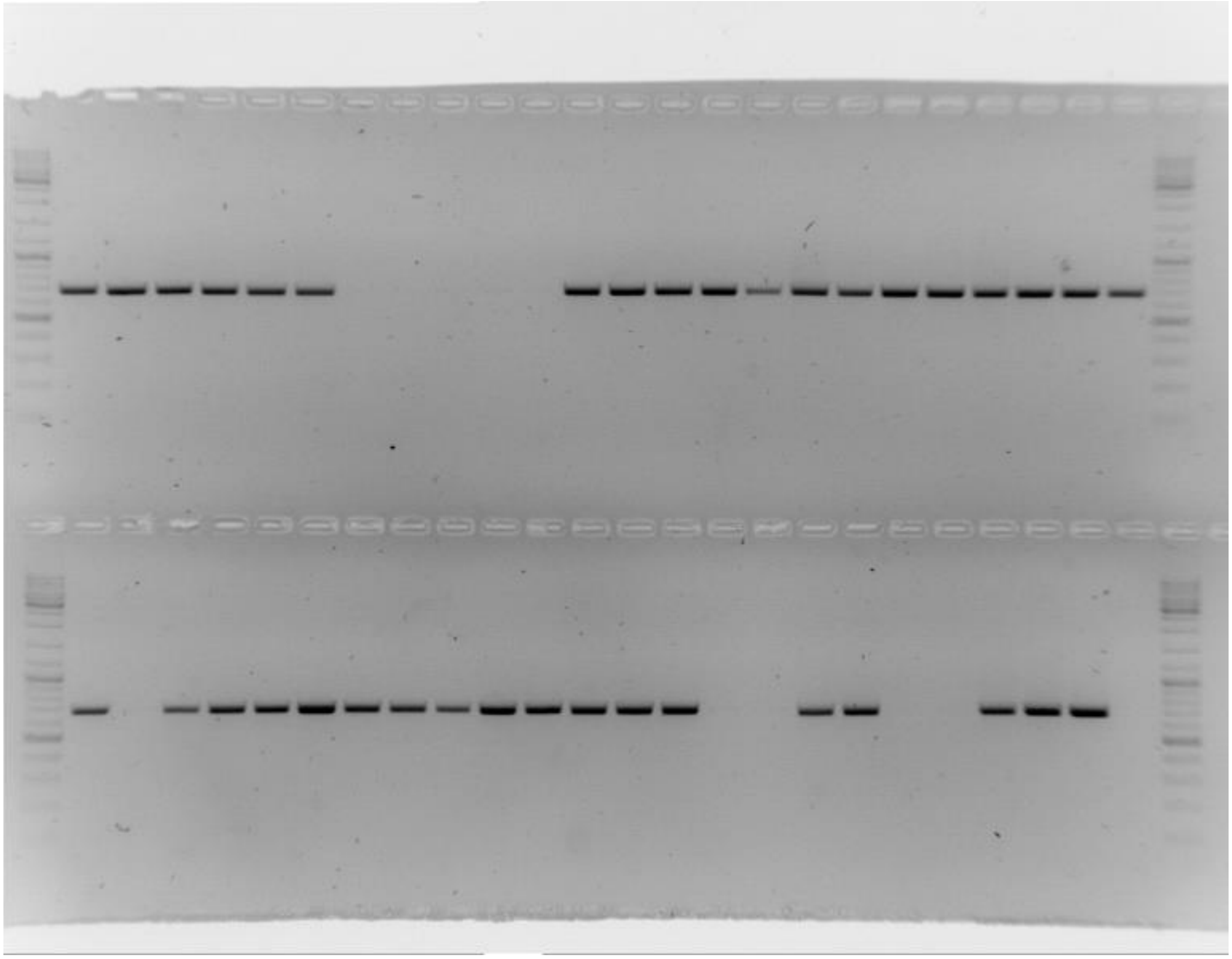

PR_34

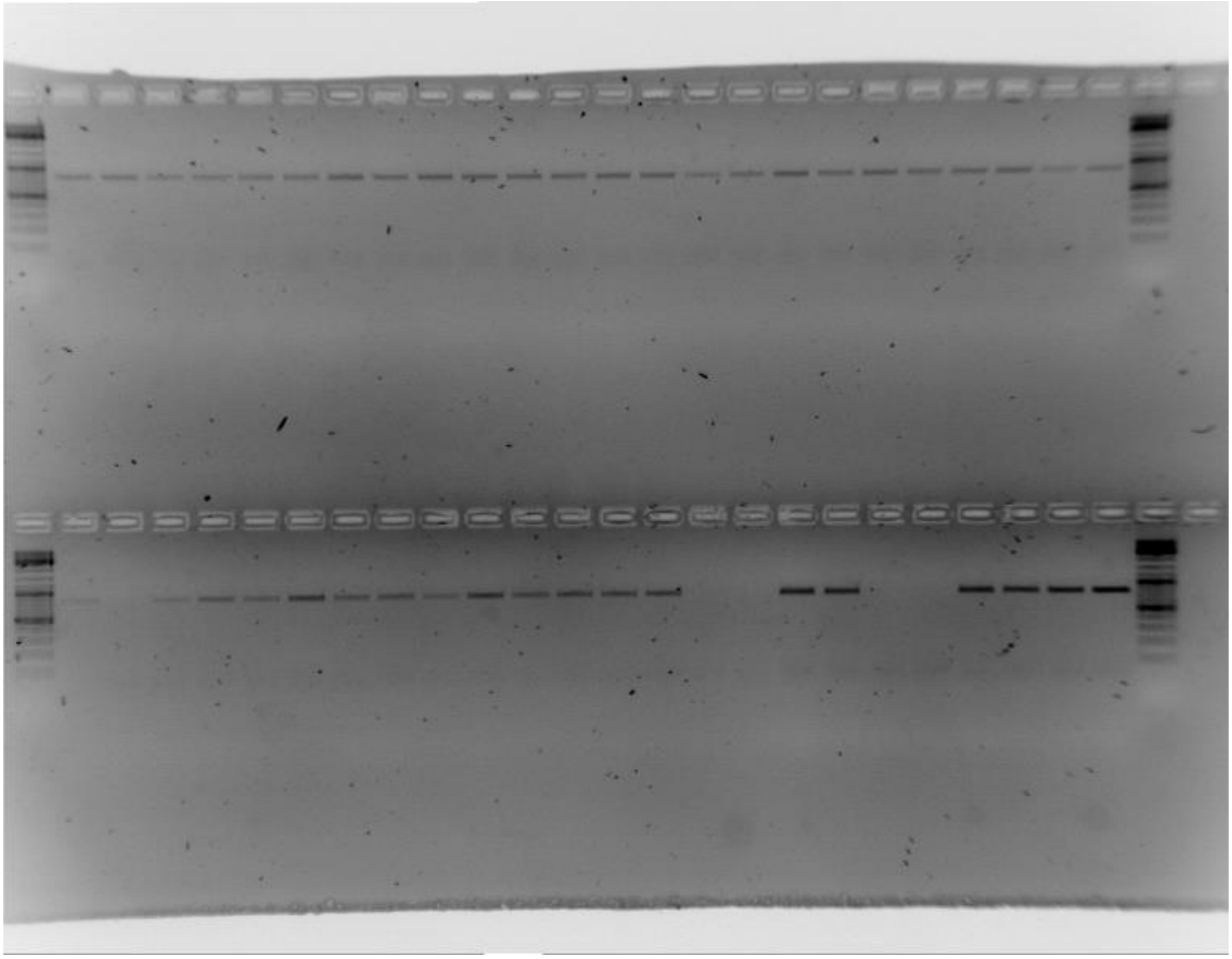

PR_35

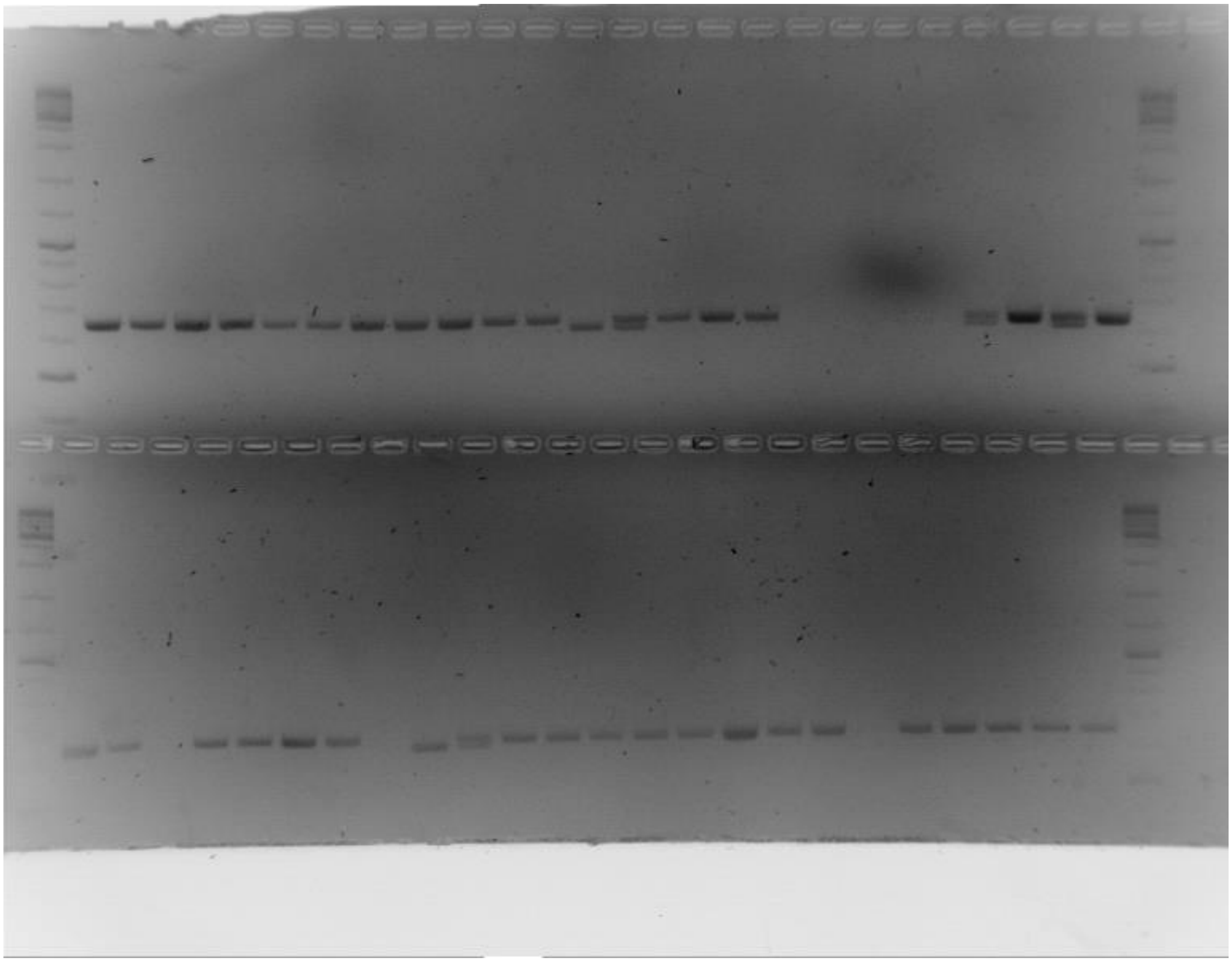

PR_36

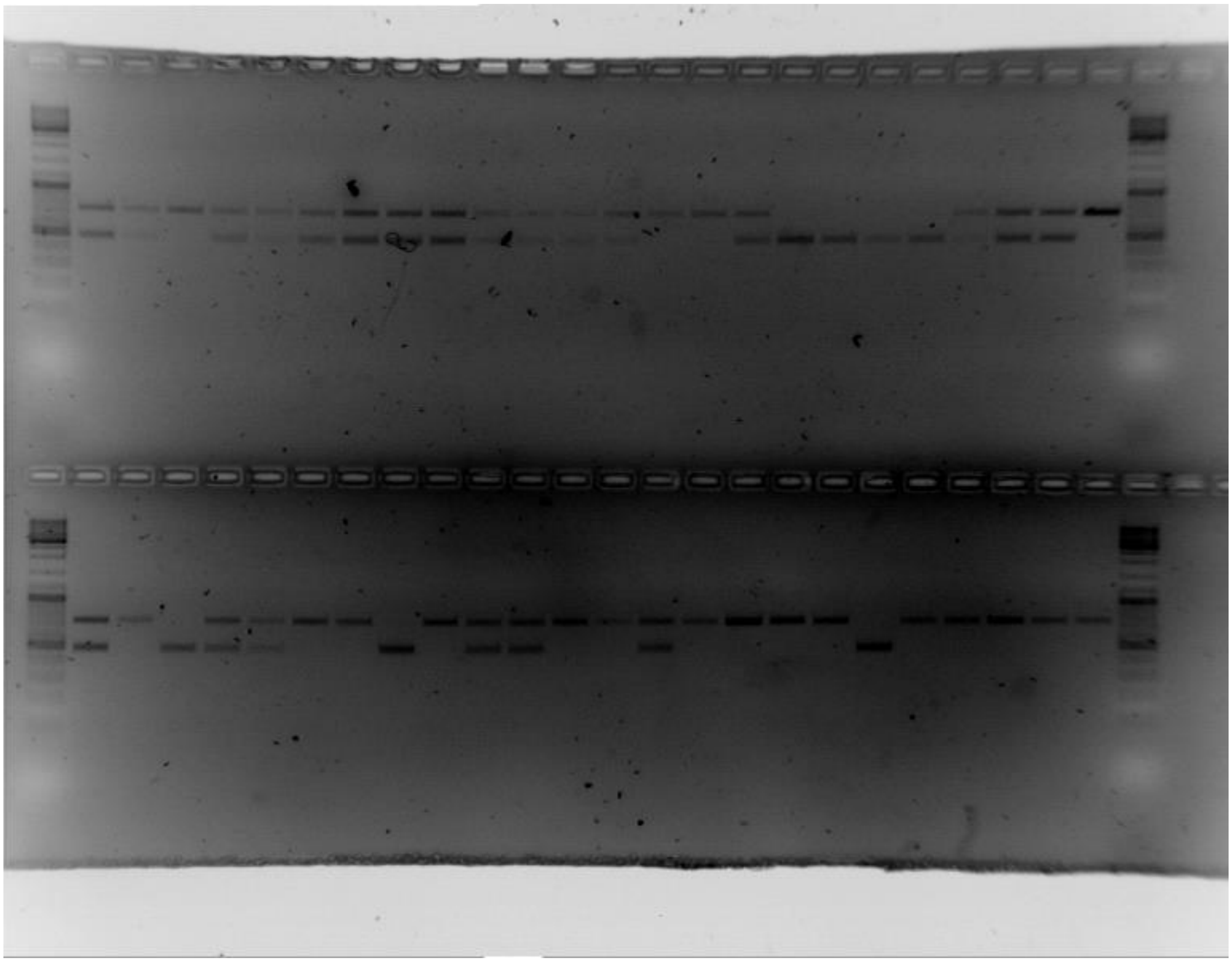

PR_37

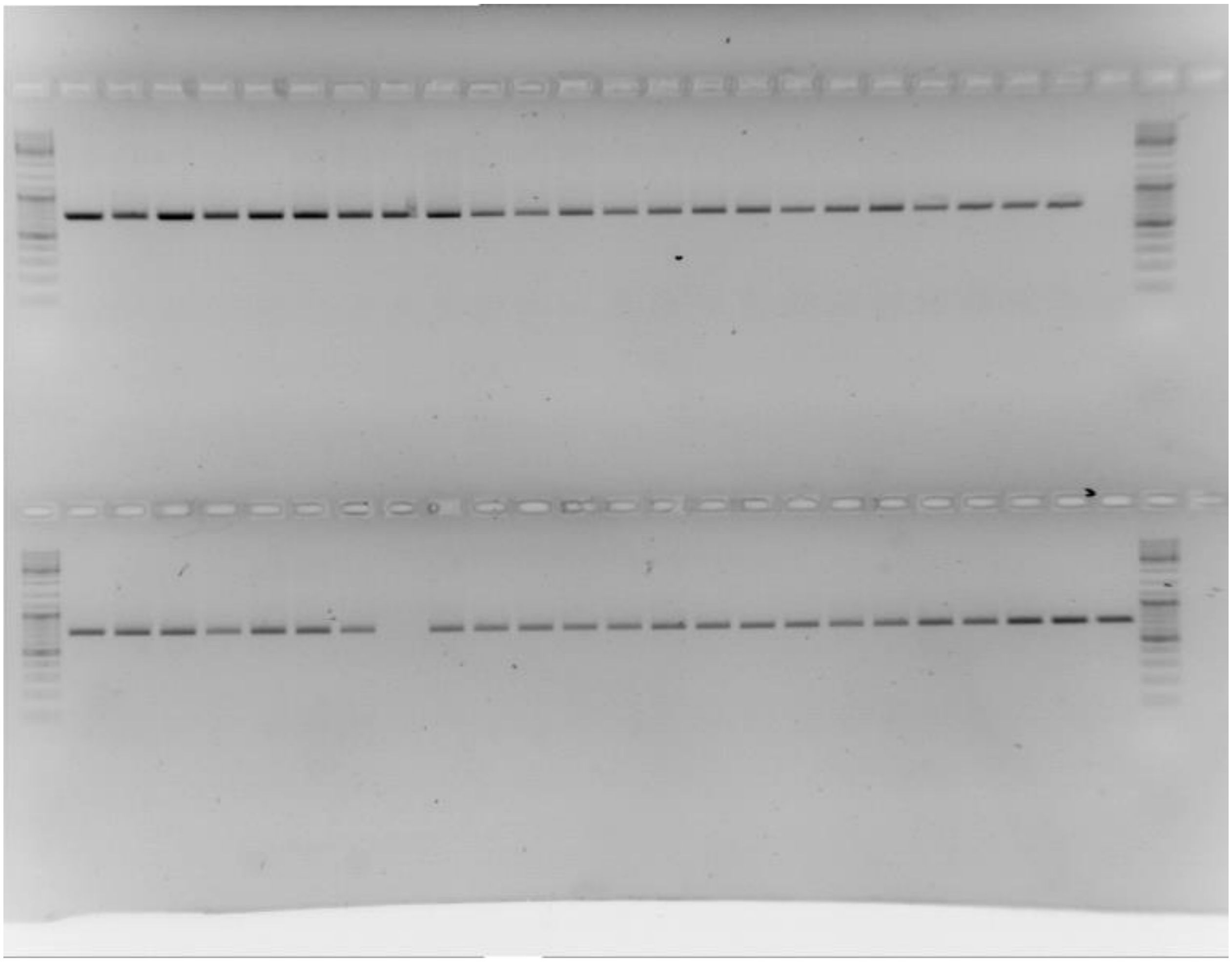

PR_38

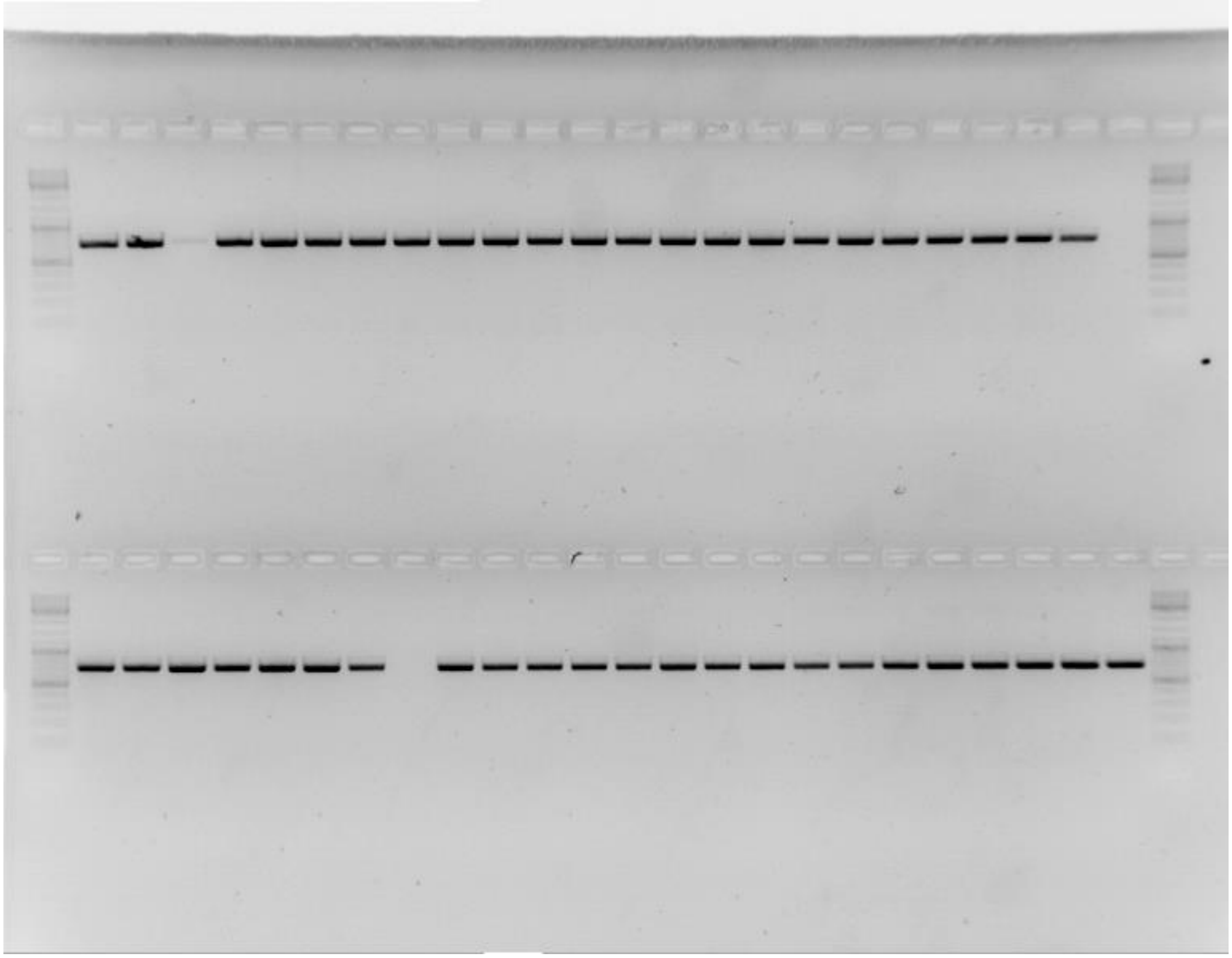

PR_39

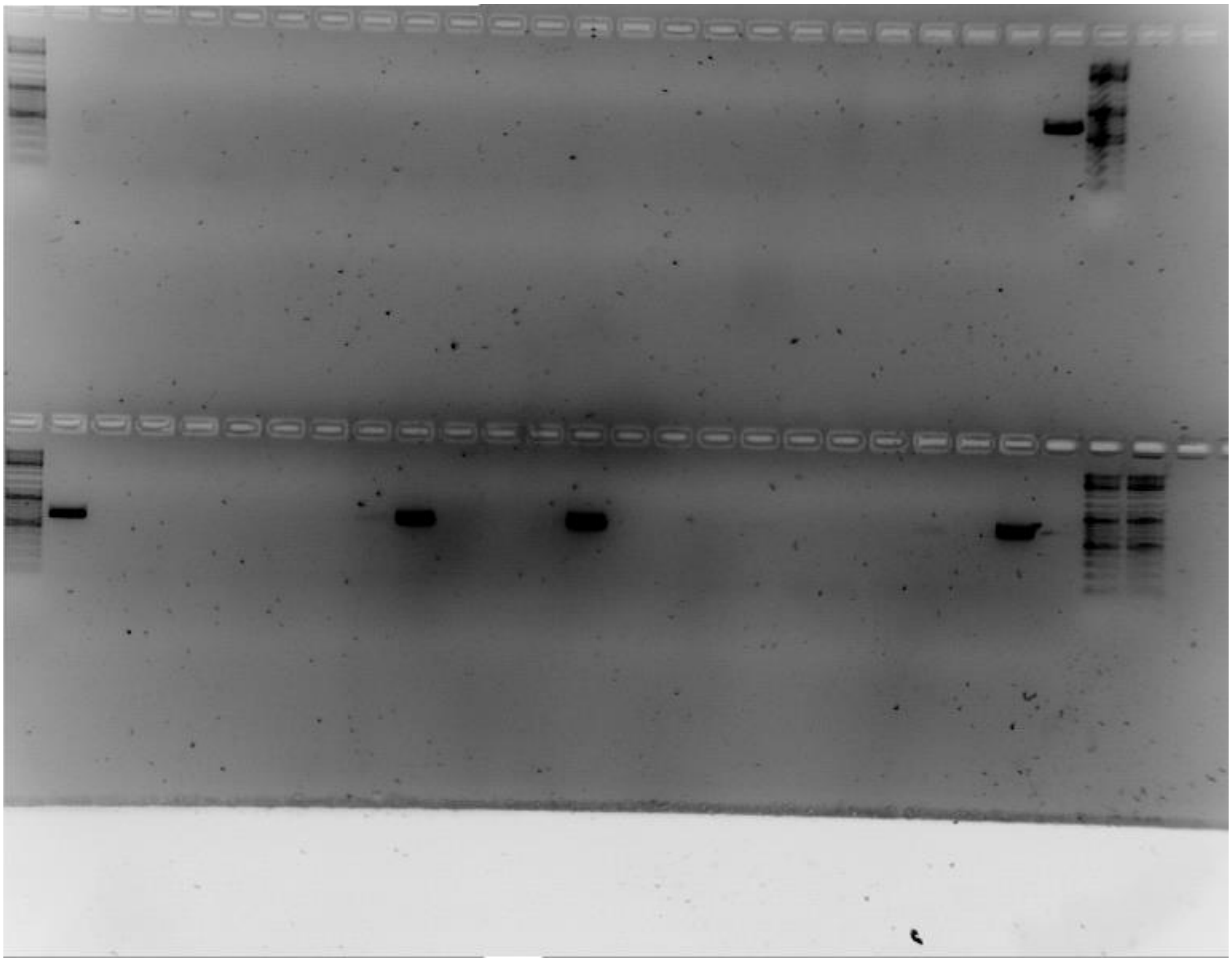

PR_40

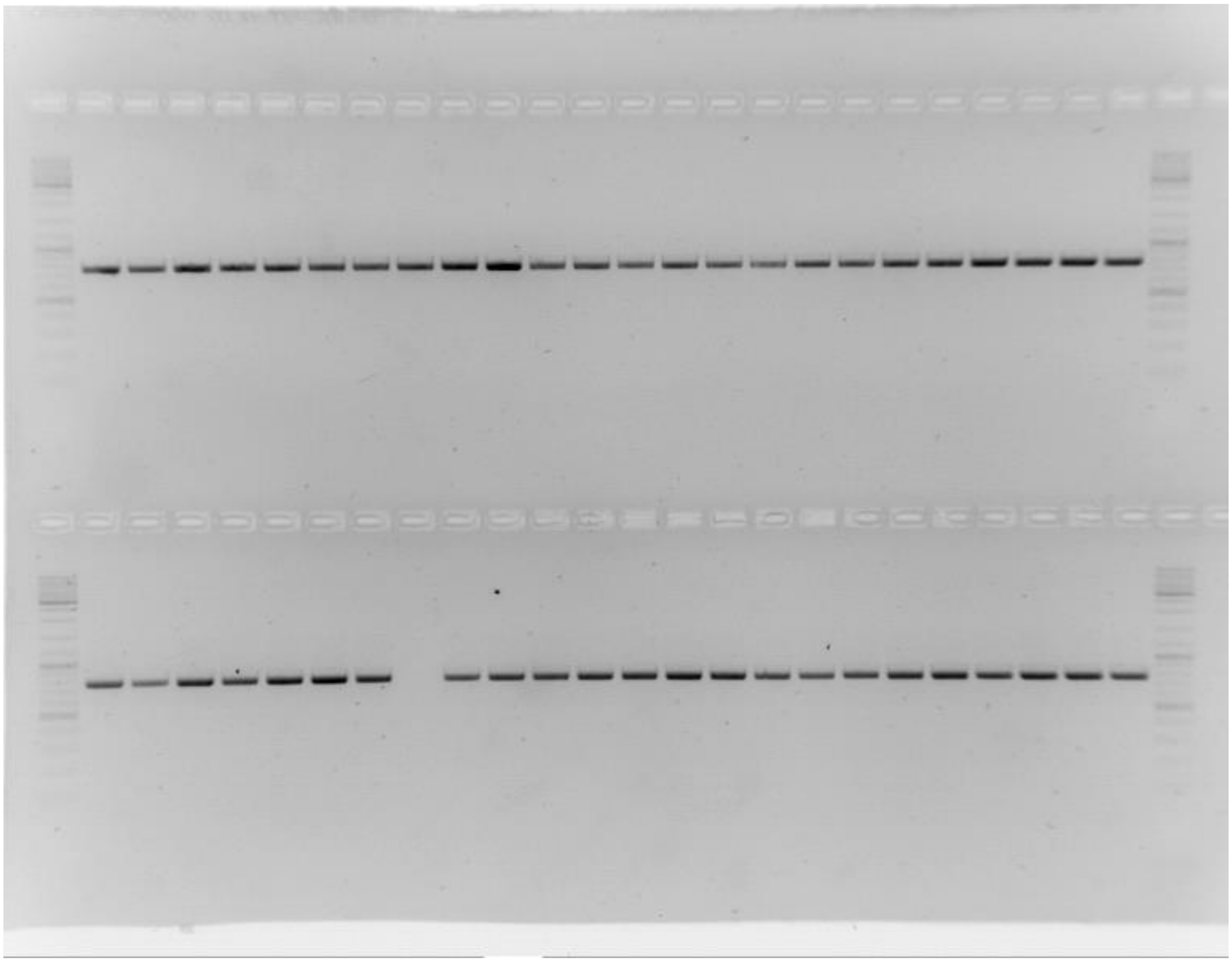

PR_41

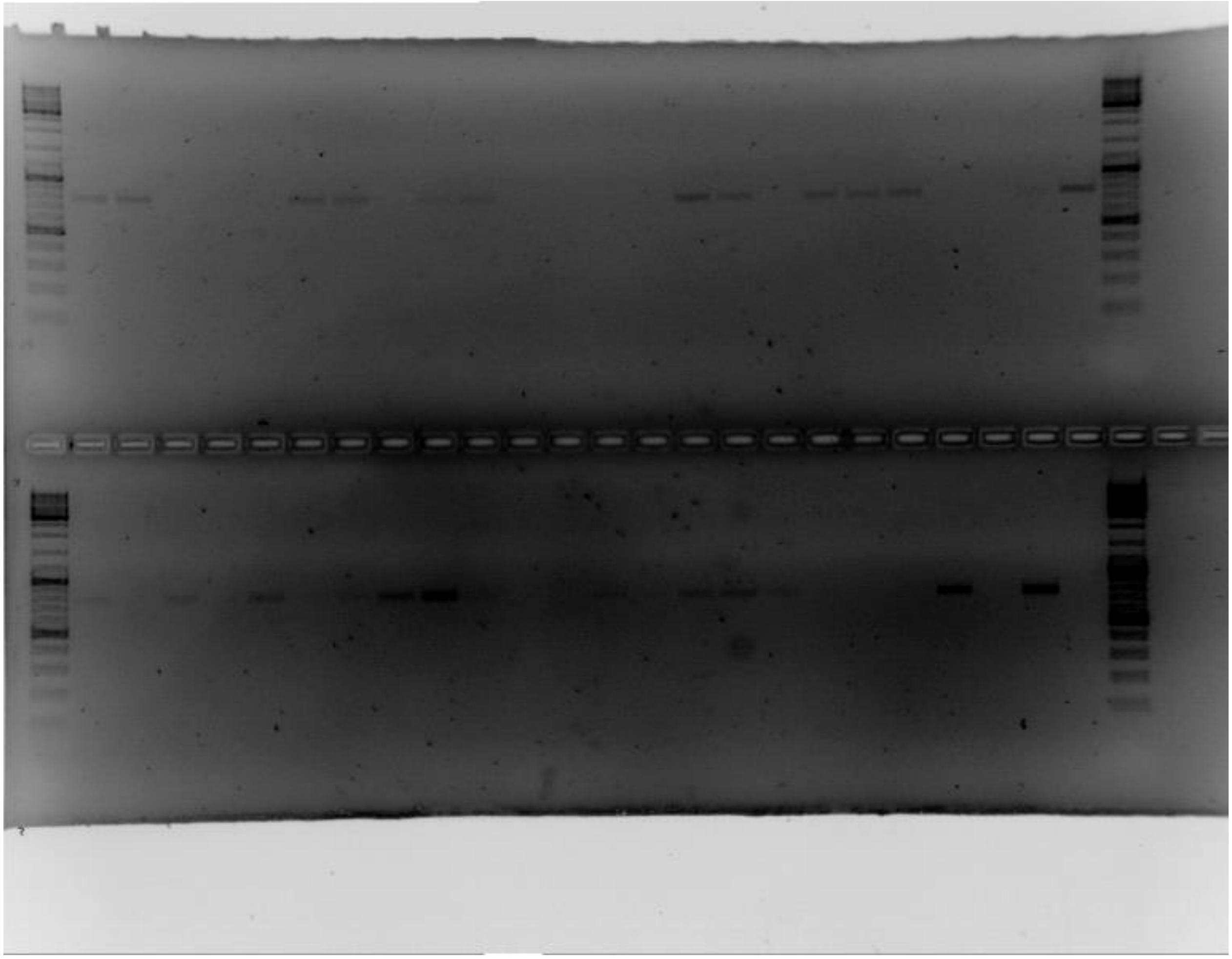

PR_42

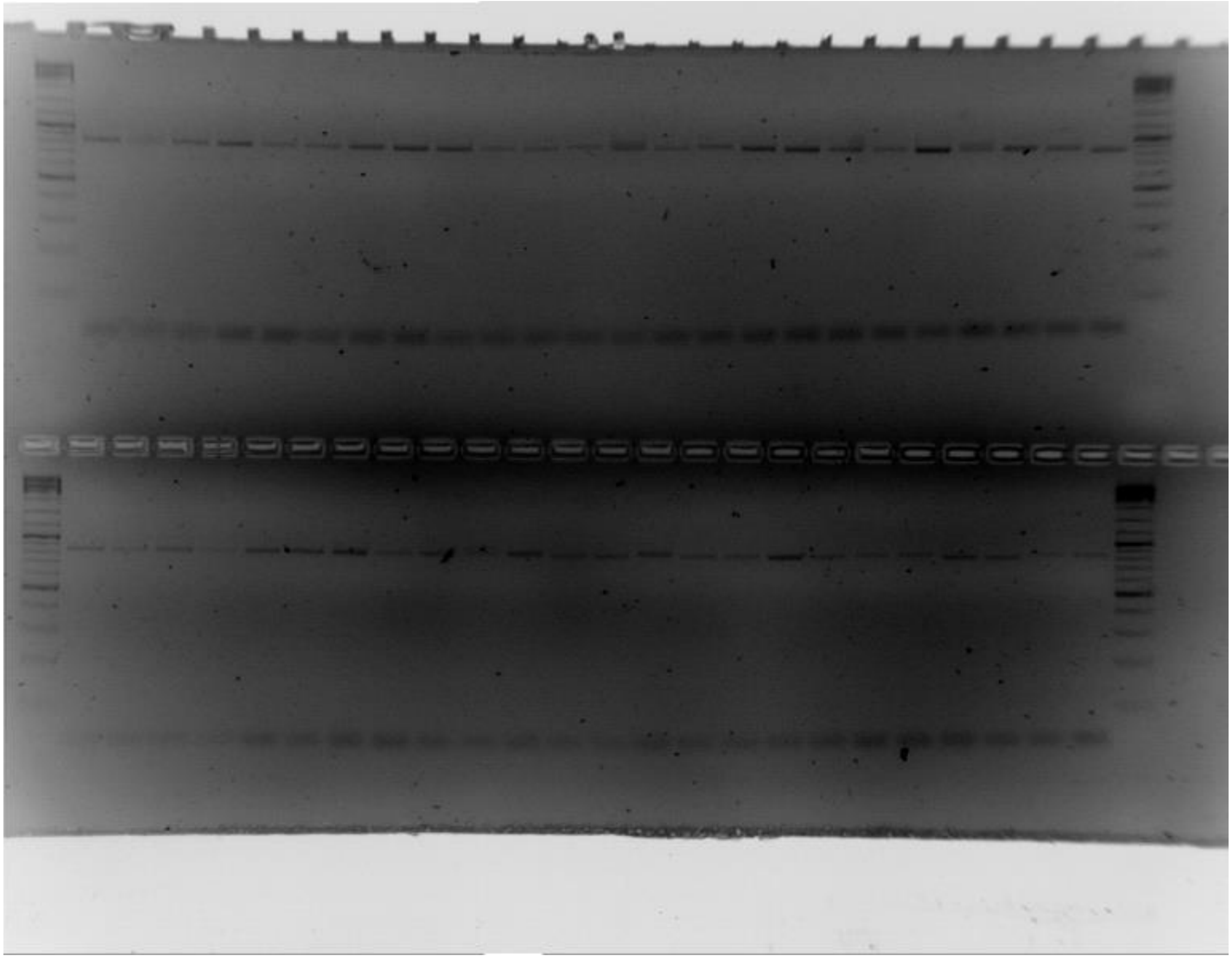

PR_43

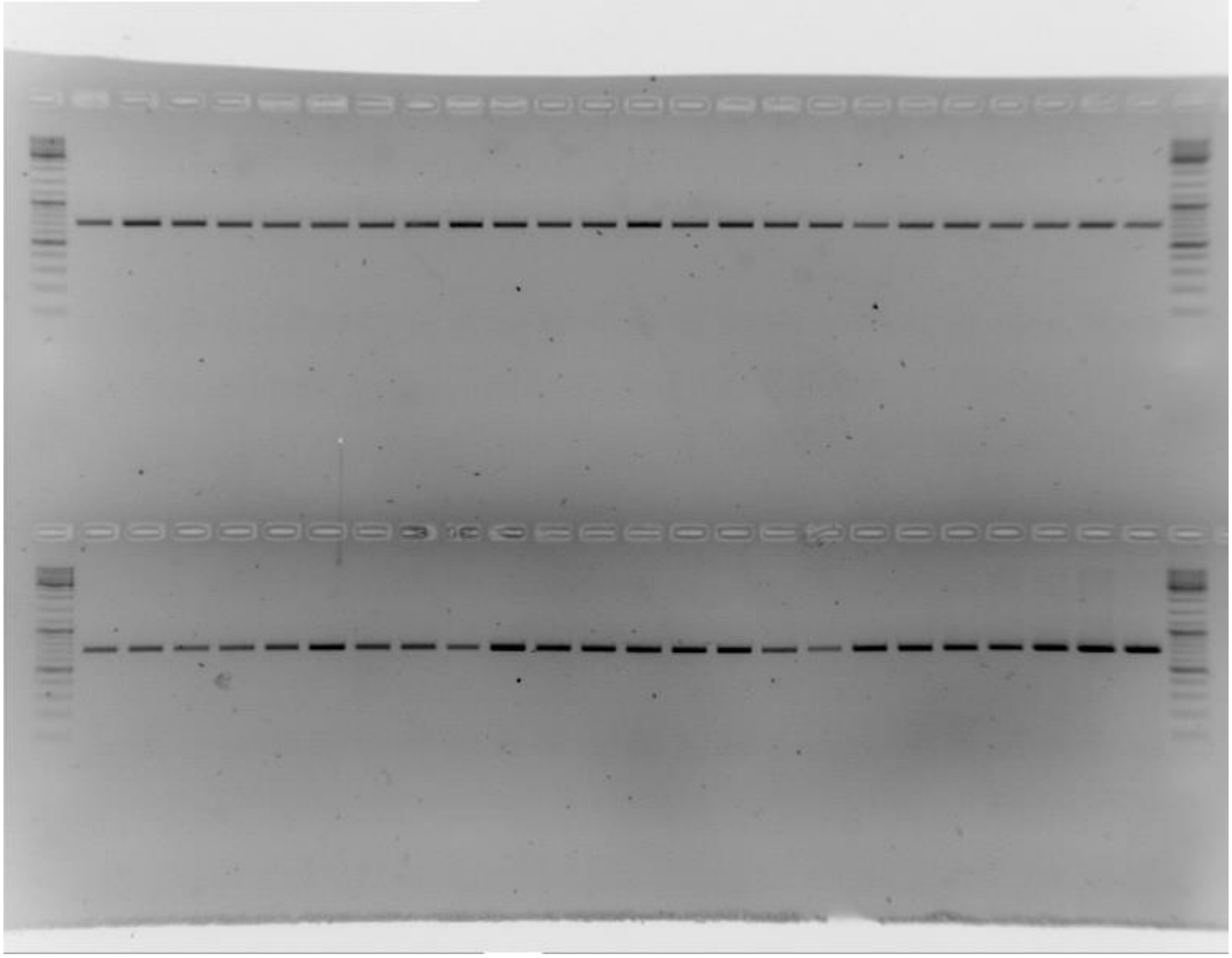

PR_58

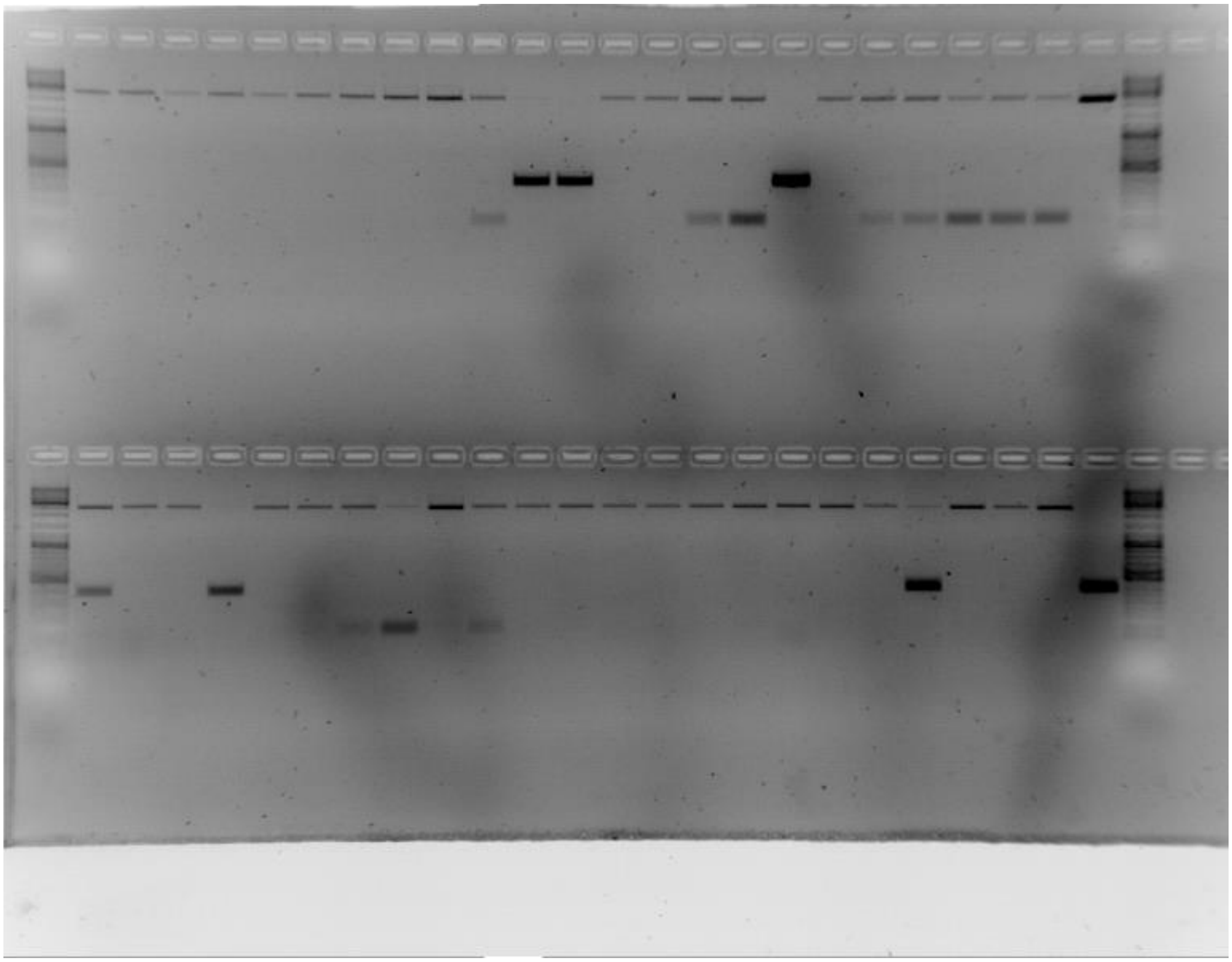

PR_61

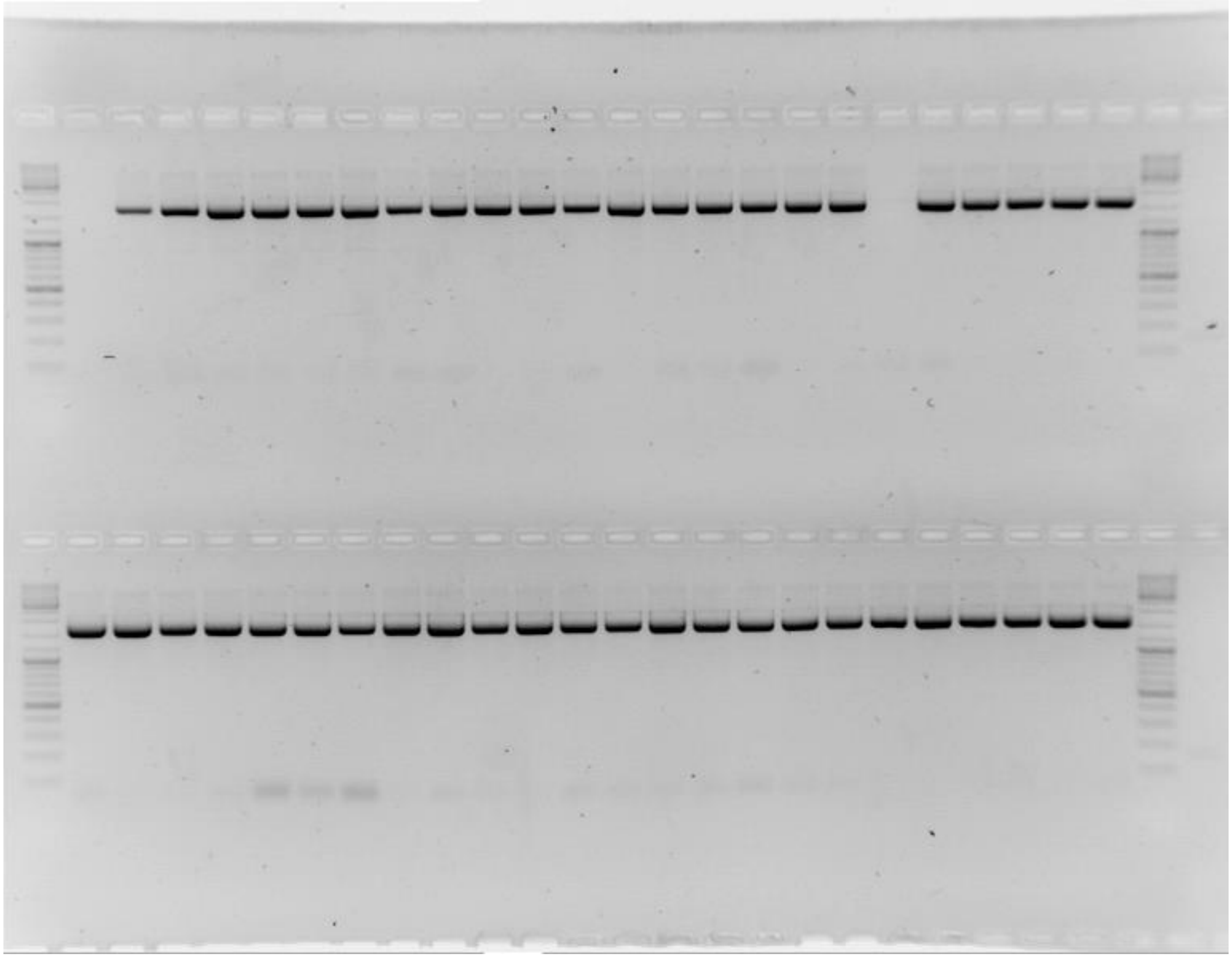

PR_70

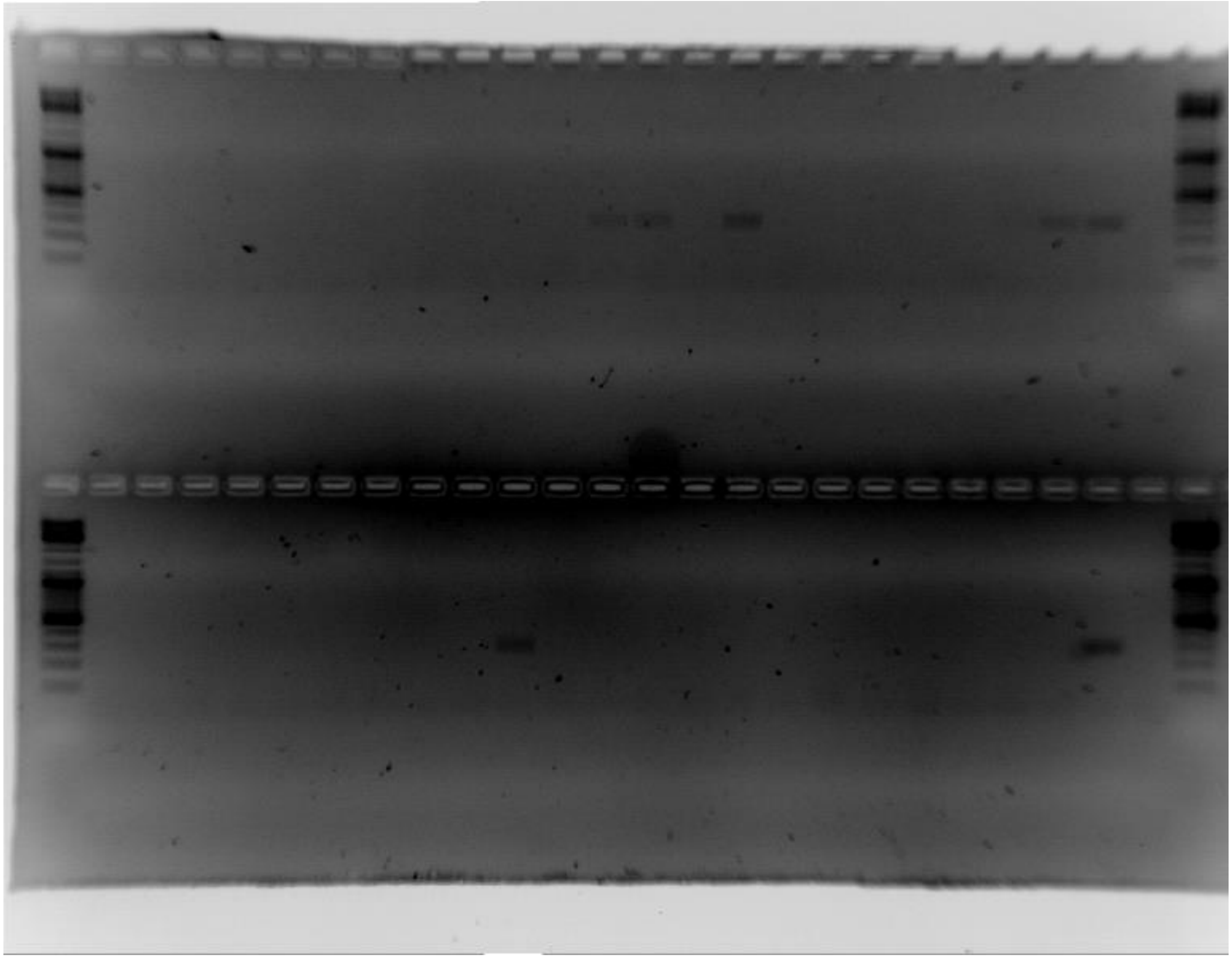

PR_71

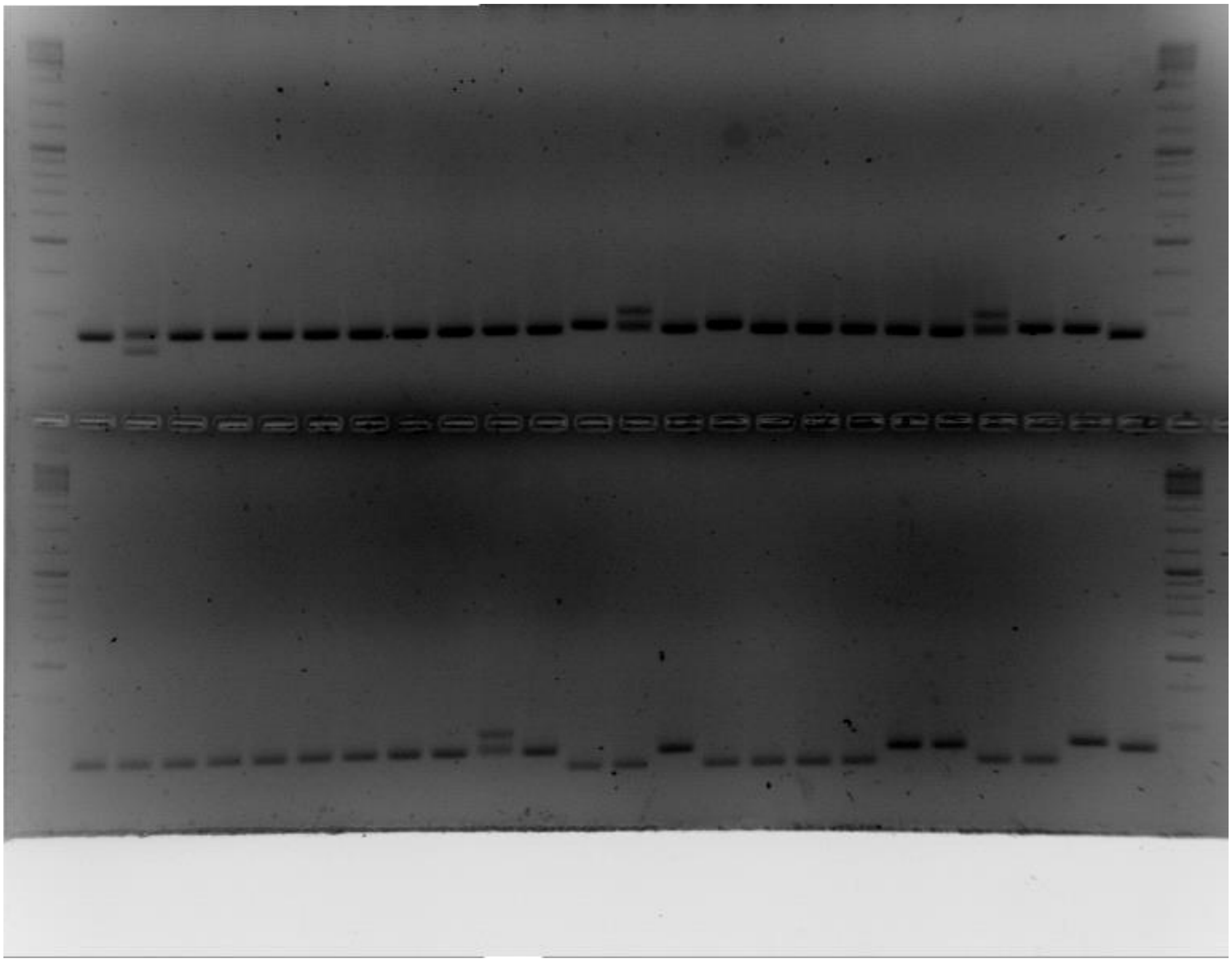

Supplement: Supplementary file 16 — Supplementary Material 16: Supplementary_File_S16.pdf: Full-length original gel images for cropped gels displayed in Supplementary Files. [file 12870_2024_5438_MOESM16_ESM.pdf]
