# Supplementary material for: A GWAS study highlights significant associations between a series of indels in a FLOWERING LOCUS T gene promoter and flowering time in white lupin (Lupinus albus L.)
Source: BMC Plant Biol. 2024 Jul 29;24:722. doi: 10.1186/s12870-024-05438-1 (PMC11285409; doi:10.1186/s12870-024-05438-1)
Supplement: Supplementary file 17 — Supplementary Material 17: The list of supplementary files. [file 12870_2024_5438_MOESM17_ESM.pdf]

The list of supplementary files:

Supplementary_File_S1.xlsx
Supplementary File S1. Phenotypic observations recorded in studied environments for white lupin germplasm diversity panel.

Supplementary_File_S2.xlsx
Supplementary File S2. Total photoperiod (day light) hours from sowing to start of flowering recorded in studied environments for white lupin germplasm diversity panel.

Supplementary_File_S3.xlsx
Supplementary File S3. Cumulative number of growing degree days from sowing to start of flowering (GDDs) recorded in studied environments for white lupin germplasm diversity panel.

Supplementary_File_S4.xlsx
Supplementary File S4. Cumulative vernalization effectiveness of daily temperature (VF) from sowing to start of flowering recorded in studied environments for white lupin germplasm diversity panel.

Supplementary_File_S5.xlsx
Supplementary File S5. DarT-seq and PCR-based markers used for population structure analysis and genome-wide association study in white lupin germplasm diversity panel.

Supplementary_File_S6.pdf
Supplementary File S6. Agarose gel electrophoregrams showing polymorphism of PCR-based markers tagging white lupin flowering time quantitative trait loci (QTLs) from linkage mapping studies.

Supplementary_File_S7.pdf
Supplementary File S7. Agarose gel electrophoregrams showing polymorphism of PCR-based markers developed for white lupin *LalbFTc1* gene promoter indels.

Supplementary_File_S8.pdf
Supplementary File S8. Values of the cross-entropy criterion for a number clusters ranging from K1 to K30.

Supplementary_File_S9.xlsx
Supplementary File S9. Results of population structure analysis in white lupin germplasm diversity panel.

Supplementary_File_S10.xlsx
Supplementary File S10. FDR-corrected P-values and phenotypic effects of markers analyzed in genome-wide association study of white lupin germplasm diversity panel for flowering time in controlled environment and field conditions.

Supplementary_File_S11.xlsx
Supplementary File S11. Results of PCR-based screening of indel polymorphism in promoter regions of *LalbFTa1*, *LalbFTa2*, *LalbFTc1* and *LalbFTc2* genes in white lupin germplasm diversity panel.

Supplementary_File_S12.xlsx
Supplementary File S12. Correlations between studied traits and PCR-based markers tagging indel polymorphism in promoter regions of *LalbFTa1*, *LalbFTa2*, *LalbFTc1* and *LalbFTc2* genes in white lupin germplasm diversity panel.

Supplementary_File_S13.xlsx
Supplementary File S13. The list of genotypes from white lupin germplasm diversity panel analyzed in the study.

Supplementary_File_S14.xlsx
Supplementary File S14. Minimum and maximum air temperature, cumulative growing degree days (GDDs), cumulative vernalization effectiveness of daily temperature (VF) and total photoperiod hours recorded in Lodi, Sanluri, Saint Sauvant and Greenhouse during the course of experiments.

Supplementary_File_S15.xlsx
Supplementary Table S15. Primer sequences of PCR-based markers developed for white lupin *LalbFTa1*, *LalbFTa2*, *LalbFTc1* and *LalbFTc2* gene promoter indels and QTL screening.

Supplementary_File_S16.pdf
Supplementary File S16. Full-length original gel images for cropped gels displayed in Supplementary Files.
